# Elucidation of the *Azotobacter vinelandii* FixABCX Energy Landscape Reveals a Conformationally Gated Flavin-Based Electron Bifurcation

**DOI:** 10.1021/jacs.6c11513

**Published:** 2026-10-29

**Authors:** Nishya Mohamed-Raseek, Dmitriy A. Lukoyanov, J. Luke Findlay, Florence Mus, Seth A. Wiley, Monika Tokmina-Lukaszewska, Farnaz Ghorbani, Carolyn E. Lubner, Brian Bothner, C. Martin Lawrence, Brian M. Hoffman, John W. Peters

**Affiliations:** † Department of Chemistry and Biochemistry, 187873 The University of Oklahoma, Norman, Oklahoma 73019, United States; ‡ Department of Chemistry, 3270 Northwestern University, Evanston, Illinois 60208, United States; § Department of Chemistry and Biochemistry, 33052 Montana State University, Bozeman, Montana 59717, United States; ∥ National Laboratory of the Rockies, 53405 Biosciences Center, Golden, Colorado 80401, United States

## Abstract

Electron bifurcation was first used to describe the electron-transfer events associated with the quinone-cycle (Q-cycle), which occurs in complex III of the electron transport chain. Decades later, flavin-based electron bifurcation (FBEB) was discovered, where flavin cofactors act in analogy to Q to drive the endergonic reduction of ferredoxin (Fd) or flavodoxin (Fld) by NAD­(P)­H. The soil microbe*Azotobacter vinelandii* is known to generate low-potential electrons in the form of reduced Fd or Fld using FBEB via the enzyme complex FixABCX. FixABCX in *A. vinelandii* (*Av*FixABCX) couples the exergonic reduction of coenzyme Q (E = +10 mV) to the endergonic reduction of Fd/Fld (E = ∼−450 mV) during NADH oxidation (E = −320 mV). In this work, we successfully optimized the expression of *Av*FixABCX in *E. coli*. We have employed protein thin-film electrochemistry, UV–vis spectrophotometry, electron paramagnetic resonance (EPR) spectroscopy, and transient absorption (TA) spectroscopy to elucidate the complete energy landscape, and structural characterization methods, cryo-electron microscopy (cryo-EM), small-angle X-ray scattering (SAXS), and cross-linking mass spectrometry (XL-MS) to show the dynamics of the complex energy landscape during *Av*FixABCX electron-transfer catalysis. Our results show that *Av*FixABCX contains two [4Fe-4S] clusters with disparate reduction potentials: the higher-potential cluster participates in the exergonic electron-transfer pathway, whereas the lower-potential cluster is off-pathway. We experimentally determined that *Av*FixABCX undergoes a conformationally gated, reversible FBEB mechanism to simultaneously reduce coenzyme Q and the semiquinone state of Fld during NADH oxidation.

## Graphical abstract

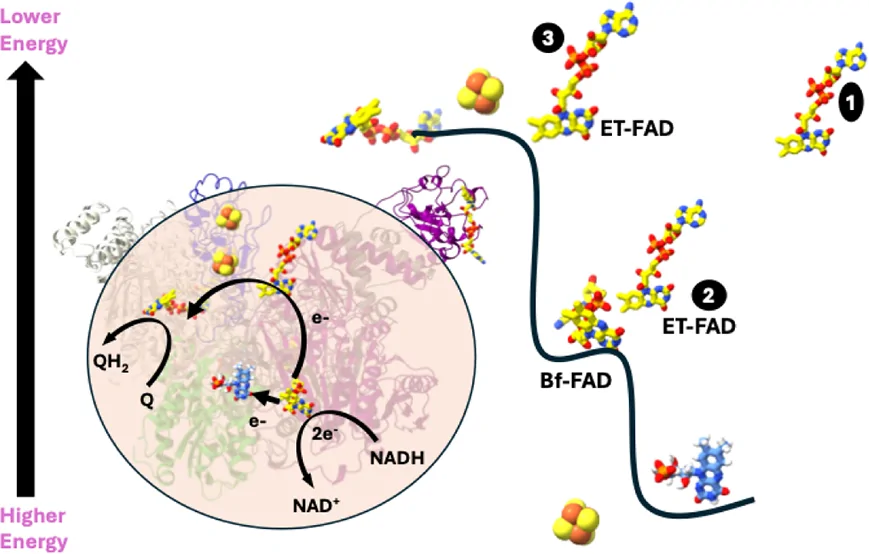

## Introduction

Electron bifurcation (EB) couples endergonic and exergonic reactions by generating an energy-rich reactive intermediate that yields low-potential reducing equivalents to drive cellular reactions such as nitrogen fixation in diazotrophs and methane formation in methanogens.^1,73^ In 1972, Wikström and Berden proposed a bifurcated reaction for quinol oxidation in the cytochrome bc1 complex (or complex III), where Cytochrome *b* and Cytochrome c accept single electrons from quinol (QH_2_).^2^ Later, in 1975, Mitchell followed this model to explain the mitochondrial quinone cycle (Q-cycle), in which reduced Cytochrome *b* is further oxidized by Q, generating a proton-motive force in the electron transport chain (ETC).^3^ The term bifurcation of electron flow was used by Brandt to discuss electron transfer from QH_2_ (*E*_*Q/QH2*_ = ∼+70 mV) to two energetically different electron acceptors, Cytochrome *b*_L_ (*E* = −20 mV) and Rieske [2Fe-2S] (*E* = +290 mV), which is an energy-conserving mechanism leading to proton translocation in the ETC.^4^ In 2008, Buckel and Thauer proposed that electron transfer flavoprotein-butyryl-CoA dehydrogenases (Etf-Bcd) couple oxidation of NADH (*E* = −320 mV) with endergonic electron transfer to low-potential electron acceptors ferredoxin (Fd) or flavodoxin (Fld) (*E* ≤ −450 mV) and exergonic electron transfer to higher-potential crotonyl-CoA reduction to butyryl-CoA (*E* = −10 mV).^1,72^ This mechanism of coupling endergonic and exergonic electron transfer reactions is analogous to the Q-cycle mechanism; however, in Etf-Bcd, it is carried out by a flavin cofactor. Therefore, this mechanism is known as flavin-based electron bifurcation (FBEB). Following the discovery of bifurcating Etfs, four main groups of enzymes have been identified. These are the Etf family, NADPH-dependent reduced Fd:NADP^+^ oxidoreductase (Nfn), heterodisulfides (Hdr), and Bfu family enzymes.^5–12^

Etfs are found in all domains of life. Etfs contain Etfα (or EtfA) and Etfβ (or EtfB) subunits, which tightly interact to form three structural domains: domains I, II, and III. Group I, nonbifurcating, canonical Etfs transfer electrons from dehydrogenases to mitochondrial Etf quinone oxidoreductase (Etf-QO) and contain a single flavin adenine dinucleotide (FAD) in domain II and an AMP in domain III.^13,14^ This FAD is called electron-transfer α-FAD or ET-FAD, whereas in group II Etfs, the second FAD molecule is located at the interface of domains I and III in place of AMP and is found to interact with NADH during electron bifurcation^5,15^ (the domain I, II, and III arrangement is shown in Figure 2B). This FAD is known as bifurcating β-FAD (Bf-FAD).^5^ Spectroscopic redox titration in homologues of bifurcating EtfABs shows that bifurcating flavins suppress the intermediate semiquinone (SQ) form.^5,15,16^ In addition, transient-absorption (TA) spectroscopic data show short-lived SQ states in these EtfABs.^11,17^ *Av*FixABCX belongs to the bifurcating Etf family. FixA and FixB are homologous to EtfB and EtfA, respectively, and FixCX has structural similarities to Etf-quinone oxidoreductase (Etf-QO).^6^

In our previous work, we showed that *Av*FixABCX carries out FBEB by coupling the oxidation of NADH (*E* = −320 mV) to the reduction of the SQ state of flavodoxin (Fld_NSQ_, *E* = −460 mV) in a lower-potential electron transfer pathway and of coenzyme Q (*E* = +10 mV) in the higher-potential electron transfer pathway.^17^ Native mass spectrometry characterization indicated that *Av*FixABCX contains three FADs: a-FAD, referred to as the bifurcating FAD or Bf-FAD, is in FixA; b-FAD, known as the electron transfer FAD or ET-FAD, is in FixB; c-FAD, referred to as the quinone reductase FAD or QR-FAD, is in FixC, and two [4Fe-4S] clusters are in the Fd-like subunit FixX as shown in Figure 1. Chemical cross-linking studies suggested that FixX binds near FixA, and we had proposed that the FixX cluster functions in the low-potential pathway in the reduction of Fld_NSQ_ (Figure 1A). More recently, a cryo-EM structure of a homologue of *Av*FixABCX from *Thermotoga maritima* termed *Tma*EtfABCX revealed that the FixX subunit interacts with ET-FAD in FixB and QR-FAD in FixC, indicating that one FixX [4Fe-4S] cluster is involved in a high-potential electron transfer pathway on the path to Q reduction (Figure 1B).^6^ The result was unanticipated, given the prevalence of 2­[4Fe-4S] clusters. Fds have clusters with negative reducing potentials. To fully rationalize the role of the [4Fe-4S] clusters in *Av*FixABCX, we obtained the cryo-EM structure to determine the cofactor arrangement and employed protein thin-film square-wave voltammetry, UV–vis spectrophotometry, and electron paramagnetic resonance (EPR) to determine the individual reduction potentials of redox cofactors and thereby describe the *Av*FixABCX energy landscape. The *Av*FixABCX energy landscape reveals that a conformationally gated FBEB is required. To determine the conformational equilibria during FBEB, we employed small-angle X-ray scattering (SAXS) and cross-linking mass spectrometry (XL-MS) to characterize structural dynamics in solution. Although many different higher-resolution structures of different conformational states are available in bifurcating Etfs and it has been proposed that a conformational change is required for FBEB, none of these studies have experimentally compared the intrinsic rate of the individual reaction steps to the overall reaction rate of multistep FBEB to prove that FBEB requires a conformational change. In this study, we determined the structure of *Av*FixABCX and associated conformational changes, the reduction potentials of the individual redox cofactors, and the rates of catalytic activity. These data were used to generate the complete energy landscape of *Av*FixABCX and propose a mechanism for the conformationally gated FBEB.

**Figure 1:**
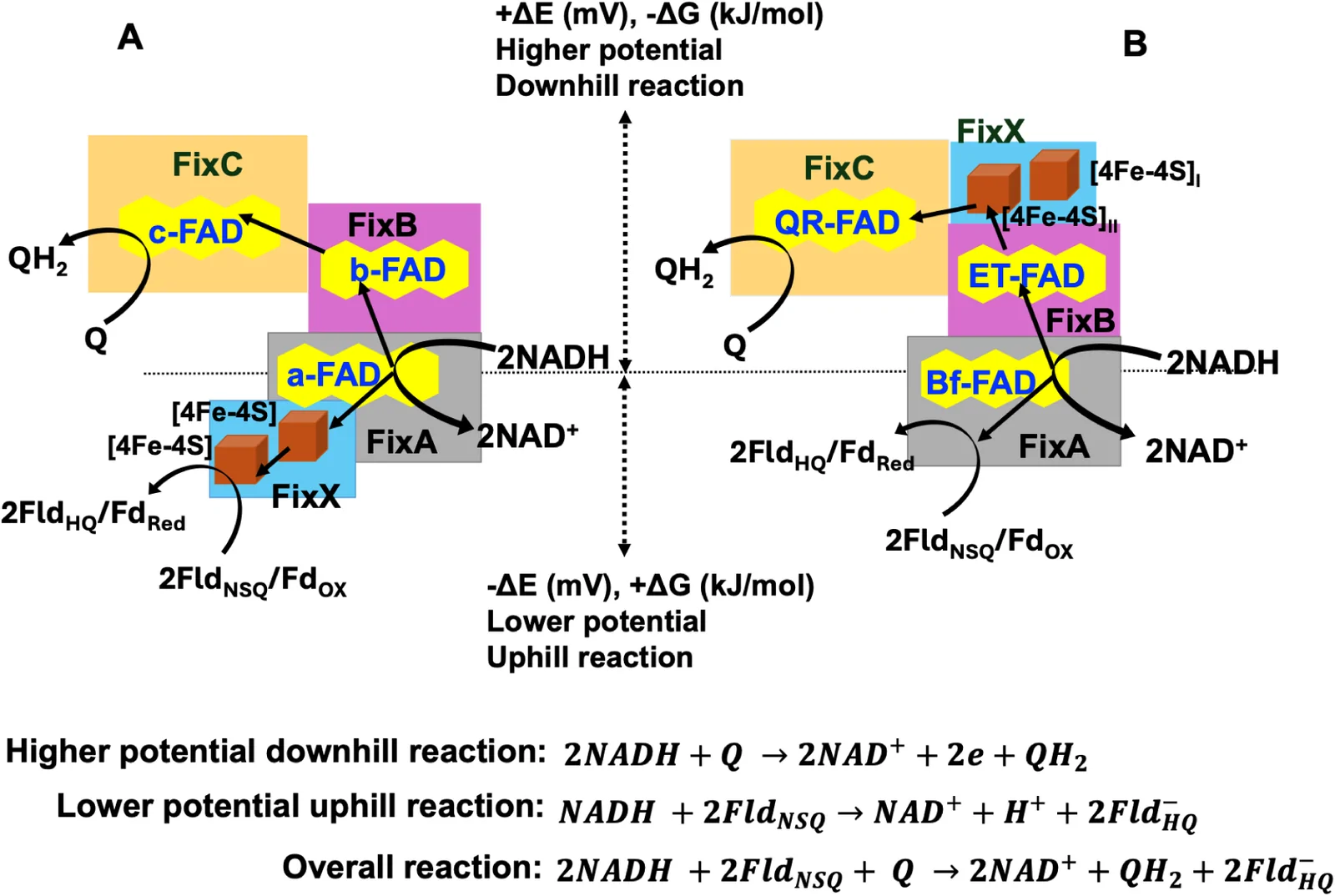
Cartoon diagram of subunit arrangement in *Av*FixABCX and the proposed FBEB pathway. A) Nomenclature of cofactors, subunit arrangement, and the proposed FBEB mechanism in *Av*FixABCX as predicted using the cross-linking mass spectrometry-based structural model in the previous study.^17^ B) Nomenclature of cofactors, subunit arrangement, and the proposed FBEB mechanism in *Av*FixABCX as reported in homologue *Tma*EtfABCX; solid arrows show the direction of electron transfer from NADH to Q in the higher-potential pathway and from NADH to Fld_NSQ_/Fd in the lower-potential pathway. During cycle 1, 2e are transferred from NADH to Bf-FAD (a-FAD); then these two electrons are directed into two different pathways: one electron goes into the higher-potential pathway to Q, and the other electron goes to Fld/Fd in the lower-potential pathway. Two cycles of electron transfer from NADH are expected to reduce a Q molecule and either two Fld_NSQ_ molecules or one Fd.

## Results and Discussion

### Protein Characterization and Cofactor Quantification

Heterologous expression in BL21­(DE3)-*ΔiscR* strain under anaerobic conditions and anaerobic protein purification of *Av*FixABCX yield ∼1.7 mg/L of soluble protein containing a full complement of FAD and FeS clusters. Flavin and Fe quantification^18^ indicated ratios of 3.17 ± 0.21:8.09 ± 0.09:1, FAD:Fe:protein. Protein yield and purity were significantly improved compared to previous work using homologous expression.^17^ Size-exclusion chromatography (SEC) analysis shows three separate peaks, all of which contain *Av*FixABCX. The first peak, which eluted in the void volume of the column (∼670 kDa), was attributed to aggregation. The second (∼260 kDa) and third (∼158 kDa) peaks were consistent with octamer and tetramer complexes based on protein standards (Supporting Information Figure S6A; mass spectrometry analysis was also carried out to confirm the presence of *Av*FixABCX). To better interrogate the properties of all three FADs and two [4Fe-4S] clusters separately, the *Av*FixX subunit was heterologously expressed in *E. coli* with a C-terminal MBP tag. Expression of *Av*FixX in both BL21­(DE3) and BL21­(DE3)-*ΔiscR* cells yielded equivalent protein yields of 2 mg/L in growth culture. However, the Protein:Fe ratio increased from 1:0.35 to 1:2.5 when expressed in BL21­(DE3)-*ΔiscR* strain. FeS cluster reconstitution of purified *Av*FixX with FeCl_3_ and Li_2_S/Na_2_S overnight at 4 °C increased the protein:Fe ratio to 1:8.29 ± 0.17. Anaerobic overexpression of *Av*FixAB and *Av*FixCX in BL21­(DE3)-*ΔiscR* strain yielded proteins with low cofactor occupancy. However, reconstitution with FAD and FeS clusters resulted in full cofactor occupancy.

### Cryo-EM Structure of *Av*FixABCX and Cofactor Arrangements

To address the discrepancy between the *Av*FixABCX structural model^17^ and the *Tma*EtfABCX cryo-EM structure, we determined the higher-resolution structure of *Av*FixABCX. The structure of the as-purified *Av*FixABCX was determined at 4.7 Å resolution by cryo-EM single-particle analysis. The RMSD between the corresponding protomers of *Tma*EtfABCX and *Av*FixABCX is 1.0 Å, and between the overall octamers is 1.9 Å. Similar to *Tma*EtfABCX^6^ (PDB ID 7KOE), the *Av*FixABCX hetero-octamer (ABCX)_2_ forms a 2-fold symmetric U-shaped structure (Figure 2A). The interaction between the protomers is mediated exclusively by the FixC subunits, which, along with FixX at each corner, form the base of the U. The N-terminal domain of FixB (domain I, residues 10–225) lies at the apex of each arm, with domain II (residues 235–359) extending down the arm and interacting slightly with FixX. The FixA major domain (residues 1–235) is present in the middle of the arms, juxtaposed between the Fd-like fold of FixX and domain I of FixB, with which there is a tight interaction. The C-terminus of FixA then extends toward and binds to domain II of FixB via a two-helix bundle. Importantly, FixB domain II and the bound C-terminal arm of FixA appear slightly displaced from the rest of the FixA/FixB structure, to which they are tethered by two hinge-like linkers (FixA: 239–249; FixB: 226–234). Instead, domain II is mainly stabilized through its interaction with FixX, just above the corners of the U-shaped structure. Density for three FAD cofactors and two [4Fe-4S] clusters is clear in each *Av*FixABCX protomer. In contrast, density is not observed for Q, and this may be due to disorder in this region of the structure. Specifically, FixC also lacks strong density for the “horizontal helices” C and D (HHc, HHd) implicated in Q binding. However, the 2.9 Å resolution *Tma*EtfABCX shows density for menaquinone (MQ) binding in the EtfC subunit.^6^

**Figure 2:**
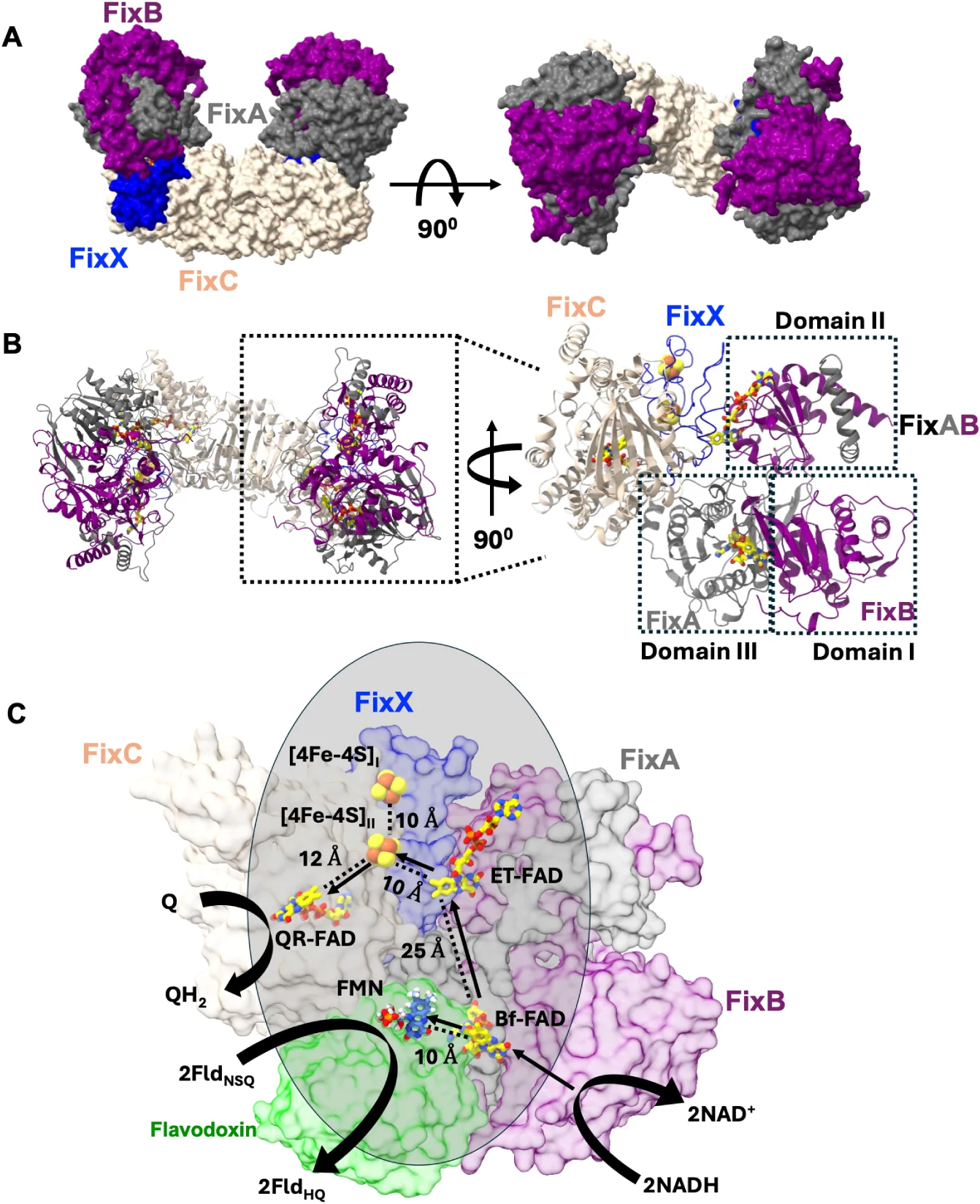
Cryo-EM structure of the *Av*FixABCX octamer (PDB ID 9ZQZ). A) Space-filling model of the octamer in two different orientations, with FixA, FixB, FixC, and FixX in gray, purple, beige, and blue, respectively. The two FixC subunits in each of the two ABCX protomers interact at the base of the U-shaped structure to form a 2-fold (C2) symmetric assembly. B) Ribbon diagram of the space-filling model shown in (A); 90-degree rotation of one protomer to show the domains in FixAB and the cofactor arrangement in each subunit. C) Cofactor arrangement in each protomer, edge-to-edge distance between the cofactors, and proposed electron-transfer pathway during FBEB: ET-FAD is in domain II (gray and purple) and Bf-FAD lies at the FixAB interface between domains I (purple) and III (gray), FADs are in yellow with heteroatom coloring, while Fe and S in [4Fe-4S] clusters are shown in orange and yellow, respectively; solid arrow shows the proposed electron transfer pathway from NADH to higher potential Q and lower-potential Fld_NSQ_ during FBEB; dotted lines show distances between the cofactors in *Av*FixABCX; and *Av* flavodoxin (*Av*Fld_NSQ_) bound *Av*FixABCX model (*Av*Fld is shown in green) was generated using ClusPro, where a 10 Å distance between the FMN of Fld (FMN is in blue with heteroatom coloring) and Bf-FAD was observed.

The relative physical location of the five redox cofactors is critical to function. The adenine-ribose-diphosphate moieties of the Bf-FAD are bound by FixA, with density for the isoalloxazine ring at the interface between FixA and domain I of FixB (Figure 2B). This Bf-FAD interacts with NADH during EB. The ET-FAD is bound by domain II of FixB, with the isoalloxazine ring at the FixB/FixX interface. The quinone reductase FAD (QR-FAD) is found in FixC close to the putative quinone-binding site, while the two FeS clusters termed [4Fe-4S]_I_ and [4Fe-4S]_II_ are located roughly 10 Å apart within FixX (Figure 2C). In this position, the [4Fe-4S]_II_ cofactor is ∼10 Å from the ET-FAD and 12 Å from the QR-FAD, and thus in position to facilitate an efficient electron transfer from ET-FAD through [4Fe-4S]_II_ to QR-FAD for subsequent reduction of Q to QH_2_. [4Fe-4S]_I_, however, is not at a favorable electron-transfer distance from both ET-FAD and QR-FAD. Therefore, electron transfer between ET-FAD and QR-FAD is only possible through [4Fe-4S]_II_. In contrast to the constellation of ET-FAD, [4Fe-4S]_II_, and QR-FAD, which appears primed for electron transfer, the distance from Bf-FAD to ET-FAD is ∼25 Å, which is a long distance for direct electron transfer. Thus, as in TmaEtfABCX,^6^ our cryo-EM single-particle analysis revealed a structure that appears conformationally inhibited with regard to electron transfer between Bf-FAD and ET-FAD.

### UV–Visible Spectroscopic Characterization of *Av*FixABCX and Reductive Titration with NADH

To experimentally validate whether the electron pathway implicated in the structures is feasible, we evaluated the properties of the five redox cofactors (three FADs and two [4Fe-4S]). The absorption spectrum of *Av*FixABCX consists of absorption peaks of two [4Fe-4S] clusters at 420 nm and three FADs at 375 nm and 450 nm.^19,20^ As-purified *Av*FixABCX shows partially reduced spectral features of bound FAD cofactors as the absorbance of band 2 at 365 nm (Supporting Information, Figure S6F, red spectrum) is higher than the absorbance of band 1 at 450 nm; however, air oxidation increases the intensity of both band 1 and band 2 of FAD while shifting the band 2 absorbance to 375 nm (Supporting Information, Figure S6F, blue spectrum), signifying that the FAD cofactors are being oxidized. After the acidic heat denaturation of *Av*FixABCX, the spectrum of free FADs without FeS cluster contributions exhibits the typical FAD band 1 at 450 nm and band 2 at 375 nm (Supporting Information, Figure S6F, green spectrum).

Reductive titration with NADH is one of the ways to confirm the presence of redox-active cofactors in the air-oxidized *Av*FixABCX samples. Adding a half-equivalent amount of NADH to the one equivalent amount of protein concentration provides 1 eq of electrons. Figure 3A shows the UV–visible spectra of the changes in absorbance during the sequential reduction of *Av*FixABCX with NADH. Each spectrum, following the first purple spectrum, was collected after 0.5 eq of NADH was added. According to the proposed electron bifurcation mechanism shown in Figure 2C, the order of reduction expected is Q, QR-FAD, [4Fe-4S]_II_, ET-FAD, and Bf-FAD. QR-FAD, ET-FAD, and Q undergo two 1e^–^ reductions to reduce the oxidized (OX) forms of FAD and Q to the semiquinone (SQ) form, which requires 0.5 eq of NADH.

**Figure 3:**
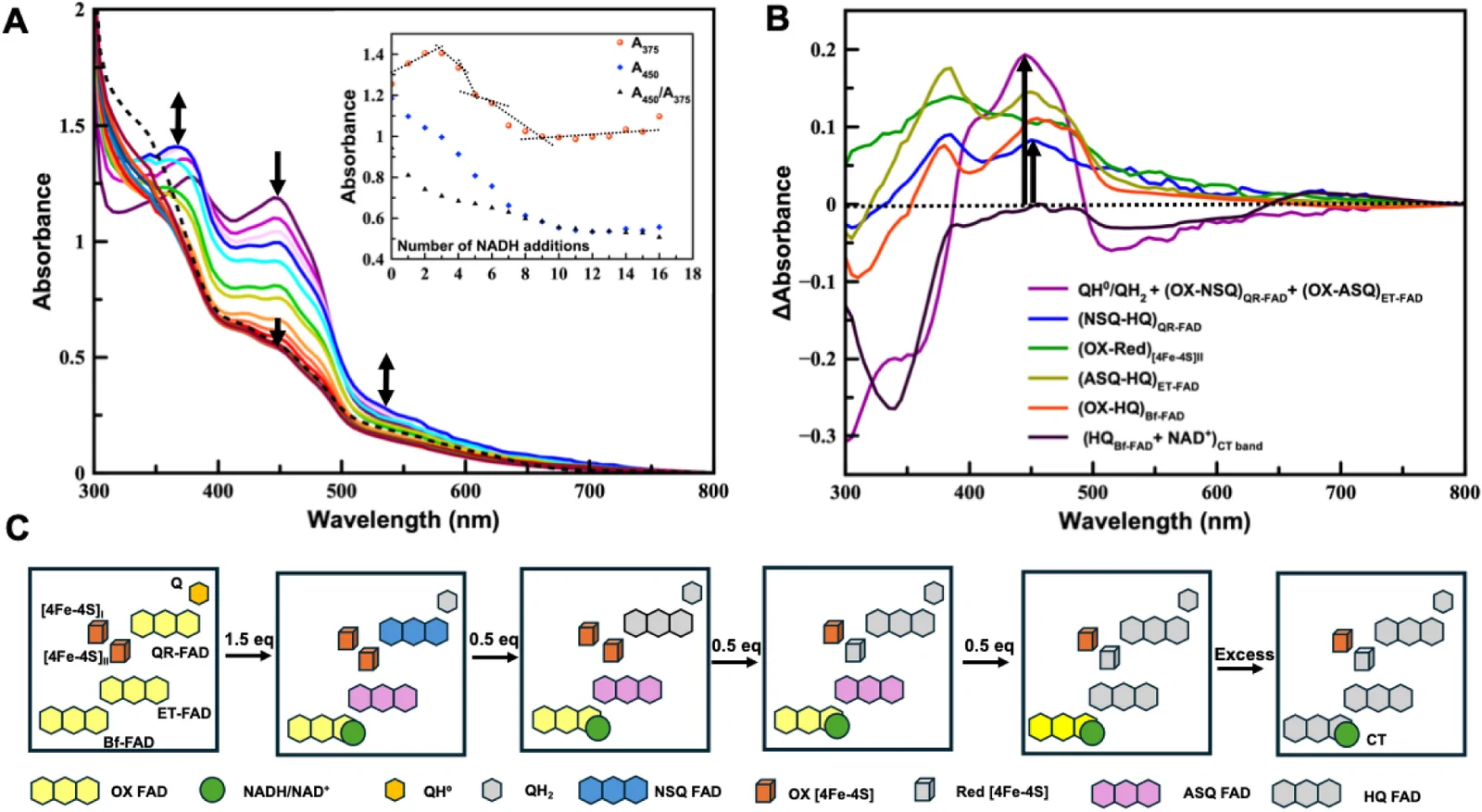
Reductive titration of air-oxidized *Av*FixABCX sample with NADH: A) UV–visible spectrum of air-oxidized *Av*FixABCX (13 μM) during sequential reduction with NADH, following the initial spectrum, each spectrum was collected after 0.5 eq of NADH addition, arrows left to right show formation and disappearance of SQ in band II at 375 nm, disappearance of band I intensity at 450 nm, slower reduction of Bf-FAD with an excessive amount of NADH, and formation and disappearance of NSQ at 500–600 nm; spectrum in the dashed black line shows formation of the CT band with excess NADH. Inset shows a plot of the absorbance at 375 nm, 450 nm, and the ratio of these absorbances against the number of NADH additions. B) Difference spectra corresponding to each redox event in *Av*FixABCX; the subsequent spectrum was subtracted from the preceding spectrum to create the difference spectrum, arrows show the absorbance ratio of two times of OX/SQ couple to the NSQ/HQ couple, and a decrease in absorbance above 500 nm in the difference spectrum due to NSQ and CT band formation is evident in the purple and black spectra, respectively. C) Cartoon diagram of the cofactor arrangement in the *Av*FixABCX (PDB ID 9ZQZ) showing the reduction scheme for all redox events in air-oxidized *Av*FixABCX in the presence of equimolar electrons or 0.5 eq of NADH.

Likewise, to reduce SQ to hydroquinone (HQ), 0.5 eq of NADH is required. Similarly, each [4Fe-4S] cluster requires 0.5 eq during 1e reduction. If Q is bound, it requires 1 eq of NADH to form SQ and then HQ. However, Bf-FAD is expected to undergo a 2e reduction step without accumulating an SQ intermediate as observed in other bifurcating Etfs.^11,21–24^ Overall, total reduction of bound cofactors requires 5 eq of NADH. During each reduction step, the absorbances at 375 and 450 nm, which correspond to the oxidized FAD, change significantly.^25^ Formation of an anionic or neutral SQ (ASQ or NSQ) state from the OX state exhibits an increase in the absorbance at 375 nm and a decrease in the absorbance at 450 nm concurrently. However, in NSQ, an additional increase in absorption is observed between 500 and 700 nm. When the ASQ or NSQ is further reduced to the HQ form, the absorption at both 375 and 450 nm (also 500–700 nm for NSQ) decreases. Similarly, oxidized [4Fe-4S]^3+/2+^ cluster absorbance decreases in the visible region from 300 to 500 nm when reduced to [4Fe-4S]^+^. A plot of the absorbance at 375 nm and 450 nm and the ratio of these absorbances against the number of NADH additions (inset in panel A) shows distinct behaviors in different phases of the reduction. To assign each reduction phase to the nature of the reaction governed by the corresponding redox-active cofactor, difference spectra of the spectra in Figure 3A were generated. The spectrum corresponding to maximum SQ formation (blue in panel A) was subtracted from the purple spectrum. Likewise, the subsequent spectra were subtracted from the preceding spectrum to create the difference spectrum shown in panel B. Each redox event has characteristic spectral features; based on these difference spectra, the reduction of the air-oxidized *Av*FixABCX sample reveals six redox events as shown in Figure 3B. These events are explained in the cartoon diagram shown in panel C for further clarification. During the first 1.5 eq of NADH (purple to blue in panel A and the corresponding purple difference spectrum in panel B), the OX form is converted to SQ, where three different redox events take place since 1.5 eq of NADH is being consumed, and the difference spectrum of these redox events corresponds to the conversion of the OX form of FAD to the SQ form. In addition, the intensity of the absorbance difference at ∼450 nm for the first redox event is double that of the second (shown in black arrows in panel B). The difference spectrum of the first redox event (purple spectrum shown in panel B) shows negative absorbance at 375 nm and 500–700 nm, due to the formation of NSQ. The subsequent reduction (blue to cyan in panel A) confirms the conversion of NSQ to HQ, as the absorbance at 375 nm and 500–700 nm decreases simultaneously, and the difference spectrum (blue spectrum in panel B) exhibits the characteristic features of the conversion of NSQ to the HQ form. Therefore, one of the redox events in the first difference spectrum is the conversion of the OX form of QR-FAD to the NSQ form. Since the spectral features indicate that this redox event is the conversion of the OX form to SQ, the second 0.5 eq of NADH is required to reduce the OX form of the higher-potential ET-FAD to the ASQ form; Bf-FAD is not expected to accumulate SQ, and the reduction potential of Bf-FAD is lower than that of ET-FAD. The third 0.5 eq of NADH results in the conversion of the higher-potential SQ state (QH°) of bound Q to the HQ form (QH_2_), which does not show a significant spectral change in the visible region from 300 to 500 nm. As the EPR spectrum of the air-oxidized *Av*FixABCX sample has an isotropic radical signal (Figure 5A), Q is present in the QH° form and is being reduced to QH_2_ during the first redox event. These three redox events could be taking place consecutively, as the reduction potentials of ET-FAD_OX/ASQ_ and Q_QH°/QH2_ are in the same range, as described below, and QR-FAD_OX/NSQ_ is directing the electron between these two redox couples and has the next higher potential. Therefore, these redox events are indistinguishable in the first redox event. The second redox event is due to the conversion of the NSQ form of QR-FAD to the HQ form as the absorbance at 375 nm and 500–700 nm decrease simultaneously (blue to cyan in panel A and dark blue difference spectrum in panel B). The difference spectrum of the third redox event (cyan to green in panel A and green difference spectrum in panel B) differs in spectral features from FAD difference spectra, suggesting the reduction of a higher-potential [4Fe-4S] cluster. This step consumes only 0.5 eq of NADH because only one cluster is reduced. The fourth reduction event shows the conversion of the ASQ form of ET-FAD to the HQ form (the yellow-green difference spectrum in panel B). The last step is due to the reduction of the OX form of Bf-FAD, which is converted to the HQ form. The characteristic red difference spectrum in panel B shows that Bf-FAD undergoes a one-step 2e reduction without stabilizing SQ, unlike the ET- and QR-FAD. This is supported by our TAS data explained below and agrees with previously published results in other bifurcating EtfABs.^5,11,21,22^ The Bf-FAD reduction step requires an excess of NADH to drive the reduction to completion and leads to the formation of a charge-transfer (CT) species (black difference spectrum in panel B), as previously reported in bifurcating Etfs from *A. fermentans* EtfAB, *Pyrobaculum aerophilum* *(Pae)*EtfAB/ABCX, and *Tma*EtfABCX complexes when NAD­(H) is in excess.^11,16,26,27^ The absorption difference for FAD reduction from the OX form to the HQ form is 0.2 absorbance units in both QR-FAD and ET-FAD. An absorbance difference of 0.1 instead of 0.2 in the red difference spectrum indicates that only half the population of Bf-FAD is being reduced; further NADH additions increase the intensity at 340 nm and between 500 and 650 nm without causing a significant change in the 350–500 nm visible region as shown in the black difference spectrum in panel B. Therefore, we propose that some populations of the negatively charged HQ form of Bf-FAD could be transferring electrons to the empty orbitals of an excess amount of NAD^+^ in the mixture and forming the HQ_Bf‑FAD_-NAD^+^ CT band. In addition, the higher absorption of NADH at 340 nm indicates the presence of excess unoxidized NADH, confirming that the CT band is due to the HQ form of Bf-FAD and NAD^+^. Since the CT complex stabilizes the interaction between HQ_Bf‑FAD_ and NAD^+^, the reverse electron transfer due to the confurcation of electrons from already populated QH_2_ and Fld_HQ_ may be prevented, or this could be a characteristic mechanism of Bf systems that temporarily holds the electron during confurcation to prevent the accumulation of the higher energy OX/SQ state of Bf-FAD. It was assumed that ET-FAD exists in the ASQ state in a reducing cellular environment due to its higher reduction potential.^6,11,22,28^ Interestingly, as-purified *Av*FixABCX shows the presence of partially reduced ET-FAD, QR-FAD (in ASQ/NSQ form), and [4Fe-4S] cluster (Supporting Information Figure S6G) and requires only around 2 eq of NADH for full reduction. In the air-oxidized sample, ET-FAD exists in the OX form and is reduced to the ASQ form at the first step of electron transfer; this stable ASQ state allows single-electron transfer from Bf-FAD to Q. *Av*FixABCX can be reduced by NADH in the absence of Fld and the formation of single-electron-reduced SQ states in ET- and QR-FAD confirms that Bf-FAD transfers a single electron via the ASQ form of ET-FAD. Furthermore, the formation of ASQ in the first redox event ensures single-electron transfer from Bf-FAD to Q. Overall, *Av*FixABCX contains bound Q in a partially reduced QH° state, and the reductive titration confirms that only one of the [4Fe-4S] clusters is being reduced, which is presumed to reside in the higher-potential electron-transfer pathway as it is being reduced before the Bf-FAD.

### Determination of the Reduction Midpoint Potential of FADs and [4Fe-4S] Clusters in *Av*FixABCX

To provide more insight into the energetics of the electron-transfer pathway in *Av*FixABCX, the reduction midpoint potential values of FADs and [4Fe-4S] clusters were determined using protein thin-film square-wave voltammetry (PTF-SWV). To simplify the system and measure the reduction midpoint potentials of FADs and [4Fe-4S] clusters separately, PTF-SWV was carried out on *Av*FixAB, *Av*FixCX, and *Av*FixX proteins individually. SWV is more sensitive than cyclic voltammetry because it enhances nonfaradaic currents.^29^ We expect two reduction potential values, *E*_*OX/SQ*_ and *E*_*SQ/HQ*_, for both ET-FAD and QR-FAD, but for Bf-FAD only one reduction potential can be measured *E*_OX/HQ_ due to the SQ state has a short lifetime. With the two [4Fe-4S] clusters and Q (in Q, the reduction potential of QH°/QH_2_ (190 mV) is greater than that of Q/QH° (−230 mV) therefore it is unlikely to observe the Q/QH° formation),^30^ in total eight reduction potential values must be measured in *Av*FixABCX. However, only three distinct potential values, −600 mV, + 50 mV, and +220 mV (*E*, SHE) were obtained at pH 8.5 (yellow voltammogram in Figure 4A) in both reducing and oxidizing conditions for *Av*FixABCX. To observe the potential values of [4Fe-4S] clusters, PTF-SWV was performed on nonreconstituted and reconstituted *Av*FixX with and without the MBP tag. Two distinct potential values, −30 mV and −500 mV (*E*, pH 8.0, SHE), were obtained in all the above conditions. The intensity of the current in SWV increased in the reconstituted sample, presumably due to the presence of fully occupied FeS clusters (Figure 4C). Since the observation of two very disparate reduction potentials was unanticipated, we examined the homologue FixX from another microbial source, *Roseoflexis castanholzii* (*Rcas*). *Rcas*FixX also indicates two distinct reduction potentials, −167 and −650 mV, for the [4Fe-4S] clusters. Based on these results, −30 mV was assigned to the [4Fe-4S]_II_ cluster in the higher-potential pathway, and −500 mV was assigned to the [4Fe-4S]_I_ cluster.

**Figure 4:**
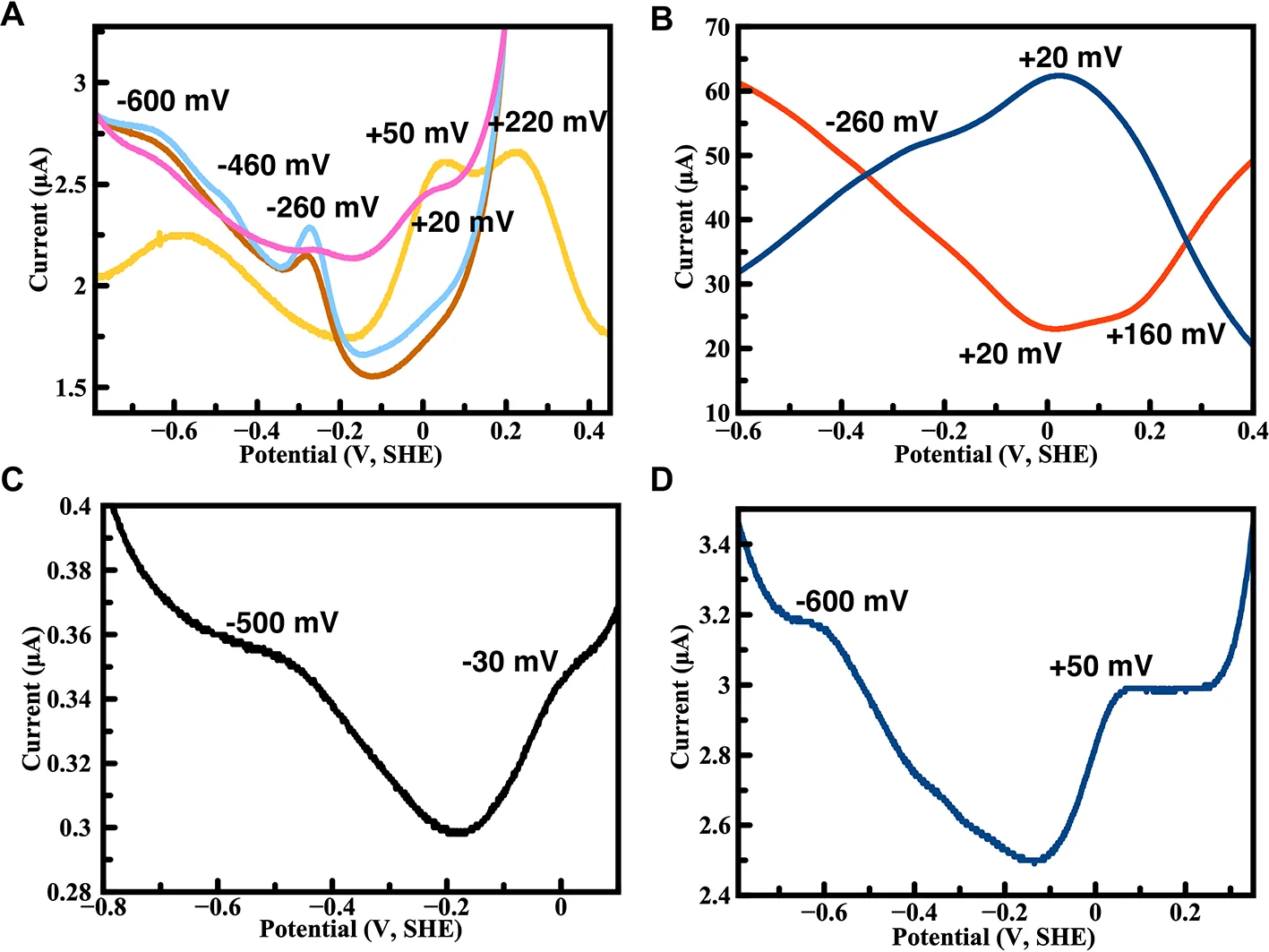
Protein thin-film square-wave voltammograms of A) *Av*FixABCX voltammograms collected in the reducing direction; the yellow voltammogram is from as-purified *Av*FixABCX, the brown voltammogram is of *Av*FixABCX:Fld_NSQ_ at a 1:2 ratio, the light blue voltammogram is of *Av*FixABCX:Fld_NSQ_:NADH at a 1:2:2 ratio, and the pink voltammogram is of *Av*FixABCX:NADH at a 1:2 ratio; B) *Av*FixAB voltammograms collected in both the oxidizing (red) and reducing (blue) diections; C) *Av*FixX voltammogram in the reducing direction; and D) *Av*FixCX voltammogram in the reducing direction. Reduction midpoint potential values, *E,* +220 mV, +50 mV, −30 mV −500/–600 mV, +160 mV, +20 mV, and −260 mV (E SHE) are the expected reduction potentials of QH°/QH_2_, QR-FAD_OX/NSQ_, two [4Fe-4S] clusters, *E*_*OX/SQ*_, and *E*_*SQ/HQ*_ of ET-FAD, and the single reduction potential *E*_OX/HQ_ of Bf-FAD.

Based on the lower-potential values obtained for [4Fe-4S]_I_ in *Av*FixX, the −600 mV value observed in *Av*FixABCX was assigned to the off-pathway [4Fe-4S]_I_ cluster. Two higher potential values observed in *Av*FixABCX, +50 mV and +220 mV, were assigned to the redox couples OX/NSQ of QR-FAD and QH°/QH_2_ of Q, respectively. The number of electrons transferred in an immobilized redox couple can be determined using the peak width at half height of the corresponding peak. At 25 °C, the peak width at half height is 90/n mV, where n is the number of electrons transferred in a simultaneous or cooperative process.^31,32^ The peak widths at half height for the peaks at +50 mV and +220 mV were 90 mV and 91 mV, respectively, suggesting that these peaks are due to a single-electron transfer. Membrane-bound Q reduction potentials can vary from +94 mV to +230 mV.^33–35^ Reduction potential measurements in Coenzyme Q show that the reduction potential of QH°/QH_2_ (190 mV) is greater than that of Q/QH° (−230 mV);^30^ therefore, +220 mV is due to the QH°/QH_2_ couple. As we observed above in the redox titration, the +220 mV QH°/QH_2_ couple and the OX/NSQ couple of QR-FAD get reduced in the first redox event, and the NSQ/HQ couple of QR-FAD reduced later. Therefore, the +50 mV potential observed in this study was assigned to the redox couple OX/NSQ of QR-FAD. Additionally, SWV of FixCX purified independently of the complex is in the oxidized form and exhibits a maximum current only at +50 mV and −600 mV (*E*, SHE) (Figure 4D), suggesting that +50 mV is due to the OX/NSQ couple and the absence of the potential value at +220 mV could be due to the absence of Q in FixCX. The analysis, however, confirms that the reduction potential value of +50 mV is due to QR-FAD, while −600 mV is for the [4Fe-4S]_I_ cluster.

SWV of *Av*FixABCX did not exhibit individual peak currents for the higher-potential [4Fe-4S]_II_ cluster, QR-FAD_NSQ/HQ_, ET-FAD, or Bf-FAD. However, the peak below −200 mV is broader, even though the maximum peak current is observed at −600 mV. To determine the potential values of ET-FAD and Bf-FAD, the SWV of the reconstituted *Av*FixAB was assessed. In the oxidizing direction, two peak currents were observed at +20 mV and +160 mV, whereas in the reducing direction, two peak currents were observed at +20 mV and −260 mV (Figure 4B). Three potential values are expected in FixAB due to the OX/SQ and SQ/HQ couples of ET-FAD and the OX/HQ couple of Bf-FAD. This is based on the UV–vis redox titration explained above,and has been previously reported in other Etf/FixAB systems as well.^11,16,21,23^ During the EB reaction, Bf-FAD is known to destabilize the SQ state by having a crossed potential. In addition, Bf-FAD has a lower reduction potential than that of ET-FAD. Hence, −260 mV is assigned to the OX/HQ couple of Bf-FAD. ET-FAD has a standard uncrossed potential with a stable SQ state; therefore, +160 mV was assigned to the OX/SQ redox state of ET-FAD, while +20 mV was assigned to the SQ/HQ state.

Reduction potentials of ET-FAD and Bf-FAD have been previously determined experimentally in *Megasphaera elsdenii* EtfAB, *A. fermentans* EtfAB, *Pae*EtfAB, *Rhodopseudomonas palustris* FixAB, and *Sulfolobus acidocaldarius* EtfAB; a 2e^–^ reduction potential of −240 to −280 mV for Bf-FAD, +81 to −60 mV for the OX/SQ couple of ET-FAD, while for SQ/HQ couple it is from −36 to −136 mV.^11,15,16,21,23,28^ A difference of more than 60 mV between the OX/SQ and SQ/HQ couples indicates that a stable SQ state forms in ET-FAD across all of these bifurcating systems.^25^ Reduction midpoint potentials measured in *Av*FixAB fall within this range of *E* values, as reported by others.^11,15,16,21,23^

To assess the Bf-FAD reduction potential in *Av*FixABCX, *Av*Fld_NSQ_ was mixed with *Av*FixABCX at a 1:2 ratio and treated with NADH. The light blue voltammogram in Figure 4A shows peak currents at +20 mV, −260 mV, −460 mV, and −600 mV. The reduction potentials of the NSQ/HQ state of *Av*Fld were reported as −483 mV and −500 mV at pH 7.5.^36,37^ In other long-chain Fld works, a range from −400 mV to −465 mV^38,39^ was reported. Therefore, −460 mV was assigned to the *E*_NSQ/HQ_ state of *Av*Fld. In line with what is observed for *Av*FixAB, −260 mV is assigned to the OX/HQ state of Bf-FAD. A narrow line shape observed for the peak at −260 mV and the peak width at half height of 53 mV (as explained above, peak width at half height equals 90/n mV, and for a 2e transfer it is 45 mV)^23,32^ confirms that it is a simultaneous 2e transfer. The peaks at +20 mV as observed in the FixAB voltammogram and −600 mV as in *Av*FixX correspond to the SQ/HQ state of ET-FAD and the lower-potential [4Fe-4S]_I_, respectively. *Av*FixX has 2­[4Fe-4S] clusters and contains an Fd-like sequence and fold. Most 2­[4Fe-4S] cluster-containing Fds have two clusters with similar reduction midpoint potentials varying from −380 mV to −725 mV in different organisms.^40^ The low-potential cluster prevents electron flow from [4Fe-4S]_II_ to [4Fe-4S]_I_ but ensures electron flow from ET-FAD to QR-FAD only via [4Fe-4S]_II_, as shown in Figure 2C above. It is not known if the off-pathway low-potential [4Fe-4S]_I_ cluster is functionally significant.

### EPR Redox Titration

To confirm the assignments of the reduction potentials of the *Av*FixX [4Fe-4S] clusters, we carried out a potentiometric titration of air-oxidized *Av*FixABCX by monitoring with EPR spectroscopy (Figure 5A). Oxidized *Av*FixABCX shows a strong isotropic *g*_*iso*_ = 2.003 signal before the addition of sodium dithionite (NaDT), which is attributed to the presence of QH° as confirmed by redox titration and SWV. During the NaDT titration (Figure 5A), the intensity of the radical signal increases as the potential decreases to −157 mV, then decreases as the potential further decreases. The initial increase is due to the increasing concentration of the paramagnetic SQ state of QR-FAD and ET-FAD. Further addition of NaDT reduces the SQ state to the diamagnetic HQ state. During the NaDT titration, two signals associated with a reduced [4Fe-4S]^+^ cluster appear in Figure 5A, denoted as signals 1 and 2 in the expanded spectra shown in Figure 5B. The *g*_1_ features of these two signals are quite well resolved, but the others largely overlap. However, careful examination of spectra taken in the 3.8–15 K temperature range allowed decomposition of the signal into component rhombic spectra (Figure 5B and C): signal 1, *g*_1_ = 2.075, *g*_2_ = 1.941, *g*_3_ = 1.891; signal 2, *g*_1_ = 2.040, *g*_2_ = 1.941, *g*_3_ = 1.879. The intensity of these signals increases during the course of NaDT addition (Figure 5A), and a plot of the amplitudes of the well-resolved cluster *g*_1_ features against the measured potential (Figure 5D) indicates essentially the same reduction potentials: −139 mV for signal 1 and −131 mV for signal 2. Quantitation of the rhombic EPR signal vs CuSO_4_ standard shows that the combined intensity of the two signals, when maximized, accounts for ∼50% of [4Fe-4S] clusters in the total protein. Given this and the equivalent reduction potentials, we assign these rhombic signals to two conformers of a single S = 1/2 [4Fe-4S] cluster, the higher-potential [4Fe-4S]_II_ cluster.

**Figure 5:**
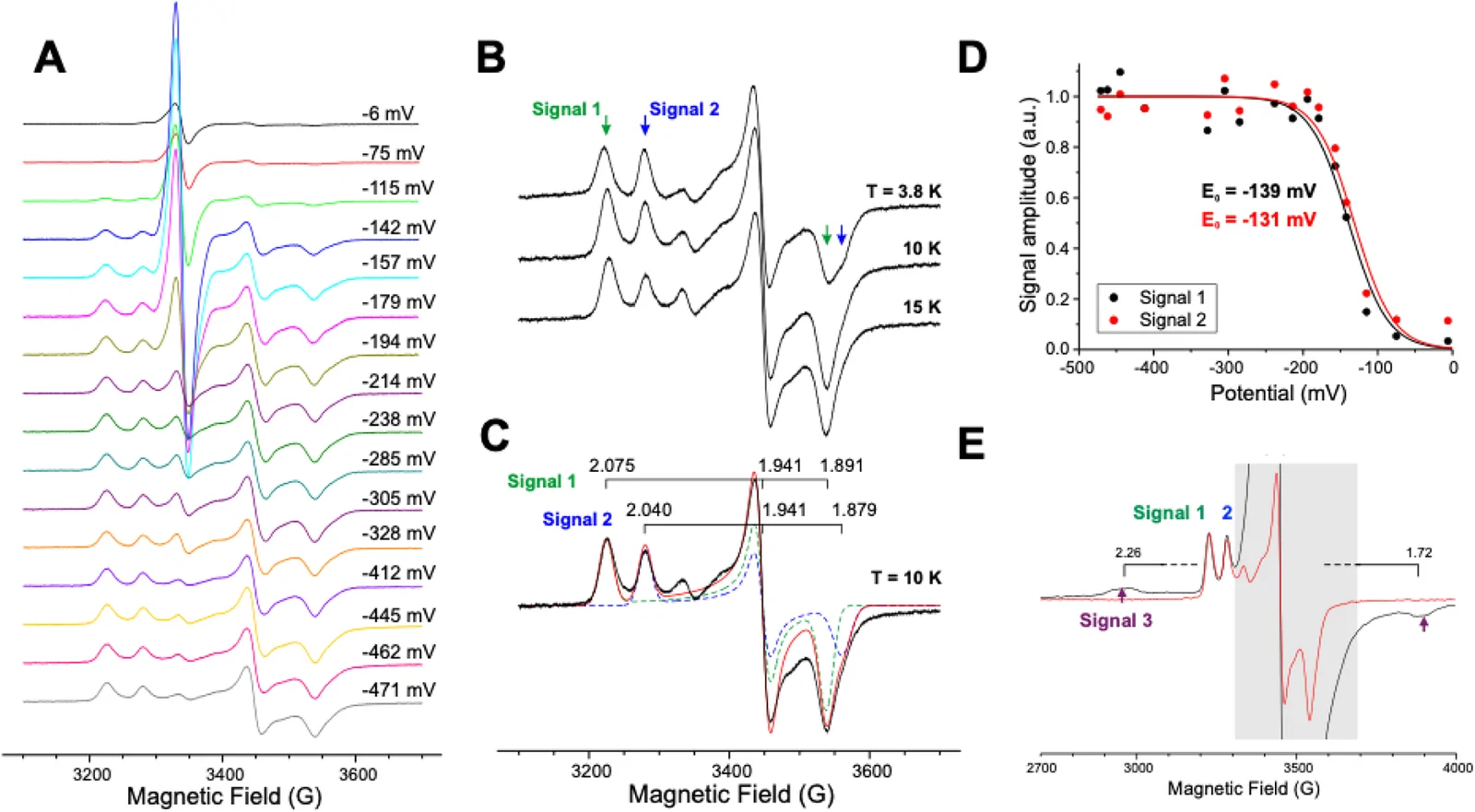
Electron paramagnetic resonance (EPR) spectroscopic analysis of air-oxidized *Av*FixABCX: A) EPR spectrum of *Av*FixABCX (72.9 μM) samples collected at 10 K, 9.36 GHz microwave frequency, 0.24 mW power,with 13 G modulation amplitude; samples were collected after each addition of sodium dithionite (NaDT), and the potential values of the reaction mixture were measured (−6 mV through −471 mV) using an ORP electrode with an Ag/AgCl reference electrode. B) Temperature dependence of the EPR for the sample corresponding to the −471 mV reduction potential: as the temperature decreases from 15 K to 3.8 K, signal 1 and signal 2 saturate differently. C) Simulation of the rhombic signals of signal 1 and signal 2 collected at 10 K. D) Plot of the amplitudes of signals 1 and 2 from spectra shown in (A) against the measured potential values: the plot shows reduction midpoint potentials of −139 mV and −131 mV for [4Fe-4S] cluster. E) Further reduction of *Av*FixABCX with excess Ti­(III) citrate shows the appearance of another rhombic signal 3 along with signals 1 and 2, indicating the presence of a lower-potential [4Fe-4S] cluster.

The spin quantum number S = 1/2 of the reduced [4Fe-4S]^+^ cluster gives rise to a rhombic EPR signal.^41^ For a [4Fe-4S]^+^ cluster, six valence isomers can exist due to electron delocalization among the constituent Fe^3+^ and Fe^2+^ within the clusters.^42^ This can give pairs of Fe^2.5+^ ions coupled to two Fe^2+^ ions^42–44^ in which the extra electron resides in a pair of ions.^42,44^ This charge distribution can cause distinctive *g* values in the EPR signal, and the charge distributions in the cubane can be influenced by side-chain residues. Thus, the two rhombic signals of [4Fe-4S]_II_ could arise from valence isomers in the [4Fe-4S]_II_^+^ state, and this charge distribution in the cubane could be influenced by the presence of an H-bonding Tyr47 residue near Cys68 (see Figure S7 in the Supporting Information). When Tyr47 forms an H-bond to Cys68 coordinated to the [4Fe-4S]_II_ cluster, the electronegativity of [4Fe-4S]_II_ should be increased, and the interaction between Cys68 and the Fe ion is weakened. This interaction could contribute to the more positive reduction potential observed for [4Fe-4S]_II_. Similar Tyr H-bonding interactions exist in higher-potential [4Fe-4S] clusters in porcine (*E* = +47 mV) and *Rhodobacter sphaeroides* (*E* = +37 mV) Etf-QO.^45,46^ Mutation of Y501 in *R. sphaeroides* Etf-QO to F resulted in a decrease in the reduction potential of the [4Fe-4S] cluster from +37 mV to −64 mV. The presence of charge redistribution arising from interactions with side-chain amino acid residues and valence/spin isomers in [4Fe-4S] clusters has been reported in many other systems. An MD simulation study on the single [4Fe-4S]-containing apo form of the [FeFe]-hydrogenase from *Chlamydomonas reinhardii* (HydA1) shows that charge distribution in [4Fe-4S]^+^ is influenced by Arg and Lys residues.^43^ In addition, different EPR signals were observed in higher-potential [4Fe-4S]^3+^ clusters of HiPIPs, due to the presence of more than one valence isomer.^44^ The reduced [4Fe-4S]^+^ cluster of the corrinoid protein from *Clostridium thermoaceticum* exists as two isomers.^47^ EPR spectra of adenosine 5′-phosphate reductase from *Mycobacterium tuberculosis* also exhibit two overlapping S = 1/2 species.^48^ Overall, the presence of valence isomers in [4Fe-4S] clusters is a common observation, and in *Av*FixABCX, H-bonding interactions between Tyr47 and Cys68 could play an important role in the more positive observed reduction potential of [4Fe-4S]_II_, influence the charge distribution, and underlie the occurrence of the observed valence isomers.

During the titration with NaDT, the potential decreased from −6 mV to −471 mV. No change in potential was observed after −471 mV despite further NaDT addition. This is because NaDT cannot reduce further ∼−476 mV at pH 8.^49,50^ To observe the EPR signal of the reduced lower-potential [4Fe-4S]_I_ cluster, we treated *Av*FixABCX with Ti­(III) citrate. The Ti­(III) citrate reduction potential can be as low as −650 mV at pH 8.5, and it further decreases with increasing concentration of Ti­(III) citrate.^51,52^ The reduction with Ti­(III) citrate of course generates signals 1 and 2 from the higher-potential [4Fe-4S]_II_ cluster (Figure 5E), but it also generates another rhombic cluster signal with broad features at *g*_1_ = 2.26 and *g*_3_ = 1.72 from the lower-potential [4Fe-4S]_I_ cluster; the strong signal from the high concentration of Ti­(III) citrate obscures the region where *g*_2_ feature of this [4Fe-4S]_I_ cluster is expected (gray area in Figure 5E).

From the EPR analysis, in conjunction with our potential measurements and cryo-EM structure, we can now conclude that the [4Fe-4S]_II_ cluster has a higher potential and is on the pathway of electron bifurcation in *Av*FixABCX. The role of the lower-potential [4Fe-4S]_I_ cluster is unknown, but it is unlikely to participate in redox catalysis or serve as an alternative conduit for electron donor–acceptor interactions because of its low potential (≤600 mV). The unusually low potential could be an evolutionary adaptation to prevent electron-transfer short-circuiting, or the low-potential cluster may influence the properties of the on-pathway [4Fe-4S]_II_. We further note that there is almost a −100 mV shift in reduction potentials (−30 mV to −131 mV and −500 mV to −600 mV) of the [4Fe-4S] clusters in *Av*FixABCX compared to *Av*FixX, which is likely a consequence of differences in the overall protein environment in *Av*FixX alone versus in the *Av*FixABCX complex.

### Ultrafast Transient Absorption to Probe Flavin Species

To probe the transient flavin species formed at the FAD cofactors in FixABCX, we performed ultrafast TA spectroscopy in the presence and absence of Fld_NSQ_ (Figure S8 and Table S6). Fld_NSQ_ alone was measured as a control to verify that no new signals were introduced by photoexcitation of Fld. Under both conditions, a short-lived NSQ signal was observed and assigned to the QR-FAD. It is rapidly formed by electron transfer from [4Fe-4S]_II_ and it decays due to back-electron transfer with the FeS cluster with a rate constant that, within error, is independent of the presence of Fld (t1 = 210 ± 19 ps/180 ± 40 ps without and with Fld present). A short-lived ASQ signal was also observed displaying biphasic decay (t2 = 260 ± 100 ps/140 ± 83 ps, t3 = >5000 ps without and with Fld present), consistent with previous studies on similar systems.^11,17^ However, the addition of Fld did not impact the ASQ (or NSQ) rates; therefore we tentatively assign the ASQ signals to the ET-FAD. In these specific experiments, electron transfer between Fld_NSQ_ and Bf-FAD was not observed, consistent with the reduction potentials of these species. Furthermore, the presence of Fld_NSQ_ does not appear to alter the kinetics along the high-potential pathway

### Energy Landscape in *Av*FixABCX

Based on the measured reduction potential values using PTF-SWV and EPR redox titration, an energy landscape for the EB mechanism in *Av*FixABCX can be formulated. As Nitschke and Russell reported, the stability constant *K*_*S*_ of the SQ state in Bf-FAD should be as low as 10^–14^ for an energetically favorable electron bifurcation to take place.^53^ To observe a short-lived SQ state, *E*_*SQ/HQ*_ should be much higher than the *E*_*OX/SQ*_ of Bf-FAD.^53,54^ The difference between these two *E* values in the Nernst equation is equal to 59 log *K*_*S*_, where *K_S_* represents [SQ]^2^/[OX] × [HQ]. Hence, Δ*E* = 59 log 10^–14^ equals −826 mV, and the addition of *E*_*SQ/HQ*_ and *E*_*OX/SQ*_ equals (−260 × 2) mV. Using Δ*E* and the addition of *E*_*SQ/HQ*_ and *E*_*OX/SQ*_, *E*_*SQ/HQ*_ was calculated as +153 mV and *E*_*OX/SQ*_ as −673 mV. In NfnSL, *E*_*OX/SQ*_ of −911 mV was experimentally determined from measuring the lifetime of electron transfer between the bifurcating flavin and the nearest [4Fe-4S]. Also, in other bifurcating EtfABs, *E*_*SQ/HQ*_ and *E*_*OX/SQ*_ were calculated using an assumed SQ stability constant of 10^–12^–10^–15^.^16,55^ Consequently, the calculated values of *E*_*SQ/HQ*_ and *E*_*OX/SQ*_ are in the same range as previously reported in bifurcating Etfs.

During electron bifurcation, NADH (*E* = −320 mV) transfers 2e to Bf-FAD (*E*_*OX/HQ*_ = −260 mV). Then the SQ/HQ state (*E*_*SQ/HQ*_ = +153 mV) of Bf-FAD transfers 1e to the ASQ state ET-FAD (*E*_*SQ/HQ*_ = +20 mV). According to the energy landscape, electron transfer from the SQ/HQ state (*E*_*SQ/HQ*_ = +153 mV) of Bf-FAD to either *E*_*OX/SQ*_ = +160 mV or *E*_*SQ/HQ*_ = +20 mV is favorable; however, the ASQ state is assumed to be stabilized in a reducing cellular environment, also the ASQ state accumulates in the as-purified *Av*FixABCX sample (as shown above), this is the energetically favorable electron transfer compared to the electron transfer to Fld (*E*_*NSQ/HQ*_ = −460 mV). This downhill electron transfer drives the electron transfer from the OX/SQ state of Bf-FAD (*E*_*OX/SQ*_ = −673 mV) to bound Fld_NSQ_ (*E*_*NSQ/HQ*_ = −460 mV). In the higher-potential pathway, the fully reduced HQ form of ET-FAD transfers 1e to the [4Fe-4S]_II_ cluster (*E* = −131 mV) and returns to ASQ; then the reduced [4Fe-4S]_II_ cluster transfers 1e to the OX form of QR-FAD (*E*_*OX/NSQ*_ = +50 mV). Based on the redox titration in Figure 3, the OX/NSQ redox couple reduces first and has a higher reduction potential than NSQ/HQ; therefore, the OX form of QR-FAD is converted to the stable NSQ state. Then, the second round of electron transfer from NADH converts the NSQ state of QR-FAD to HQ; the NSQ/HQ couple can reduce Q to QH° since both redox couples have comparatively similar redox potentials (see above), and then the OX/NSQ couple (+50 mV) can transfer the electron to QH° (*E* = +220 mV) and turn to the OX form. Likewise, two rounds of NADH oxidation take place at Bf-FAD to reduce 1 eq of Q to QH_2_ and 2 eq of Fld_NSQ_ or Fd (reaction stoichiometry was obtained in the section below).

### Bifurcation Reaction Is Slower Than Confurcation

Steady-state kinetic experiments were carried out to determine the rate of electron transfer for the bifurcation, confurcation, and each individual half-reaction in bifurcation and confurcation. The nonequilibrium reduction midpoint potentials of NADH (−480 mV), NAD^+^ (−380 mV), Q (+260 mV), QH_2_ (−460 mV), Fld_HQ_ (−580 mV), and Fld_NSQ_ (−340 mV) were calculated using the standard reduction potentials of the NADH/NAD+ (−320 mV) and Q/QH_2_ (+200 mV) couples and the measured equilibrium reduction potential of Fld_NSQ_/Fld_HQ_ (−460 mV) couple using the Nernst equation^56–58^ (Supporting Information Table S4). The specific activities of all the reactions, turnover number, and the reaction ΔG were tabulated in Supporting Information Table S5. Air-oxidized *Av*FixABCX can be reduced by NADH, and each redox event requires 0.5 eq of NADH, indicating that all the electrons are transferred to the higher-potential pathway from Bf-FAD to Q (as shown above in Figure 3A and 3B), and this electron transfer happens at a measurable rate. However, when the reduction of an excess amount of Q with NADH in the presence of a catalytic amount of *Av*FixABCX was monitored, the reaction completed in less than 2.0 ms, which is the dead time of the stopped-flow instrument. Changes in absorbance due to NADH oxidation at 340 nm and the intensity at 280 nm for Q reduction yield a 1:1 stoichiometric ratio between Q and NADH (Supporting Information, Figure S9A). This confirms an energetically favorable downhill reaction (ΔG = −42.5 kJ/mol) that directs 2e from NADH to Q alone and suggests that *Av*FixABCX can short-circuit the electron transfer from NADH to Q in the absence of Fld_NSQ_. In contrast, the uphill, NADH-mediated Fld_NSQ_ reduction is extremely slow, as expected, with a specific activity of 0.045 nmol/mg/min. Electron transfer from QH_2_ to NAD^+^ in the absence of Fld_HQ_ from *Av*FixABCX is much slower, with an activity of 2.5 nmol/mg/mol, compared to the forward half-reaction from NADH to Q. This is because the electron flow from −460 mV QH_2_ to −380 mV NAD^+^ is energetically unfavorable (ΔG = −15.4 kJ/mol) compared to the forward half-reaction (ΔG = −42.5 kJ/mol). In contrast, the activity of *Av*FixABCX for electron transfer from Fld_HQ_ to NAD^+^ is 7.7 nmol/mg/min, almost three times higher than the QH_2_-to-NAD^+^ electron transfer, because electron flow from −580 mV Fld_HQ_ to −380 mV NAD^+^ is energetically favorable (ΔG = −38.6 kJ/mol).

Differences in the rate of electron transfer in the individual half-reactions are reflected in the overall bifurcation and confurcation activity. Bifurcation reaction activity of *Av*FixABCX in the presence of Q, Fld_NSQ_, and NADH is 0.87 nmol/mg/min, while the confurcation activity in the presence of QH_2_, Fld_HQ_, and NAD^+^ is 7.4 nmol/mg/min. Confurcation activity was almost 10 times higher than that of bifurcation, even though the overall ΔG is +5 kJ/mol higher than that for the bifurcation reaction. This is due to a higher activation barrier for the bifurcation reaction. In contrast, the confurcation reaction takes place at a faster rate to establish the equilibrium by consuming the reactant and forming the product of interest. The higher activation barrier for the electron bifurcation mechanism could be due to its dependence on conformational changes, which can occur more slowly. The electron transfer from Fld_HQ_ to NAD^+^ in the absence of QH_2_ has the same rate as confurcation (see above); however, electron transfer from QH_2_ to NAD^+^ is 3 times slower. This means that the rate of confurcation is controlled by Fld_HQ_ binding rather than electron transfer from QH_2._ Similarly, in the presence of Fld_NSQ_, the bifurcation reaction takes place at a measurable rate, but much slower than the downhill half-reaction and relatively faster than the uphill half-reaction. Collectively, the bifurcation and confurcation rates are affected by the presence of Fld_NSQ_ or Fld_HQ_, even though the electron transfer to the uphill, low-potential branch is driven by electron transfer to the downhill, higher-potential branch.

To determine the stoichiometry between the electron donor and acceptor, we used spectral deconvolution of steady-state kinetic data (as explained in the Supporting Information, Figures S9 and S10). In the forward and reverse half-reactions of the higher-potential branch (downhill) involving NADH and Q, the stoichiometry was 1:1. Similarly, the reverse half-reaction in the uphill pathway from Fld_HQ_ to NAD^+^ was 1:1. However, the reaction stoichiometry among NAD^+^:Fld_HQ_:QH_2_ was 2:2:1 for the confurcation. As shown in Supporting Information Figures S9 and S10, the spectral change was much smaller in the bifurcation reaction than in confurcation because the reaction rate was much slower; however, spectral deconvolution showed the reduction of Q at 280 nm and Fld_NSQ_ at 580 nm by NADH. The stoichiometry of 2:2:1 is obtained for NADH:Fld_NSQ_:Q in the bifurcation reaction. In the absence of excess Fld_NSQ_, *Av*FixABCX directs the electrons from NADH via Bf-FAD to bound Q as explained in Figure 3. Similarly, as the steady-state reaction stoichiometry data show, in the presence of excess Q, electrons from NADH are directed rapidly to Q via the higher-potential pathway, whereas in the presence of excess Fld_NSQ_, a much slower electron transfer occurs directly from Bf-FAD to Fld_NSQ_. However, when NADH, Q, and Fld_NSQ_ are present, *Av*FixABCX directs the electrons from NADH to both Q and Fld_NSQ_, confirming a sequential two-electron transfer, leading to electron bifurcation.

### Electron Transfer in EB Is a Conformationally Gated Mechanism

According to the cryo-EM structure, the distance between Bf-FAD and ET-FAD is 25 Å, which is too long for a reasonable rate of electron transfer in an enzyme, and we anticipate that a conformational change occurs in domain II to bring ET-FAD and Bf-FAD closer to one another.^59^ Domain II of canonical Etfs related to FixABCX has been implicated in undergoing conformational change during electron transfer.^60–65^ In the structural work describing *Tma* Fix/EtfABCX, it was noted that two distinct conformational states were reported in higher-resolution structures of homologous bifurcating systems. In *A. fermentans* EtfAB, ET- and Bf-FADs are separated by 18 Å in the closed conformer.^5^ In *Clostridium difficile* EtfAB-Bcd, *Acetobacterium woodii* caffeyl-CoA reductase (CarCDE), and EtfAB-lactate dehydrogenase, ET-FAD and Bf-FAD are ∼37 Å apart in the open conformer.^55,66,67^ Superimposition of these observed conformations indicates that domain II can rotate 75–80°.^55,67^ In the *Av* and *Tma* Fix/EtfABCX structures, ET-FAD and Bf-FAD are observed on this same domain movement trajectory, with an intermediate distance of 25 Å^6^. A conformational change in domain II along this trajectory is expected to modulate the distance between *Av*FixABCX ET-FAD and Bf-FAD as proposed for the *Tma* Fix/EtfABCX structure, bringing them into closer proximity and thereby controlling the rate of electron transfer.^6^

The experimentally determined reduction midpoint potentials of the redox cofactors and the observed distances in the structures were used to estimate the electron transfer rates using the Moser-Dutton equation^59,68^ (tabulated in Supporting Information Table S3 and Figure 2C). As shown in Figure 6, Bf-FAD exhibits inverted potentials, where the reduction potential of Bf-FAD_OX/SQ_ is more negative than that of ET-FAD_SQ/HQ_. ET-FAD is observed in the SQ state in as-purified *Av*FixABCX, and we assume that the ET-FAD_SQ/HQ_ is the possible redox transition relevant to catalysis (nevertheless, calculations based on the ET-FAD_OX/SQ_ redox transition show a similar trend, as discussed below). Thus, Bf-FAD transfers an electron to the SQ state of ET-FAD at *E*_*SQ/HQ*_ = +20 mV. During electron bifurcation, a sequential electron transfer from Bf-FAD occurs, with the first electron transfer to ET-FAD_SQ_ and the second to Fld_NSQ._ Given that the overall turnover number of the bifurcation reaction is 0.4 × 10^3^ s^–1^ (Table S4), and it is 10^3^ times faster than the calculated electron-transfer rate from Bf-FAD_HQ_ to ET-FAD_SQ_ (0.5 s^–1^) at the 25 Å distance observed in the structure, we assume that a conformational change must occur to decrease the distance between Bf-FAD and ET-FAD.

**Figure 6:**
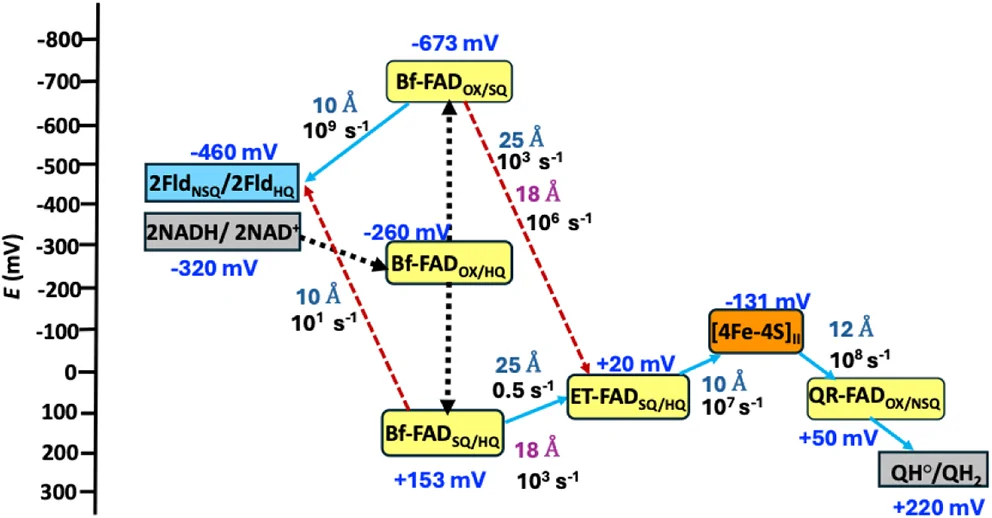
Energy landscape of *Av*FixABCX. Redox couples that are involved in electron transfer are shown. The subscript in each FAD and Fld indicates the redox couple for which the reduction potentials were determined. As described above in the UV–vis redox titration and supported by SWV data, ET-FAD is in the ASQ state because it has a higher reduction potential for the OX/SQ couple than for the SQ/HQ couple. The distances between the cofactors, based on the cryo-EM structure, are shown in light blue font. Distances and the potentials between the electron donor and the acceptor in each electron-transfer step were used to calculate the electron-transfer rates using the Moser-Dutton equation (Supporting Information Table S3). The 18 Å distance shown in purple is calculated based on the turnover number of 10^3^ s^–1^, obtained for the bifurcation activity. Red dashed arrows show possible electron-transfer directions from each redox couple of Bf-FAD. Electron bifurcation takes place at Bf-FAD. Comparison of electron-transfer rates from Bf-FAD to Fld and ET-FAD shows that the SQ/HQ state of Bf-FAD transfers the electron to ET-FAD when ET-FAD and Bf-FAD are 18 Å apart, while the OX/SQ state of Bf-FAD transfers the electron to Fld_NSQ_, and this sequential electron transfer prevents the short-circuiting of the electrons. The significant change in electron-transfer rates with changes in the distance between ET-FAD and Bf-FAD indicates a possible conformationally gated FBEB due to domain II movement.

If we use the turnover number of 0.4 × 10^3^ s^–1^ as the slowest possible electron-transfer rate between Bf-FAD_HQ_ and ET-FAD_SQ_, the minimum distance between them would have to be 18 Å or less. In this scenario, the uphill electron transfer to Fld from Bf-FAD_HQ_ is calculated to occur at ∼10^1^ s^–1^, eliminating the possibility of short-circuiting in the first electron transfer. Presumably, this conformation exists with a longer ET-FAD to [4Fe-4S]_II_ distance than observed in the cryo-EM structures. This generates a Coulomb blockade, and electron transfer from Bf-FAD_SQ_ to ET-FAD_HQ_ cannot occur until ET-FAD_HQ_ transfers its electron to [4Fe-4S]_II_ and for this to occur a conformational change is required back to the observed conformation with an ET-FAD_HQ_ to [4Fe-4S]_II_ distance of ∼10 Å. This gating mechanism then effectively promotes the second electron-transfer event from Bf-FAD_OX/SQ_ to Fld_NSQ_, which we measured by TAS and confirmed by calculation at ∼10^9^ s^–1^. This gating appears to translate to high fidelity in both bifurcation and confurcation reactions, with observed ideal stoichiometries of 2NADH:1Q:2Fld_NSQ_ and 2Fld_HQ_:1QH_2_:2NAD^+^, respectively (Table S5).

Surprisingly, we observe that NADH reduces Q in the absence of Fld_NSQ_, indicating that bifurcation is not obligatory. This reaction occurs much faster than the turnover rate for bifurcation. The rate is so fast that we cannot reliably measure it by stopped-flow spectrophotometry, indicating a rate on the order of 10^5^ s^–1^ or faster. This suggests that the ET-FAD and Bf-FAD distance of 18 Å that we have proposed in our mechanistic model is likely shorter. Using the Moser–Dutton formula with a rate of ∼10^5^ s^–1^ and our determined reduction potentials results in a distance of 14 Å, which is more closely aligned with observed biological electron-transfer distances. Interestingly, if we consider a distance equal to or less than 12 Å, the calculated rates indicate that short-circuiting reactions become more favorable. Therefore, the conformational change that is expected in flexible domain II can move ET-FAD closer to Bf-FAD than 25 Å and move away from Bf-FAD once the first electron transfer takes place to prevent short-circuiting of the second electron. Hence, we experimentally confirm that EB in *Av*FixABCX is a conformationally gated electron transfer, and this conformational change in domain II prevents short-circuiting of the second electron to the higher-potential pathway in the presence of Fld_NSQ_.

### SEC-SAXS Data Show Compact and Extended Conformational States of *Av*FixABCX in Solution

Understanding that EB in *Av*FixABCX requires a conformational change to direct an electron from Bf-FAD to the higher-potential pathway via ET-FAD in domain II, we set out to gather evidence of alternative conformations. Size-exclusion chromatography coupled with SAXS (SEC-SAXS) was performed to investigate solution-state conformational changes associated with catalysis. SAXS is a biophysical method that provides low-resolution information on overall structural shape and large-scale structural transitions of biological macromolecules in solution.^69^ SEC-SAXS helps to separate aggregated proteins from the macromolecular state of interest. Detailed analyses of SEC-SAXS data using evolving factor analysis (EFA) for all the different reaction conditions are provided in Supporting Information Section 1.11 and the results are provided in Figures S11–S20.

Ensemble analysis identified multiple conformational states in solution, including a population resembling the cryo-EM-like structure of *Av*FixABCX (Table S8). All structural models generated from the SAXS samples had χ^2^ values between 1 and 2 (χ^2^ should be closer to 1 but no more than 10).^70^ Conformers with a *D*_max_ larger than 139 Å (which is the *D*_max_ of the cryo-EM structure) are present in the P­(r) plot (or *R*_*g*_ values above 52 Å as tabulated in Table S8) and are referred to as extended conformers and differ in the domain II position compared to the cryo-EM structure. Models that look like the cryo-EM structures are referred to as compact conformers. In the extended conformers, ET-FAD in domain II is spatially separated from both Bf-FAD and [4Fe-4S]_II_ and resides far away. Regardless of the oxidation states of the cofactors in the samples, whether they are air-oxidized, as-purified, or reduced with NADH, the solution state of the *Av*FixABCX sample contains varying degrees of conformers from compact to extended, as shown in Figure 7A and 7B. The as-purified *Av*FixABCX sample had compact conformers with *R*_*g*_ values of 45 and 52 Å, which collectively accounted for 46%; 20% of the conformers had *an R*_*g*_ value of 59 Å, and 24% had an *R*_*g*_ value of 80 Å. Here, conformers with a 59% population showed slight extension of domain II, whereas conformers with *an R*_*g*_ value of 80 Å showed extended domain II, as shown in Figure 7B. The population of extended conformers increased to 42% when 2 eq of NADH was present and was in equilibrium with the compact conformers, which accounted for 58%. After full reduction with NADH (8 eq of NADH added to the sample), the extended conformer population decreased to 15%. The increase in the population of the extended conformer during the reduction could indicate that higher-potential cofactors, Q, QR-FAD, and [4Fe-4S]_II_, are already being reduced by ET-FAD, so domain II stays far away from Bf-FAD and [4Fe-4S]_II_ (as shown in Figure 7D). Surprisingly, when all the components that are required for catalysis are present in the bifurcation or confurcation reaction (*Av*FixABCX, Fld (NSQ/HQ), Q or QH_2_, and NADH/NAD^+^), only the extended conformer is present (Figure 7C). Although our SEC-SAXS data analysis using EFA supports the extended-conformation interpretation, a more highly purified sample may improve and further define the nature of this structure.

**Figure 7:**
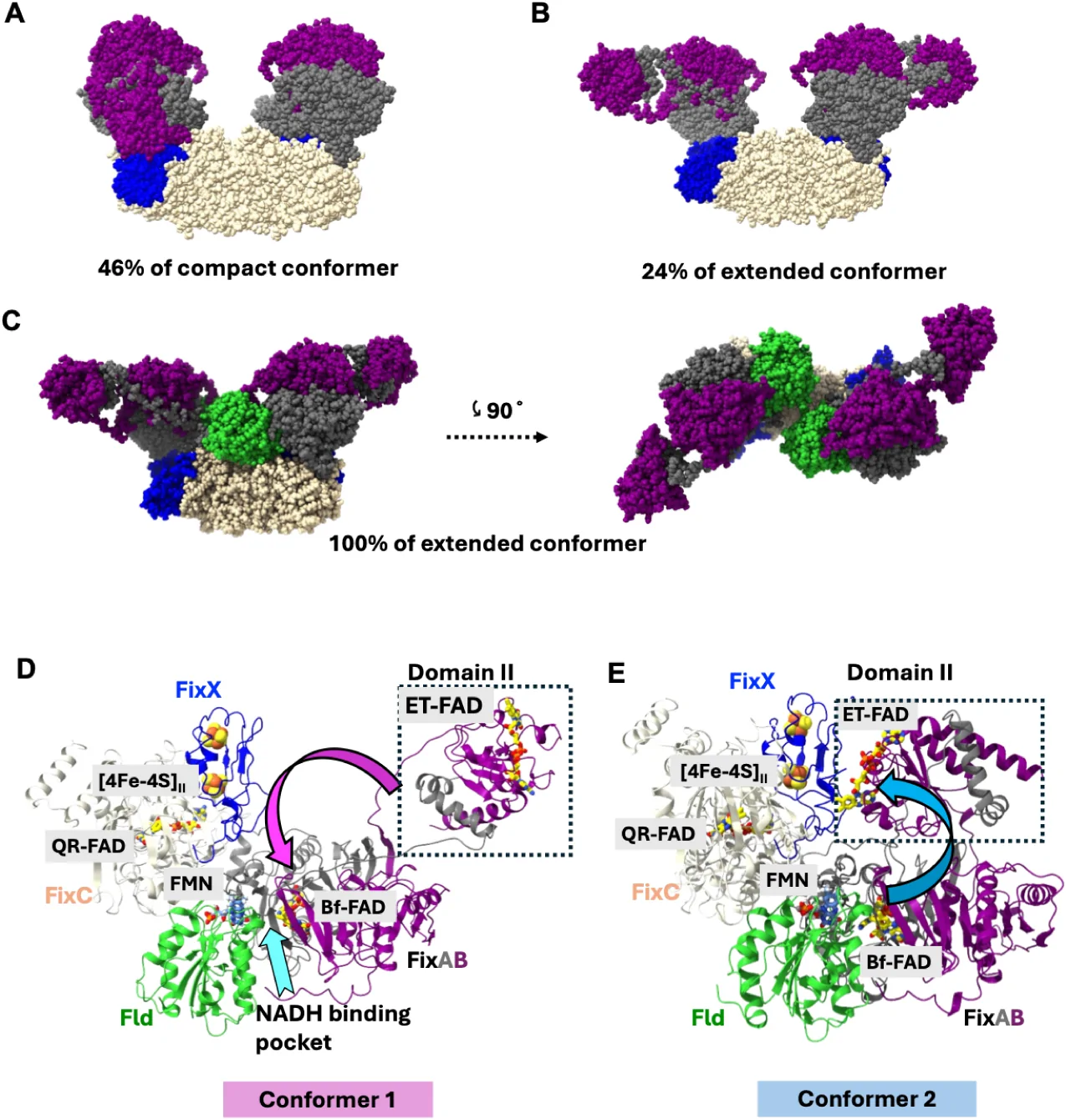
Possible conformational states that enhance the FBEB catalytic cycle in *Av*FixABCX A) and B) two solution-state conformers obtained from BilboMD in two different populations in the as-purified *Av*FixABCX sample based on SEC-SAXS analysis (two more conformers are present with *R_g_* values of 45 Å and 59 Å, as tabulated in Table S8; the structural model in A is a compact conformer similar to *Av*FixABCX cryo-EM structure), and C) two different orientations of the structural model of the extended conformer that exists at 100% when the as-purified *Av*FixABCX sample is treated with Fld_NSQ_, Q, and NADH. Extended conformers show domain II spatially separated from Bf-FAD and [4Fe-4S]_II_ in FixX, while this domain II is mostly stabilized by FixX in the compact conformer and the cryo-EM structure. D) Extended conformational state in *Av*FixABCX (only the tetramer is shown for clarity); a resting state where Fld_NSQ_ binds first, then NADH, NADH binding triggers movement of domain II and brings ET-FAD closer to Bf-FAD; E) compact, cryo-EM structure-like conformational state, where ET-FAD (in the reduced state) in domain II interacts with [4Fe-4S]_II_ of FixX after accepting a single electron from Bf-FAD, the movement of domain II prevents short-circuiting of the second electron and ensures the second electron flows to Fld_NSQ_, the pink curved arrow in (D) shows movement of domain II toward Bf-FAD during catalysis, and the blue arrow in (E) shows movement of the domain II toward FixX but away from Bf-FAD when ET-FAD is being reduced. An intermediate conformational state exists between the extended and compact conformers as described above, in which ET-FAD is 18 Å from Bf-FAD, unlike in the compact structure where it is 25 Å away. This intermediate conformer was not observed in either cryo-EM or SEC-SAXS in this study; however, the FBEB rate verifies its presence, as explained above in the conformational gating mechanism.

The presence of a 100% extended conformer in the bifurcation and confurcation reaction mixture could be due to turnover in these reactions. Therefore, the extended conformer could be the resting state when none of the cofactors need to be reduced, since domain II stays away from the Bf-FAD and [4Fe-4S]_II_ in this state. Collectively, as reported for *Tma*EtfABCX and *A. fermentans* EtfAB solution-state structures, we also observe conformational equilibria in *Av*FixABCX.^70,71^ More importantly, our data provide the sample conditions that generate the highest population of the extended conformer, which could play an important role in the catalytic cycle. In addition, our data show that the NADH reduction of *Av*FixABCX alters the conformational equilibrium between the compact and extended states. We revisited the cryo-EM structure determination to see whether we could find evidence for the extended conformer in that data set, but we were unable to find particles corresponding to the extended structure. This is likely due to the mobility of domain II in the extended structure and suggests that obtaining a high-resolution structure of the extended conformer may be challenging.

Based on the low-resolution structural models we obtained, we propose that *Av*FixABCX undergoes conformational changes in solution due to domain II movement, which is required to bring ET-FAD closer to Bf-FAD during FBEB. The crystal structure of *A. fermentans* EtfAB soaked in NADH shows that the ADP moiety of NADH binds in the Bf-FAD binding pocket, and modeling of ferredoxin with EtfAB shows that ferredoxin binds in the NADH binding pocket as well.^5,55^ Similarly, our modeling of *Av*Fld with *Av*FixABCX shows that Fld binds in the same region shown in the structural model in Figure 7C and that the distance between the FMN and Bf-FAD is 10 Å, a favorable electron-transfer distance. Previously, in Ledbetter et al.‘s work,^17^ based on a cross-linking mass spectrometry (XL-MS) structural model, we proposed that FixX binds near the Bf-FAD binding pocket and Bf-FAD (referred to as a-FAD) transfers an electron to Fld/Fd via the two [4Fe-4S] clusters in FixX during the EB. We now suggest that this misinterpretation likely resulted from multiple conformers and conformational equilibria, as observed in our SAXS data. We repeated the XL-MS experiments in this work, which show cross-links within 30 Å as well as long-range ones over 50 Å, suggesting structural changes within the octamer and/or the presence of multiple conformers in solution in the presence and absence of NADH (Supporting Information Movie S2 and Table S9).

### How Conformational Dynamics in *Av*FixABCX Controls the Bifurcation Activity

One of the major findings of this work is the evidence of conformational movements in *Av*FixABCX associated with electron bifurcation under the conditions required for bifurcation. Previous works in homologous systems report only oxidizing and reducing conditions in the presence of NAD­(H). Our controlled experimental design for steady-state kinetics measurements and SEC-SAXS data, along with the energy landscape, reinforce a mechanism for conformationally gated electron bifurcation in *Av*FixABCX. Superimposition of compact and extended structural models (models in Figure 7A and 7C) reveals a significant conformational change in the NADH and Fld binding pockets, driven by domain II movement. This finding is consistent with work reported on the superimposition of *C. difficile* EtfAB conformational states.^55^ They report that a conformational change near the NADH (and Fld/Fd) binding pocket could be induced by NADH binding, which triggers the domain II movement from an open conformer to a closed conformer (B-like state) where the ET-FAD is at a favorable distance to Bf-FAD to accept an electron.^55^ Three main amino acid segments (see Figures S21A and S21B) near the NADH and Fld binding pockets of *Av*FixABCX, similar to those reported in EtfAB in *C. difficile*, may play an important role in catalysis: the EtfB/FixA protrusion, EtfB/FixA arm anchor, and EtfA/FixB hairpin (Supporting Information Figure S21). A conserved amino acid T227 in the EtfAB-Bcd complex of *C. difficile* ^55^ (T229 in *Av*FixABCX), which is 3.8 Å away from Bf-FAD (referred to as β-FAD in the article), undergoes a 2 Å shift when NADH binds, pushing the EtfA/FixB hairpin away from the binding pocket, which then disengages the EtfB/FixA protrusion from the EtfA/FixB hairpin. Simultaneously, the EtfB/FixA arm is pulled into a position where domain II is closer to Bf-FAD (similar to what is shown in Figure 7D). Also, mutational studies of these segments have shown a significant change in the bifurcation reaction rates of the EtfAB-Bcd complex in *C. difficile*.^55^ The valuable assumption that was made by the researchers in the EtfAB-Bcd system can be applied to explain the conformationally gated FBEB *Av*FixABCX. Our steady-state kinetic data and the two major conformers observed in the SAXS data during catalysis validate this assumption.

As reported for the *A. fermentans* EtfAB-Bcd complex (a homologue of the EtfAB-Bcd complex in *C. difficile* ^55^), *Av*FixABCX also shows a slower bifurcation reaction than the confurcation reaction.^5^ The rate of the electron transfer that we measured in the bifurcation direction is ten times slower than the rate of confurcation in *Av*FixABCX; this could be due to the fact that when Fld_NSQ_ is bound at the Fld/NADH binding pocket in FixA, the Fld_NSQ_ interaction with the FixA protrusion and anchor arm (as shown in Supporting Information Figure S21B) slows down the domain II movement. However, in the absence of Fld_NSQ_, the FixA protrusion and anchor arm are free and could allow faster domain II movement, leading to faster electron transfer from NADH to Q, as we observed in the steady-state kinetic experiment. Therefore, for an efficient electron bifurcation to take place instead of unidirectional rapid electron transfer from NADH to Q, Fld_NSQ_ binds to *Av*FixABCX first, and then NADH binds. TAS data also show no change in intramolecular electron transfer rates when Fld_NSQ_ binds to *Av*FixABCX in the absence of NADH, suggesting that NADH binding controls the electron flow by triggering the conformational change. The drastic decrease in the electron transfer rate in the bifurcation direction when Fld_NSQ_ binds to *Av*FixABCX, compared with the extremely fast electron transfer from NADH to Q in the absence of Fld_NSQ_, can be explained by our solution-state conformers. According to our SEC-SAXS data, when Fld_NSQ_ is bound to *Av*FixABCX under bifurcation and confurcation conditions, *Av*FixABCX is in the 100% extended conformer. This is after the turnover of the reactions. Therefore, this could be the conformational state in which ET-FAD in domain II stays far from Bf-FAD and [4Fe-4S]_II_ when electron transfer from Bf-FAD to [4Fe-4S]_II_ is not needed; the system is in the resting state (as shown in Figure 7C and 7D). During electron bifurcation, first Fld_NSQ_ binds to the extended conformer, then NADH binds to transfer 2e^–^ to the Bf-FAD. This triggers a conformational change that positions domain II closer to the Bf-FAD (as shown by pink curved arrows in Figure 7D), where ET-FAD is 18 Å from Bf-FAD. Once ET-FAD is near the 2e^–^ reduced Bf-FAD, it accepts a single electron from the Bf-FAD_SQ/HQ_; then domain II swings back to the compact structure to interact with [4Fe-4S]_II_ in FixX for electron transfer (as shown by blue curved arrows in Figure 7E). This movement prevents the second electron from going to the higher-potential ET-FAD and prevents short-circuiting, thereby facilitating the second electron transfer from Bf-FAD_OX/SQ_ to Fld_NSQ_. Energy released from the higher-potential pathway enhances the energetically unfavorable electron transfer to Fld_NSQ_ at a rate of 10^9^ s^–1^. This movement of domain II may be transient, and we may not be able to observe the shortest distance between Bf-FAD and ET-FAD using solution-state or higher-resolution structures. During confurcation, electron transfer from Fld_HQ_ to Bf-FAD (−260 mV) is energetically more favorable than from Fld_HQ_ to Fld_NSQ_ because the reduction potential of Fld_HQ/NSQ_ is −460 mV, lower than the reduction potential of Fld_NSQ/OX_, which is −187 mV.^36^ Once Fld_HQ_ delivers the single electron to Bf-FAD, it becomes Fld_NSQ_. This Fld_NSQ_ species is not necessarily bound to the binding pocket as the reaction moves in the reverse direction, and electron transfer from Fld_NSQ/OX_ (−187 mV) to Bf-FAD_OX/SQ_ (−673 mV) is not energetically favorable and is not expected to occur. Therefore, domain II movement is interrupted less than in the bifurcation direction. This can explain why confurcation is 10 times faster than electron bifurcation. Also, for the individual half-reaction in the confurcation direction, in the absence of Fld_HQ_, the rate of electron transfer from QH_2_ to NAD^+^ is 3 times slower than the overall confurcation reaction rate. In summary, Fld_HQ_ binding enhances the confurcation reaction rate, and Fld_NSQ_ binding controls bidirectional electron flow during bifurcation. Therefore, the two major conformational changes driven by domain II movement (Figure 7D and 7E) in *Av*FixABCX are likely essential for the efficient EB mechanism.

## Conclusion

In this work, we systematically determined the electron bifurcation mechanism of *Av*FixABCX using the cryo-EM structure, UV–visible spectroscopy, EPR spectroscopy, steady-state kinetic measurements of bifurcation and confurcation reactions, and electrochemical properties of redox cofactors. The combination of these techniques facilitated the elucidation of the energy landscape in bifurcating *Av*FixABCX. We have shown that the FixX [4Fe-4S] clusters exist at disparate potentials, with a higher-potential cluster that apparently functions in electron transfer and a lower-potential cluster that does not. This was not anticipated; the functional role of this low-potential [4Fe-4S] cluster remains unclear. Compact and extended forms of *Av*FixABCX exist in the solution state to carry out the catalytic cycle. NADH-binding-induced domain II movement in the extended conformer could be the rate-limiting step when Fld_NSQ_ is present, whereas releasing domain II from the NADH-binding pocket to reach the compact structure could be the fastest step to prevent short-circuiting of the second electron during EB. Although previous studies of the EtfABCX and EtfAB-Bcd homologues report that a conformationally gated mechanism is required to carry out FBEB, they did not experimentally compare the intrinsic rates of the individual reaction steps with the overall reaction rate to support the claim that FBEB in bifurcating Etfs requires a conformational change. In this work, we present strong evidence of a catalytic step that requires conformational gating in bifurcating Etfs. The proposed mechanism is based on measured redox potentials, the energy landscape, the rates of individual reaction steps, the overall FBEB reaction rate, and solution-state structures. While the catalytic mechanism of FBEB in bifurcating Etfs requires a larger conformational change, NfnSL requires only a subtle conformational change when NADPH binds near L-FAD. This is because in NfnSL, redox cofactors are within favorable electron-transfer distances, unlike in Etfs. NfnSL and *Av*FixABCX show the presence of crossed potentials in the bifurcating FADs. However, NfnSL has a larger energy gap compared with bifurcating Etfs and *Av*FixABCX. This larger energy gap likely contributes to the prevention of short-circuiting of electron transfer during FBEB. Collectively, *Av*FixABCX carries out a conformationally gated FBEB reaction. This kinetically and thermodynamically controlled FBEB mechanism enhances the efficiency of the electron bifurcation.

## Supplementary Material







## Notes

All the experimental details and materials and methods; cryo-EM data collection, processing, and model refinement; SEC profile, SDS-PAGE gels and UV–vis and EPR data; SEC-SAXS data analysis, ensembles, and populations of models generated from BilboMD; steady-state kinetic results, UV–visible spectra, and spectral deconvolution; TAS kinetic traces (PDF)
Movie S1: 3D variability analysis in cryo-EM particles (MP4)
Movie S2: 3D variability analysis in long-range BS3 cross-links (MP4)

## Abbreviations

EB: electron bifurcation
FBEB: flavin-based electron bifurcation
Av: *Azotobacter vinelandii*
Q: quinone
OX: oxidized
SQ: semiquinone
HQ: hydroquinone
FeS: iron–sulfur
FAD: flavin adenine dinucleotide
Etf: electron transfer flavoprotein
ASQ: anionic semiquinone
NSQ: neutral semiquinone
ETC: electron transport chain
ET-FAD: electron transfer FAD
Bf-FAD: bifurcating FAD
QR-FAD: quinone reductase FAD
