# Supplementary material for: Elucidation of the Azotobacter vinelandii FixABCX Energy Landscape Reveals a Conformationally Gated Flavin-Based Electron Bifurcation
Source: J Am Chem Soc. 2026 Oct 29;148(44):47867–83. doi: 10.1021/jacs.6c11513 (PMC13761266; doi:10.1021/jacs.6c11513)
Supplement: Supplementary file 1 [file ja6c11513_si_001.pdf]

Supporting Information

Elucidation of the *Azotobacter vinelandii* FixABCX energy landscape reveals a conformationally gated flavin-based electron bifurcation.

Nishya Mohamed-Raseek[1], Dmitriy A Lukoyanov[2], J. Luke Findlay[3], Florence Mus[1], Seth A. Wiley[4], Monika Tokmina-Lukaszewska[3], Farnaz Ghorbani[1], Carolyn E. Lubner[4], Brian Bothner[3], C. Martin Lawrence[3], Brian M. Hoffman[2], John W. Peters[1]*.

[1]Department of Chemistry and Biochemistry, The University of Oklahoma, Norman, Oklahoma 73019, United States.
[2]Department of Chemistry, Northwestern University, Evanston, Illinois 60208, United States.
[3]Department of Chemistry and Biochemistry, Montana State University, Bozeman, Montana 59717, United States.
[4]National Laboratory of the Rockies, Biosciences Center, Golden, Colorado 80401, United States.

*Corresponding author: John W. Peters: jw.peters@ou.edu

Table of Contents

1. MATERIALS AND METHODS **4**

1.1. Construction of *Av*fixABCX, *Av*fixX, *Av*fixAB, *Av*fixCX and *Rcas*fixX 4

1.2. Recombinant Protein Expression and Purification 4

1.2.1. AvFixABCX and AvFixCX *4*

1.2.2. AvFixX and RcasFixX *5*

1.2.3. AvFixAB expression and purification *5*

1.3. Protein concentration determination and Cofactor quantification. 6

1.4. FAD and FeS cluster reconstitution 7

1.5. *Av*FixABCX Cryo-EM data collection and structure determination 7

1.6. Anaerobic reduction of as-purified and air-oxidized *Av*FixABCX with NADH 8

1.7. Electrochemistry 8

1.8. Potentiometric Titration of *Av*FixABCX 9

1.9. EPR data collection for *Av*FixABCX, CuSO4 and Nernst fit 10

1.10. EPR radical quantification in *Av*FixABCX using $CuSO_4$ standard curve 10

1.11. Size Exclusion Chromatography coupled with Small Angle X-ray scattering (SEC-SAXS) data collection and processing. 11

1.14. Crosslinking Mass spectrometry (XL-MS) 15

1.15. Transient absorption spectroscopy (TAS) 15

1. RESULTS: **16**

Table S1: The list of primers used to amplify the *Av*FixABCX and *Av*FixAB genes. 16

Table S2: cryo-EM data collection, processing, model refinement and validation. 16

Table S3: Moser-Dutton equation parameters and rates of the reaction 18

Table S4: Calculated non-equilibrium reduction midpoint potentials. 18

Table S5: Activity assay of each individual reactions in the forward, reverse direction, confurcation and bifurcation reactions, stoichiometric coefficient between the reactants and free energy change of each reaction. 19

Table S6: Fitting parameters for the data shown in Figure S8. 19

Table S7: Primary analysis of SAXS profiles using RAW and GNOM from ATSAS package 20

Table S8: Selected ensembles from BilboMD and populations of models. 20

Table S9: Protein-protein interactions captured with BS3 within FixABCX complex. 21

Figure S1. A) An exemplar micrograph 24

Figure S2: Single particle reconstruction workflow for *Av*FixABCX. See Methods for details. 26

Figure S3: Local Resolution and Orientation Diagnostics 28

Figure S4: Model Fit to Map (Stereo Figure). 29

Movie S1: 3D variability analysis in *Av*FixABCX cryo-EM particles. 30

Figure S6: SEC profile, SDS PAGE gels, UV-visible and EPR characterization of *Av*FixABCX 31

Figure S7: Amino acid arrangement near [4Fe-4S] clusters in *Av*FixX 32

Figure S8. Kinetic traces 33

Figure S9:UV-visible spectral change during the steady-state kinetic reactions and the steps of the spectral deconvolution. 34

Figure S10: UV-visible spectral change during the bifurcation reaction and the lower-potential half-reaction: The red spectrum shows the spectral change during the lower-potential half-reaction mixture containing *Av*FixABCX, Fld_{NSQ}, and NADH. Absorbance at 340 nm is due to NADH consumption, absorption at 450 nm is due to the change in the absorption of flavins in *Av*FixABCX, and a small absorption change at 580 nm is due to the consumption of Fld_{NSQ}, which is negligible. However, in the blue spectrum, the bifurcation reaction mixture containing *Av*FixABCX, Fld_{NSQ}, NADH, and Q shows a relatively visible spectral change in all components. This indicates the formation of QH_2 at 280 nm, Fld_{HQ} from Fld_{NSQ} at 580 nm, and the consumption of NADH. Here, the absorption at 280 nm includes the change in absorption due to the conversion of Fld_{NSQ} to Fld_{HQ}, which is accounted for in the determination of QH_2 concentration. 35

Figure S11: Comparison of SEC chromatogram and SEC-SAXS scattering profiles. 36
Figure S12: A, B and C) SVD of three overlapping peaks in the as-purified AvFixABCX SEC-SAXS profile shows six scattering components. 37
Figure S13: Evolving factor analysis (EFA) of as-purified AvFixABCX sample; 38
Figure S14: A, B, C, D, E, and F) EFA analysis of the as-purified AvFixABCX sample by picking the region with a higher singular value. 39
Figure S15: A, B and C) SVD of the entire elution profile in the sample containing as-purified AvFixABCX with AvFldNSQ, NADH and Q shows seven scattering components. 40
Figure S16: Evolving factor analysis (EFA) of as-purified AvFixABCX with AvFldNSQ, NADH, and Q; 41
Figure S17: A, B, C, D, E, and F) EFA analysis of the as-purified AvFixABCX with AvFldNSQ, NADH, and Q by picking the region with a higher singular value. 42
Figure S18: 43
Figure S19: P(r) function and dimensionless Kratky plot. 43
Figure S20: DENSS maps generated for all the SAXS curves using RAW 44
Figure S21: Amino acid segments surrounding the NADH binding pocket near the Bf-FAD site of *Av*FixABCX 45
Movie S2. Long-range BS3 crosslinks detected between domain II (FixB) and FixC, FixA and FixX in the presence and absence of NADH. 46
REFERENCES 47

1. Materials and Methods

1.1. Construction of *Av*fixABCX, *Av*fixX, *Av*fixAB, *Av*fixCX and *Rcas*fixX

The fixA (Avin_10520), *fixB* (Avin_10530), *fixC* (Avin_10540), and *fixX* (Avin_10550) genes from *Azotobacter vinelandii* (DJ, TAX 322710) were amplified using forward and reverse primers for FixABCX listed in Table S1 of the supporting information and cloned into the EcoRI and BamHI

restriction sites of the pASK-IBA7C vector containing an inducible tetracycline promoter to generate the *Av*FixABCX plasmid construct. An N-terminal Strep-tag II with a factor Xa protease cleavage site is present at *the fixA* gene. Amplified *fixA* and *fixB* genes were cloned into the pETDuet™-1 vector to make the *Av*FixAB plasmid construct. *The fixA* gene was cloned after the N-terminal 6X His tag in the vector at multiple cloning site 1 (MCS-1) by digesting the vector at the BamHI and HindIII restriction sites, whereas *fixB* was cloned in MCS-2 between NdeI and KpnI. The *fixX* gene with a C-terminus TEV-MBP-Strep-tag II was synthesized and cloned into the MCS-2 restriction sites NdeI and XhoI in the pCDFDuet™-1 vector by GenScript to generate *the Av*FixX plasmid construct. In a separate construct, the *fixC* gene with an N-terminal Strep-tag II was synthesized and cloned into MCS-1 between NcoI and BamHI, whereas *fixX* was cloned into MCS-2 at the BamHI and HindIII restriction sites with no tag in the pCDFDuet™-1 vector by GenScript to generate *the Av*FixCX plasmid construct. The *Roseiflexus castenholzii* (*Rcas*) *fixX* construct from the previously published work of Ledbetter *et al.* was used to express *Rcas*FixX to compare the redox properties of two [4Fe-4S] clusters in FixX[1].

1.2. Recombinant Protein Expression and Purification

1.2.1. *Av*FixABCX and *Av*FixCX

Plasmid pASK-IBA7C::*fixABCX* was transformed into BL21(DE3)-Δ*isc*R cells for heterologous overexpression. Cells were grown in TB medium (18 L) supplemented with 20 mg/L riboflavin, 2 mM $MgCl_2$, and 50 µg/ml chloramphenicol at 37 °C up to OD_{600} 0.4-0.5 in screw-cap flasks covered with aluminum foil. At this point, the cell culture was supplemented with ammonium Fe (III) citrate (4 mM), L-cysteine (2 mM) to increase Fe-S cluster occupancy, and sodium fumarate (10 mM), which serves as a terminal electron acceptor for anaerobic metabolism. Aluminum foil was replaced by screw caps with rubber sealing. A long needle was inserted through the rubber seal to sparge Ar, and a small needle was used to vent the gas. After sparging Ar for 15-30 min to remove residual O_2, protein expression was induced by adding 200 µg/L anhydrotetracycline, and cells were incubated overnight at room temperature while sparging Ar. Cells were anaerobically transferred to airtight centrifuge tubes in a COY chamber and centrifuged at 10, 000 g for 10 min. Cell lysis and protein purification were carried out under anaerobic conditions in the COY chamber (95 % N_2, 5% H_2). Wet cell pellets were suspended in degassed Bug Buster containing 10 µg/ml DNase, EDTA-free protease inhibitor (Cat no. A32965, 1 tablet/50 mL), 1 mM Flavin adenine dinucleotide, and 1 mM DTT (1:2 ratio of mass of cell pellet to Bug Buster). Resuspended cells were stirred gently in the COY chamber for 1-2 h. Then 1% DDM was added dropwise to solubilize the membrane-bound subunit FixC, and the mixture was gently agitated for 1 h; the supernatant was collected by centrifugation at 15,000 g for 30 min. *Av*FixABCX protein was purified using a prepacked Strep-Tactin^R^XT 4Flow (IBA Cat no. 2-5024-001) FPLC column using the AKTA FPLC. The column was pre-equilibrated with buffer containing 50 mM Tris-HCl (pH 8.5), 150 mM NaCl, 10% glycerol, 0.02% DDM, and 1 mM DTT. The buffer was degassed by sparging with N_2 prior to adding 0.02% DDM and 1 mM DTT in the COY chamber. The buffer was kept under Ar throughout the purification using AKTA pure™ FPLC. Once the protein was loaded, the column was washed until the baseline absorbance returned. The bound protein was then eluted with buffer containing 50 mM Tris-HCl (pH 8.5), 150 mM NaCl, 10 % glycerol, 0.02 % DDM, 1 mM DTT, and 50 mM biotin as a Yellow-brown band and anaerobically buffer exchanged to 50 mM Tris-HCl (pH 8.5), 150 mM NaCl, 10 % glycerol, 0.02 % DDM and 1 mM DTT using 10DG desalting column (BIO RAD, Cat no. 7322010), then concentrated using an Amicon (EMD Millipore) centrifugal filter with a membrane molecular mass cutoff 30 kDa. Size exclusion chromatography (SEC) was conducted using Superdex® 200 Increase 10/300 GL (Cytiva, 28-9909-44) to monitor heterogeneity in the *Av*FixABCX protein. 5-7 mg/ml protein was run at 0.5 ml/min speed. To estimate the molecular weight of SEC peaks of *Av*FixABCX, chromatography standards (Bio-Rad, Cat no.1511901) were run on the same column under the same conditions. Purified

*Av*FixABCX protein was aliquoted in the COY chamber into airtight screw cap tubes and stored at -80 °C until further use.

Plasmid pCDFDuet™-1::*fixCX* was expressed, purified and handled as same as *Av*FixABCX except cells were grown in the media supplemented with 100 µg/ml streptomycin up to the OD_{600} = 0.2-0.3 and *Av*FixCX protein expression was induced by adding 0.5 mM IPTG.

1.2.2. *Av*FixX and *Rcas*FixX

*Av*FixX was anaerobically expressed in BL21(DE3)-*Δisc*R cells, like *Av*FixABCX, except that the cells were grown in TB medium supplemented with 100 µg/ml streptomycin without added riboflavin, and the protein expression was induced by adding 1mM IPTG. Protein purification was carried out by resuspending the cells anaerobically in the degassed lysis buffer containing 50 mM Tris-HCl (pH 8.0), 150 mM NaCl, 10 % glycerol, and 1 mM DTT, supplemented with 10 µg/ml DNase and an EDTA-free protease inhibitor (1 tablet/50 mL). The mass of the cell pellet to the volume of lysis buffer is a 1:5 ratio. The buffer was degassed by sparging with N_2 prior to adding 1 mM DTT in the COY chamber. Resuspended cells were lysed using the sonicator in a COY chamber (95 % N_2, 5% H_2) and the supernatant was collected by centrifugation at 15, 000 g for 30 min. *Av*FixX protein was purified using prepacked Strep-Tactin® superflow® high capacity FPLC column (IBA, Cat no. 2-1258-001) using AKTA pure™ FPLC. Column was pre-equilibrated with buffer containing 50 mM Tris-HCl (pH 8.0), 150 mM NaCl, 10 % glycerol, 1 mM DTT. The buffer was kept under Ar throughout the purification. After loading the protein, the column was washed until the baseline absorbance returned. The bound protein was then eluted with buffer containing 50 mM Tris-HCl (pH 8.0), 150 mM NaCl, 10 % glycerol, 1 mM DTT, and 2.5 mM desthiobiotin as a brown band and anaerobically buffer exchanged to 50 mM Tris-HCl (pH 8.0), 150 mM NaCl, 10 % glycerol and 1 mM DTT using 10DG desalting column (BIO RAD, Cat no. 7322010), then concentrated using an Amicon (EMD Millipore) centrifugal filter with a membrane molecular mass cutoff 10 kDa. Purified *Av*FixX protein was aliquoted in the COY chamber into airtight screw cap tubes and stored at -80 °C until further use. *Rcas*FixX was expressed and purified as described before[1].

1.2.3. *Av*FixAB expression and purification

Plasmid pETDuet™-1::*fixAB* was transformed into BL21(DE3)-Δ*isc*R cells for heterologous overexpression. Cells were grown in TB medium supplemented with 20 mg/L riboflavin, 2 mM $MgCl_2$, and 100 µg/ml carbenicillin at 37 °C up to OD_{600} 0.2-0.3 in screw-cap flasks covered with aluminum foil. Aluminum foil was replaced by screw caps with rubber sealing. A long needle was inserted through the rubber seal to sparge Ar, and a small needle was used to exhaust the gas. After sparging Ar for 15-30 min to remove residual O_2, protein expression was induced by adding 0.5 mM IPTG, and cells were incubated overnight at room temperature while sparging Ar. Cells were anaerobically transferred to airtight centrifuge tubes in a COY chamber and centrifuged at 10, 000 g for 10 min. Anaerobic expression of *Av*FixAB helped eliminate the major contaminant protein, likely ferritin. Cell lysis and protein purification were carried out under aerobic conditions. Wet cell pellets were suspended in Bug Buster containing 10 µg/ml DNase, EDTA-free protease inhibitor (1 tablet/50 mL), and 1 mM Flavin adenine dinucleotide (1:2 ratio of mass of cell pellet to Bug Buster). Resuspended cells were stirred gently for 1- 2 h. Then, the supernatant was collected by centrifugation at 15, 000 g for 30 min. *Av*FixAB protein was purified using a Bio-Rad Ni-IMAC prepacked column using the AKTA pure™ FPLC. The column was pre-equilibrated with an equilibration buffer containing 50 mM Tris-HCl (pH 8.0), 150 mM NaCl, 10 % glycerol. After loading the protein, the column was washed with buffer containing 50 mM Tris-HCl (pH 8.0), 150 mM NaCl, 10% glycerol, and 20 mM imidazole until the baseline absorbance returned. The

protein was then eluted with buffer containing 50 mM Tris-HCl (pH 8.0), 150 mM NaCl, 10% glycerol, and 500 mM Imidazole as a yellow band and buffer exchanged to 50 mM Tris-HCl (pH 8.0), 150 mM NaCl, 10 % glycerol using a 10DG desalting column (Bio-Rad, Cat no. 7322010) with a molecular weight cutoff of 10 kDa. Purified protein was concentrated and passed through a size-exclusion column (Superdex® 200 Increase 10/300 GL; Cytiva, 28-9909-44) at 0.5 ml/min to separate lower-molecular-weight contaminants. Fractions containing *Av*FixAB were then concentrated using an Amicon (EMD Millipore) centrifugal filter with a membrane molecular mass cutoff 10 kDa. The aliquoted *Av*FixAB sample was stored at -80 °C until further use.

1.3. Protein concentration determination and Cofactor quantification.

An anaerobically sealed, frozen protein sample was placed in the COY chamber and thawed in a cooling block at 4 °C for protein concentration determination and other experiments. Protein concentration was determined using the Pierce Detergent Compatible Bradford assay kit (Cat. No. 23246). Each FixABCX tetramer MW (130 kDa, Strep tag II) was used to convert protein concentration from µg/ml to µM. The absorbance spectrum of the as-purified or air-oxidized *Av*FixABCX sample (1 mL of 10- 15 µM) was recorded, and the protein concentration was determined. Then, 0.100 mL of 75 mM ascorbic acid and 10 µl of 100% trichloroacetic acid (TCA) were added, and the mixture was incubated at 90- 100 °C in a heating block for 10-15 min. The sample was then centrifuged to remove the precipitated protein, and the supernatant was carefully collected for UV-Vis spectra recording. The absorption spectrum of the supernatant was recorded using an Agilent Technologies Cary 6000i UV-Vis-NIR spectrophotometer. Absorption at 450 nm and an extinction coefficient $\varepsilon = 11.3\ mM^{-1}\ cm^{-1}$ were used to calculate the FAD concentration in *Av*FixABCX. Total FAD content is compared with protein concentration. Acidic heat denaturation was performed as described in Aliverti *et al*[2]. to maintain Fe in Fe^{2+} form. This will eliminate the spectral contribution of FeS clusters at 420 nm when quantifying FAD at 450 nm, and the sample can be used to measure Fe content. The same sample was used to measure the Fe content following the colorimetric assay described by Fish W.W[3]. Protein sample and Fe standards (Cat. no 047282.AE) were treated similarly, and the absorbance of magenta colored Fe(II) [Ferrozine] complex was measured at 562 nm. A standard curve of different concentrations of Fe standard was generated to determine the Fe content in *Av*FixABCX, *Av*FixX, and *Av*FixCX samples.

The absorbance spectrum of the FixAB sample with a known concentration was recorded, and the sample was incubated at 90- 100 °C in a heating block for 10-15 min. The sample was then centrifuged to remove the precipitated protein, and the supernatant was carefully collected to record the UV-Vis absorption spectrum. The absorbance at 450 nm and an extinction coefficient of $11.3\ mM^{-1}\ cm^{-1}$ were used to calculate the FAD concentration in the *Av*FixAB sample[2].

1.4. FAD and FeS cluster reconstitution

As the purified *Av*FixAB sample had lower FAD content, the protein sample was reconstituted with 1 mM FAD and incubated at 4 °C overnight after purification using a size-exclusion column. Unbound FAD was removed using a gel filtration column, and FAD content was measured as explained above.

As purified *Av*FixX and *Av*FixCX had lower Fe content, the purified protein samples were buffer-exchanged into 50 mM Tris-HCl (pH 8.0 for *Av*FixX and pH 8.5 for *Av*FixCX), 150 mM NaCl, 10% glycerol, 5 mM DTT, and concentrated to 50-100 µM. Fe reconstitution was carried out under strictly anaerobic conditions in a COY chamber following the protocol described in Freibert *et. a*[4].

1.5. *Av*FixABCX Cryo-EM data collection and structure determination

For cryo-TEM sample preparation, Quantifoil 300 mesh copper R 1.2/1.3 holey carbon grids were plasma cleaned for 45 s at 15 mA with a PELCO easiGlow discharge cleaning system (Ted Pella) and placed into a Vitrobot Mark IV (Thermofisher) blotting apparatus at 4 °C and 95% humidity with Whatman grade 1 blotting paper. Four µL of anaerobically prepared "as purified" FixABCX (3.5 mg/ml) was applied to each grid, blotted for 4 s at blot force 4, and immediately vitrified by plunge-freezing into liquid ethane. During this process, each sample was exposed to air for less than 10 seconds. The sample buffer contained 1 mM DTT to prevent oxidation; under these conditions, oxidation requires more than 10 minutes in air when the sample is stirred, as monitored spectroscopically. The samples are exposed to air for less than 10 seconds. Since we are not mixing these small samples, and the protein takes so long to oxidize in air, we think it is unlikely that the samples have further oxidized. Upon freezing, the samples were clipped into autogrids and stored in liquid nitrogen until analyzed.

For cryo-TEM data collection, prepared grids were loaded into the Talos Arctica G2 transmission electron microscope (ThermoFisher Scientific) operating at 200 kV and tuned for parallel illumination at the Montana State University Cryo-EM Core facility[5]. Micrographs were collected using a Gatan K3 camera and a total electron exposure of 65 e^{-} $Å^{-2}$ distributed over 50-frame dose-fractionated movies with a 3.49 s exposure time. SmartScope-controlled SerialEM was used to collect 3,436 exposures at a nominal magnification of 45,000 (0.9061 Å $pixel^{-1}$) with target defocus values from -0.8 to -2.4 µm using a 5x5 multi-shot scheme, or beam-image-shift (BIS) distance of 7.5 µm [6, 7]. Data collection and initial processing were done with CryoSPARC Live. Single-particle reconstruction was performed with CryoSPARC[8, 9]; the workflow is presented schematically in Figures S1 and S2. Each recorded movie underwent patch motion correction and patch CTF correction in CryoSPARC Live using default parameters. We discarded 537 movies based on quality and CTF-fit resolution, calculated defocus, total motion, and relative ice thickness, leaving 2,899 for subsequent work. The cryoSPARC blob picker was used to select 1,593,178 blobs, extracted with a box size of 352 pixels and binned by 2 (final box size = 176 pixels). In addition to single particles, the grids also contained significant amounts of aggregated and/or denatured material. Iterative 2D classification identified a large number of junk classes containing more than 1,400,000 particles that were culled from the particle stack. The remaining 150,037 particles were subjected to a second round of 2D classification, and 8 of 50 particle classes with 64,538 particles were then used to seed a 2-class *ab initio* reconstruction with C1 symmetry. The 48,303 particles in class 0 gave a map with clear similarity to the 2-fold symmetric *T. maritima* Fix/EtfABCX structure (PDB 7KOE) [10], and subsequent homogeneous refinement with C2 symmetry produced an initial map at 7.27 Å resolution. These particles were then used in Topaz (particle diameter = 150 Å) to train and extract a new set of 239,073 particles. 2D classification and selection (37 of 50 classes) reduced this to 202,250 particles. These particles were then washed with a 2-class ab initio, giving 137,677 particles that upon non-uniform refinement yielded a 5.16 Å map. 3D classification then identified 67,249 particles that, upon final non-uniform refinement, gave a 4.72 Å map by gold-standard Fourier shell correlation (FSC) with a 0.143 cutoff. 3D FSC analysis[11] showed a conical FSC Area Ratio (cFAR) of 0.65 with minimum and maximum directional resolutions of 4.4 and 4.98 Å, while Fourier sampling analysis [12, 13] yielded a Sampling Compensation Factor (SCF) of 0.965 (Figure S2).

A homology model of the hetero octamer, including one FAD molecule in each FixA, FixB, and FixC subunit, was generated using AlphaFold[14]. The model was initially placed into the sharpened map with Phenix (phenix.autosharpen, phenix.dock_in_map)[15]. This was followed by rigid-body refinement of the individual domains (phenix.real_space_refine)[16, 17], and then rigid-body refinement using subdomain fragments identified by the TLS Motion server [18] or by eye. The model was completed using iterative rebuilding in Coot [19] and real-space refinement (phenix.real_space_refine) with Phenix [17, 20]. During late-stage refinement, two [4Fe-4S] clusters were placed by hand into each FixX subunit. Phenix real-space

refinement included global minimization, local grid search, and atomic displacement parameters. Refinement also enforced secondary structure and Ramachandran restraints on the main chain. In later stages, starting model restraints on the main chain and side chains were also used, with fixed outliers (sigma = 1.0 and limit = 15). In addition, Cys S-Fe bond distances were restrained to 2.3 Å (sigma 0.2). While the GSFSC for the map itself is 4.72 Å, the overall map to model FSC with a 0.5 cutoff was 5.0 Å, reflecting the difficulty of refining an atomic model against a low-resolution map, likely due to the conformational and/or compositional heterogeneity of the "as purified" FixABCX. Regardless, by eye, the model fits the map nicely (Figure S4), as indicated by the overall map-to-model correlation coefficient (CC mask) of 0.72. For additional refinement and model statistics, please see supporting information Table S2. The final model was deposited in the Protein Data Bank with accession code PDB ID 9ZQZ and the map was deposited in the Electron Microscopy Data Bank with accession code EMD-74585. Metrics for model and map validation are presented in Table S2. Figures were prepared with Pymol [21], Chimera and ChimeraX [22, 23].

1.6. Anaerobic reduction of as-purified and air-oxidized *Av*FixABCX with NADH

Redox titration was carried out in a buffer containing 50 mM Tris-HCl (pH 8.5), 150 mM NaCl, 5% glycerol, 0.02% DDM, and 1 mM DTT by adding NADH sequentially. As-purified *Av*FixABCX was air-oxidized while monitoring absorbance with an Agilent Technologies Cary 6000i UV-Vis-NIR spectrophotometer until absorbance no longer increased. The protein sample was then carefully degassed to remove residual oxygen. The UV-Vis spectrum of 13 µM air-oxidized *Av*FixABCX was recorded in an airtight, self-masking quartz cuvette with a screwcap and septa (Starna spectrophotometer cells, Cat. no. 18B-Q-10-GL14-S). Then, the reduction titration was carried out in the airtight cuvette (with a continuous flow of Ar gas) by adding 6.5 µM NADH. After each NADH addition, a UV-Vis spectrum was recorded. Reduction was carried out until there was no further decrease in absorbance at 450 nm and/or until excess NADH accumulation was observed at 340 nm. Reduction of as-purified *Av*FixABCX was carried out in the similar way.

1.7. Electrochemistry

Protein thin film- square wave voltammetry (PTF-SWV) measurements were performed to assess the reduction potentials of FADs and [4Fe-4S] clusters in *Av*FixAB, *Av*FixX, *Av*FixCX and *Av*FixABCX. PTF-SWV measurements were taken using a Pyrolytic Graphite electrode (PGE, Pine Research, AFED050P040GE), which is the working electrode (WE) disk insert used on the ChangeDisk Rotating Disk electrode (RDE, AFE5TQ050PK) attached to the WaveVortex10 electrode rotator (Pine Research, AF01WV10). The Standard 250 ml cell kit features a center port (24/25) to accommodate the RDE shaft. Among the other five side necks (14/20), two are used to insert the Ag/AgCl (3M KCl) reference electrode (MF-2052) and platinum counter electrode (Pine Research, AFCTR5) through rubber septa. The other three ports are tightly closed using rubber septa. An electrochemistry experiment was performed on a benchtop while continuously sparging Ar through one of the ports of the electrochemical cell. First, the working electrode was cleaned using alumina suspension (90-187505) and silicon carbide paper (50-00075) as recommended by Pine Research. The sealed cell setup attached to all three electrodes was placed, with the working electrode facing upward, and purged with Ar for 10 min by piercing one of the septa to create an anaerobic environment. Then 5 μl of 30- 45 µM protein was placed through the septa on one of the ports using an airtight Hamilton syringe. Once the protein droplet was completely dry, the cell setup was mounted with the WE facing downward. After that, ~150 mL of degassed electrolyte buffer (50 mM Tris-HCl, 150 mM NaCl, 10% glycerol, 0.02% DDM, 1 mM DTT, pH 8.5) was loaded through the septa on the front neck while sparging with Ar until all three electrodes were fully immersed in the electrolyte. The same buffer at pH 8.0 was used without 0.02% DDM to assess the reduction potential of

[4Fe-4S] clusters in *Av*FixX. The electrodes were then connected to the CH Instrument Potentiostat, and a voltammogram was recorded using the CHI630E software. SWV data were collected with potential *E* applied in both the reducing and oxidizing directions, with time increments of 0.001 V, an amplitude of 0.025 V, a frequency of 10 Hz, and a sensitivity setting of 1×10^-4 $A \times V^{-1}$. *Av*FixABCX alone, *Av*FixABCX with $Av\text{Fld}_{NSQ}$, *Av*FixABCX/$Av\text{Fld}_{NSQ}$ mixture reduced with NADH, *Av*FixCX, and *Av*FixX with and without MBP tag were tested. Buffer background measurements were collected under identical conditions in the absence of the protein film. Each SWV measurement was repeated at least 3 times. All the *E* values are reported versus SHE, following conversion from the Ag/AgCl/ saturated KCl reference electrode by addition of 210 mV.

The thermodynamic and kinetic parameters resulting from these measured potentials were calculated using the Moser-Dutton formula (equation 1) where the electron transfer rate constant, k_{ET} in s^{-1}, ΔG° is the free energy in eV, R is the edge-to-edge distance between electron acceptor and donor in Å, β is a correction to the distance parameter that is proportional to barrier height equal to 1.1 $Å^{-1}$, γ is a nuclear term parameter equal to 3.1, and λ represents the reorganization energy of 0.7 eV[24].

$$\log k_{ET} = 13 - \left(\frac{\beta(R-3.6)}{2.303}\right) - \gamma\left(\frac{(G+\lambda)^2}{\lambda}\right) \quad \text{equation 1}$$

The equilibrium reduction potential, *E,* of the $NADH/NAD^+$ and UQ/UQH_2 redox couple was calculated using the Nernst equation (equation 2). Where E°, the standard reduction potentials are -320 mV and 200 mV, respectively. R is the universal gas constant in J mol^{-1} K^{-1}, T is the temperature in K, n is the number of electrons, and F is the Faraday constant, 96485 J $mol^{-}1$. [Red] and [Ox] indicate the concentrations of reduced and oxidized species used in the steady-state kinetic experiments.

$$E = E^{\circ} - \frac{RT}{nF} log \frac{[Red]}{[Ox]} \quad \text{equation 2}$$

The free energy change, ΔG° in eV was calculated using equation 3, where ΔE was calculated using *E* values measured in this study to use in the Moser-Dutton formula (equation 1); and to calculate reaction ΔG in kJ/mol, equation 3 was used, and ΔE was calculated using *E* values from equation 2.

$$\Delta G^{\circ} = -nF\Delta E \quad \text{equation 3}$$

1.8. Potentiometric Titration of *Av*FixABCX

A steady-state potentiometric titration was performed under strictly anaerobic conditions in a COY chamber at room temperature. The solution's reduction potential was monitored throughout the titration using an oxidation-reduction potential (ORP) electrode (Thermo Scientific 9678BNWP) connected to a pH meter (Orion Star A211). The ORP electrode was calibrated against a standard solution (Orion 967901, +222 mV vs SHE). The anaerobically purified *Av*FixABCX sample was concentrated and air-oxidized until a stable maximum absorption at 450 nm was observed. The air-oxidized *Av*FixABCX sample was concentrated to 72.9 µM in a 4 mL sample volume in a buffer containing 50 mM Tris, pH 8.5, 150 mM NaCl, 0.02% DDM, and 10% glycerol. A redox mediator cocktail was chosen based on the potential range (-580 mV to +220 mV) observed in PTF-SWV of *Av*FixABCX to establish an equilibrium electron transfer between the dye and cofactor of interest in the *Av*FixABCX sample. Mediator cocktail contained p-benzoquinone (E° = +220 mV), thionine (E° = -6 mV), Methylene blue (E° = +49 mV), Nile blue (E° = -146 mV), 2-hydroxy-1, 4 naphthoquinone (E° = -205 mV), Phenosafranin (E° = -312 mV),

Benzyl viologen (E°= -371 mV), and Methyl viologen (E°= -490 mV) was added to oxidized protein in 4 µM final concentration of each dye and measured the initial reduction potential prior to adding sodium dithionite (NaDT) reductant[25]. Then the protein was titrated toward more negative potentials by addition of 1 µL of NaDT either from 36 mM NaDT stock at the beginning or 66 mM stock at the end (NaDT stock was prepared inside the COY and concentration was determined at 315 nm, using ε = 8.043 mM^{-1} cm^{-1} in the working buffer; absorbance was measured in an airtight quartz cuvette)[25]. Following stabilization at a given potential, 200 µL samples were withdrawn, loaded into EPR tubes (Wilmad Lab Glass, 727-SQ-250M, 4mm OD), secured with a lid and Parafilm, and then flash-frozen in liquid nitrogen. Samples were withdrawn sequentially at -6, -75, -115, -142, -157, -179, -194, -214, -238, -285, -305, -328, -412, -445, -462, and -471 mV versus SHE.

1.9. EPR data collection for *Av*FixABCX, CuSO4 and Nernst fit

As purified FixABCX (69 µM) and as purified *Av*FixABCX treated with excess NaDT were prepared to test the EPR signal for the presence of a higher potential [4Fe-4S] cluster in *Av*FixABCX. *Av*FixABCX sample was treated with a stronger reducing agent, Ti(III) citrate, to ensure full reduction of all the cofactors. These samples were prepared without any dye cocktails. Ti(III) citrate solution at pH 8.5 in the above working buffer was prepared under strict anaerobic conditions in a Mbraun glove box by following the previously described protocol[26].

$CuSO_4$ standard solutions were prepared in the same buffer as the *Av*FixABCX samples. EPR spectra were recorded on an X-band Bruker ESP 300 spectrometer equipped with an Oxford Instruments ESR 900 liquid helium flow cryostat. Experimental conditions for the spectra of titration samples were as follows: microwave frequency ~9.37 GHz, temperature 10 K, microwave power 0.24 mW, modulation amplitude 13 G, time constant 160 ms, scan speed 10 G/s. Rhombic signals were simulated using EasySpin.

To assess the approximate reduction midpoint potential value for rhombic signal 1 and signal 2 (Figure 5B and 5C), the intensity of each low-field g-value (labeled in Figure 5C) in each signal was plotted against the measured reduction potential. The Nernst fit curve shown in Figure 5D was used to determine the reduction midpoint potentials of the [4Fe-4S] cluster for signals 1 and 2.

1.10. EPR radical quantification in *Av*FixABCX using $CuSO_4$ standard curve

Quantification of paramagnetic centers in *Av*FixABCX samples was performed by comparing their EPR signal intensities with a set of $CuSO_4$ standards prepared at concentrations ranging from 50 µM to 250 µM. The compared spectra were recorded under identical conditions at T = 10 K, using a lower microwave power of 3.9 µW for both *Av*FixABCX and CuSO4 to avoid saturation of the $CuSO_4$ sample.

1.11. Size Exclusion Chromatography coupled with Small Angle X-ray scattering (SEC-SAXS) data collection and processing.

Datasets were collected at the Cornell High Energy Synchrotron Source (CHESS) Biological Small Angle X-ray Scattering (BioSAXS) beamline, 7A1. All samples were prepared in a degassed buffer containing 50 mM Tris-HCl (pH 8.5), 150 mM NaCl, 5% glycerol, 0.02% DDM, and 1 mM DTT under anaerobic conditions in a COY chamber at the CHESS user facility. All samples were centrifuged at 14000 rpm for 10 min at 4 °C and stored anaerobically until the run. Size Exclusion Chromatography (SEC) separation was conducted at 4 °C using Superdex® 200 Increase 10/300 GL (Cytiva, 28-9909-44) under anaerobic conditions. Sequential 2-s exposures at a flow rate of 0.5 ml/min were used. *Av*FixABCX samples were prepared at a concentration of 56 µM (7.2 mg/mL). To observe conformational changes in *Av*FixABCX, 2

equivalents (eq) and 8 eq (excess) of NADH were added to as-purified and air-oxidized *Av*FixABCX. To test the conformational changes during electron bifurcation, *Av*FixABCX: *Av*Fld_{SQ}: UQ: NADH in the concentration ratio of 1:2:1:2 was added to the sample mixture. Likewise, to test the reversible reaction, *Av*FixABCX, *Av*Fld_{HQ}, UQH_2, and NAD^+ were mixed in the same ratio. A sample containing only *Av*FixABCX: UQ: NADH without *Av*Fld was tested by mixing the reactants in the same ratio to test any possible conformers that show domain II movement. Approximately 120 µL of the sample was loaded anaerobically into the SEC column using the sample pump as designed at the CHESS user facility. SAXS data were collected at 1.0937 Å at 4.89 × 10^{12} photons/sec. Data collection was followed as described before[27-29]. Data were collected in 1300 frames to ensure a sufficient scattering profile from the buffer background is present prior to the scattering of *Av*FixABCX particles, or at the end. SAXS data analysis was performed using RAW 2.2.2[30]. The original SEC-SAXS profile of each sample was subjected to liquid chromatography series analysis, with integral baseline correction using the buffer scattering region[31]. All the Figures S11B-S18 are generated in RAW.

The SEC-SAXS scattering profiles of all samples show three overlapping peaks, as shown in Figure S11B: two higher-intensity peaks and a lower-intensity peak in the as-purified, air-oxidized, and NADH-reduced *Av*FixABCX samples. However, when *Av*Fld is present, an additional lower-intensity peak elutes later in the profile due to excess unbound *Av*Fld. Most randomly distributed higher-molecular-weight particles (6680 kDa to 1000 kDa) elute in Peak 1; their radius of gyration (R_g) ranges from 180-60 Å, and the corresponding size exclusion chromatogram shown in Figure S11A indicates that these particles elute in the void volume of the SEC column (below ~9.5 mL), indicating polydispersity due to aggregation. Peak 2 contains particles with molecular weights of 1000 kDa to 260 kDa and R_g values ranging from 90-52 Å. A molecular weight of 260 kDa and an R_g of 52 Å suggest the presence of the *Av*FixABCX octamer. Therefore, some aggregated protein elutes in Peak 2 along with *Av*FixABCX. The component eluting in Peak 3 has an R_g of 30 Å. Based on the conditions tested, we expect an *Av*FixABCX octamer (260 kDa) in the as-purified, air-oxidized, and NADH-reduced *Av*FixABCX samples. In the bifurcation and confurcation reaction mixture, we expect an *Av*FixABCX-*Av*Fld complex (298 kDa). Moreover, the samples we tested could be highly dynamic in solution due to ongoing reactions in the presence of NADH, Q, and *Av*Fld. Therefore, we expect a mixture of conformers that is generated during the reaction.

Initially, we generated SAXS curves for frames with a constant R_g in peak 2. However, the SAXS curves of structural models derived from these experimental SAXS curves showed a higher χ^2 value in the low-q region in the Guinier fit. This discrepancy could be due to aggregation in peak 2. Therefore, to generate models that best fit the experimental SAXS curves, evolving factor analysis[32] (EFA) was performed on peak 2. First, the two overlapping adjacent peaks were included to confirm that peak 2 does not overlap with other peaks. However, this step suggested the presence of three components in peak 2. Then, the best-fitting region in peak 2 was selected by performing the EFA in each half of the peak by reducing the number of singular values to two. This approach isolated the best SAXS curve corresponding to the octamer of *Av*FixABCX. EFA of as-purified, air-oxidized and NADH-reduced samples (step by step analysis is shown in Figures S11-S14). Similarly, EFA for the samples containing all the components *Av*Fld_{NSQ}/Q/NADH in bifurcation reaction mixture and *Av*Fld_{HQ}/QH_2/NAD^+ in confurcation reaction mixture was performed and the step by step is shown in Figures S15-S17.

First, singular value decomposition (SVD) was performed to determine how many scattering components are present in the entire elution profile, in the three overlapping peaks, and in peak 2[32]. SVD shows seven scattering components in the entire profile and six in the three overlapping peaks, but only 3 components in peak 2 (Figures S11, S12, and S15). SVD of peak 1 alone shows four components, suggesting that most components in the elution profiles are aggregated proteins. Therefore, to better resolve the higher-intensity peak 2 of interest from the overlapping peaks, evolving factor analysis (EFA) was performed on

the three overlapping peaks[32]. EFA helps separate components within overlapping peaks. Peak 1 contains varying degrees of aggregation, with R_g ranging from 180-60 Å, whereas in peak 2, R_g ranges from 90-50 Å, covering the expected R_g value of ~52 Å. Therefore, to avoid the complexity of deconvoluting peak 2 from peak 1, frames that contain particles above 90 Å in peak 1 were excluded (Figure S13 and S16).

SVD of this region shows four singular values, and EFA isolates peak 2 from both overlapping peaks, as shown in Figure S13. This EFA shows three components in Peak 2. As mentioned above, SVD of Peak 2 alone showed three components. Therefore, excluding frames with higher-MW aggregates did not introduce discrepancies. Additionally, components from Peak 1 stop eluting in Peak 2, while components in Peak 3 start eluting in Peak 2. However, in Peak 2, the higher-concentration components resemble the SAXS curve of the *Av*FixABCX octamer, and these components do not overlap with adjacent peaks 1 and 2.

After ensuring the frames where peak 2 starts to elute and ends eluting, and to reduce the number of singular values and make the absolute values of the left and right singular vectors as equal as possible, the best frames for peak 2 were picked for EFA. In the as-purified *Av*FixABCX sample, the step-by-step EFA analysis was shown in Figures S13 and S14. Likewise, in air-oxidized and NADH-reduced *Av*FixABCX samples, both the first and second halves of peak 2 were subjected to EFA to ensure the best region that gives the SAXS curve of the *Av*FixABCX octamer. This approach helped us simplify the EFA by selecting two components instead of three. Moreover, as shown in Figure S13, the other two components, besides the octamer, are at much lower concentrations. One appears in the 1st half while the other in the 2nd half. Overall, EFA analysis of each sample helped gather the scattering profiles of the higher-concentration components. As shown in Figure S18A, the SAXS curves generated from this step-by-step EFA resemble the SAXS curve of the octamer of *Av*FixABCX.

Some scattering profiles obtained from EFA analysis did not have a constant R_g value due to aggregates; therefore, the profile with the best Guinier fit and R_g values was chosen and subjected to molecular weight (MW) analysis. Scattering profiles derived from EFA analysis and based on the constant R_g value in peak 2 are very similar, as shown in Figures S14H and S17H. Each SAXS profile generated using the EFA analysis was subjected to Guinier analysis to assess profile quality by fitting a linear function to the experimental data. Molecular weight (MW) was determined using the volume of correlation (Vc) method.

The air-oxidized sample treated with 2eq of NADH and the as-purified *Av*FixABCX treated with Q and NADH showed the presence of octamer and tetramer in the samples, as determined from the MW obtained from the volume of correlation (Vc). However, all the other samples showed the presence of the octamer of *Av*FixABCX or *Av*FixABCX-*Av*Fld (if Fld is present in the sample). Dimensionless Kratky plots of the tetramer show a partially folded protein, possibly due to instability in solution prior to dimerization. Therefore, we subjected the octamer SAXS curves to further analysis. The MW of as-purified *Av*FixABCX was 243 kDa, while Fld_{NSQ}-bound *Av*FixABCX showed 260 kDa, indicating that a 17 kDa Fld has bound to *Av*FixABCX (Fld binds to *Av*FixABCX only in its NSQ state, as shown in Figure S6A). We validated the SAXS profiles generated from the Guinier fit using GNOM analysis (Table S7). Then, the GNOM analysis from the ATSAS data analysis package was used to fit the data, calculate a P(r) curve, and estimate the D_{max} and R_g[33]. We selected the final experimental scattering curves for the octamer in the samples based on the best Guinier fit and GNOM quality estimates. All SAXS scattering plots showed a good fit to the calculated Pair-distance distribution function (P(r)), with a total estimate (TE) ranging from 0.65 to 0.75 and a χ^2 value between 1 and 2 in all samples (all profiles are shown in Figure S18A). The dimensionless Kratky plot for all samples indicates a fully folded globular protein with two peaks (Figures S19A and S19B). To compare the P(r) curve of samples with the cryo-EM structure of the *Av*FixABCX octamer, SAXS scattering plots for PDB files were generated using CRYSOL from the ASTAS package. P(r) and log-lin plots reveal two curves for the octamer and one for

the tetramer (see Supporting Information Figure S18A). The MW determined for the octamer was consistent with the MW of the cryo-EM structure (Table S7). The P(r) of all the tested samples showed a higher D_{max} above 139 Å than the cryo-EM structure of *Av*FixABCX, indicating the presence of extended particle size in the solution state (Figure S19A and Table S7).

GNOM output files (.out) were used as inputs to calculate DENSS maps for the octamer in the samples using DENSS in the RAW interface. In slow mode, an average of 20-50 reconstructions were run by aligning the outputs to the *Av*FixABCX cryo-EM structure[34]. DENSS maps were generated for SAXS profiles of the cryo-EM structure and all the octamers. Although the resolution is lower than that of the cryo-EM map, the DENSS maps clearly show extended particles with orientations distinct from the cryo-EM structure (see Supporting Information Figure S20A). The scattering pattern is isotropic because particles in solution have random orientations. Moreover, due to the conformational flexibility, the scattering pattern is polydisperse; therefore, experimental information such as R_g and shape may correspond not to a single particle but to an average over the entire ensemble[35]. Hence, a minimum ensemble search (MES) using BilboMD was performed to resolve the structural variability in the scattering pattern[36]. BilboMD is a rigid-body modeling strategy in which MD simulations are performed on known, higher-resolution structures by defining domain connections at very high temperatures. The MD simulation provides an ensemble of molecular models from which a SAXS curve is generated and compared to the experimentally determined SAXS profile[36]. To generate 3D domain structures of the octamer based on conformational sampling using the molecular dynamics (MD) approach, BilboMD classic with the PDB server was used[36]. Conformational sampling was performed using CHARMM; the structure was validated by FoXS, and a minimal ensemble search was conducted using MultiFoX[37-40]. The model, Fld_{NSQ} bound to *the Av*FixABCX octamer (Fix/Fld) in a 1:1 ratio, was generated using ClusPro, and the crystal structure of *Azotobacter vinelandii* Flavodoxin II (PDB 5k9B) and the cryo-EM structure of FixABCX were used[41-46]. The .inp files were generated in BilboMD by providing a PDB file of the *Av*FixABCX octamer and defining the rigid and flexible regions in the *Av*FixABCX subunits. Residues 250-279 in FixA and residues 235-359 in FixB were defined as flexible regions in domain II of FixAB. The same residues were used as the flexible residues to generate Fix/Fld .inp files. To determine the conformers in SEC-SAXS samples that were treated with Fld_{NSQ} or Fld_{HQ}, the PDB file of the Fix/Fld model was used, and for other samples, the PDB file of the cryo-EM structure was used (PDB 9ZQZ).

We selected the ensemble of models with the lowest χ^2 (1-2) as the best-fitting model. As shown in Table S8 and Figure S20D and S20E, SAXS scattering plots of the generated models fit the experimental SAXS scattering plots; and in Figure S20, panels B and C, the DENSS map of extended particles generated in RAW fits the *Av*FixABCX-*Av*Fld model representing an extended conformer. We used the populations (as tabulated in Table S8) of the best-fitting model in each tested sample to interpret the possible EB mechanism of *Av*FixABCX in vitro. PDB of the *Av*FixABCX cryo-EM structure was used to analyze SAXS curves of air-oxidized, as-purified samples, and reduced samples. Similarly, the *Av*FixABCX-*Av*Fld model was used to generate conformers in the bifurcation and conformation reaction mixture. In all analyses, flexible residues in the FixA and FixB chains that anchor domain II were defined, since domain II motion is expected during electron transfer and has been reported in solution-state and higher-resolution structures of Etf[47-51]. All SAXS experimental data, along with the relevant experimental conditions, sample details, derived models, and their fits, were deposited in the SASBDB database (http://www.sasbdb.org/).

1.12. Steady State Kinetics

Steady-state kinetic experiments were carried out under anaerobic conditions in an airtight, screw-capped, self-masking quartz cuvette (Starna spectrophotometer cells, Cat. no. 18B-Q-10-GL14-S). All the samples were prepared in or buffer-exchanged into 50 mM Tris (pH 8.5), 150 mM NaCl, 0.02% DDM, and 10%

glycerol. Anaerobically purified and stored *Av*FixABCX and *Av*Fld samples were thawed inside the COY chamber prior to starting the reaction. All assays were carried out in 50 mM Tris, pH 8.5, 150 mM NaCl, 0.02% DDM, and 10% glycerol. The buffer was sparged with N_2 before transfer into the COY chamber and then supplemented with DDM; DTT was not added, as it tends to reduce UQ and *Av*FixABCX. As a higher-potential electron acceptor, Ubiquinone-5 (UQ, referred to as Q throughout the main text) was used and is a coenzyme Q1 derivative (Enzo cat. no. ALX-270-294). UQ stock was prepared by dissolving 2 mg in 1 mL of 50% ethanol. The absorbance spectrum of 300 µM oxidized UQ was recorded, then fully reduced to UQH_2 with NaDT to determine ε_{280} and ε_{290}. *Av*Fld was used as a low-potential electron acceptor; its expression is encoded by the *niff* gene and is referred to as flavodoxin II in many publications[46, 52]. *Av*Fld binds to *Av*FixABCX only in the semiquinone state (as shown in Figure S6A); therefore, *Av*Fld was expressed and purified as described before in the neutral semiquinone state (Fld_{NSQ})[53]. The absorbance spectrum of Fld_{NSQ} was recorded and FMN content was determined using ε_{445} = 12.5 mM^{-1} cm^{-1} [2]. *Av*Fld was in the 92% semiquinone state, and FMN was fully occupied in Fld_{NSQ}. The following reactant concentrations were used, depending on the reactions monitored: 1 µM *Av*FixABCX, 85 µM *Av*Fld_{NSQ}/*Av*Fld_{HQ}, 200 µM NADH/NAD^+, and 300 µM UQ/UQH_2. During the redox reaction, the changes in the absorbance spectrum were monitored using the Agilent Technologies Cary 6000i UV-Vis-NIR spectrophotometer. After recording the baseline buffer spectrum, NADH, UQ, and Fld_{NSQ} were added sequentially to their respective final concentrations, and the spectrum was recorded again. Then FixABCX was added to a total reaction mixture of 400 µl. The bifurcation reaction was monitored by recording the spectrum from 200- 800 nm every 30 sec for 10- 20 min. To monitor the electron confurcation, *Av*FixABCX, UQ, and Fld_{NSQ} were fully reduced using NaDT, and the initial spectrum was recorded. Then, NAD^+ was added, and the absorption spectrum was monitored. Similarly, a higher-potential half-reaction without Fld_{NSQ}/Fld_{HQ} and a lower-potential half-reaction in the absence of UQ/UQH_2 were carried out by adding NADH for the forward reaction and NAD^+ for the reverse reaction. NADH formation or oxidation, Fld_{NSQ} formation or reduction were monitored at 340 nm and 580 nm, respectively, and extinction coefficient (ε_{340} = 6.22 mM^{-1} cm^{-1}, ε_{580} = 5.7 mM^{-1} cm^{-1})[46, 52] values were used to measure the concentrations. Moles of NADH formed in the higher-potential reverse half-reaction were determined using the total volume of the reaction and the concentrations of NADH formed. This value was then divided by the milligrams of protein used (0.05 mg) and the time difference in minutes (~20 min). Similarly, moles of Fld_{NSQ} formation were calculated for electron bifurcation, electron confurcation, and the low-potential half-reaction in the reverse direction.

1.13. Spectral deconvolution

Individual spectra of known concentrations of *Av*Fld_{NSQ}, NADH, oxidized UQ, and reduced UQH_2 were recorded using an Agilent Technologies Cary 6000i UV-Vis-NIR spectrophotometer. The higher-potential half-reaction in the forward direction is the fastest of all. To determine the reaction stoichiometry of the higher-potential branch in the forward direction, we calculated the concentrations of NADH and UQ from absorbance decrease at 340 nm and 280 nm, respectively. Similarly, for a reversible reaction, we subtracted the initial spectrum of the reaction mixture from the final spectrum. This will provide the spectrum of the species undergoing the reaction. The absorbance increase at 340 nm was used to determine the concentration of NADH formed; then the NADH spectrum corresponding to this concentration was subtracted from the final spectrum to obtain the UQ spectrum and calculate the concentration. The stoichiometry of Fld_{NSQ} to NADH was determined using a lower-potential reversible reaction. The concentration of Fld_{NSQ} formed was determined using absorbance at 580 nm. The Fld_{NSQ} spectrum at this concentration was subtracted to obtain the NADH spectrum, and the concentration was determined from the absorbance at 340 nm. Similarly, the stoichiometry of *Av*Fld_{NSQ} to NADH to UQ was assessed using the confurcation reaction, as it had a significant spectral change compared to the bifurcation reaction. The initial spectrum was subtracted from the final spectrum to eliminate any hydroquinone spectral contribution. The concentration of *Av*Fld_{NSQ} formed was calculated at 580 nm, and

the spectrum of *Av*Fld_{NSQ} corresponding to this concentration was subtracted to obtain the NADH+UQ spectrum. Then, absorbance at 340 nm was used to calculate the NADH concentration, as UQ has minimal spectral contribution at 340 nm, and the corresponding NADH spectrum was subtracted to obtain the UQ spectrum. Absorbance at 280 nm was used to calculate the concentration of UQ (ε_{340} = 1.5 mM^{-1} cm^{-1}).

1.14. Crosslinking Mass spectrometry (XL-MS)

Protein-protein interactions within the *Av*FixABCX complex were investigated as described previousl[1]. Briefly, chemical cross-linking was performed using 20 µg of the *Av*FixABCX complex and 1 mM bis(sulfosuccinimidyl)suberate (BS3) (Thermo-Fisher, Waltham, MA) in 50mM HEPES, pH 7.2, 150mM NaCl buffer at room temperature for 1 h. The reaction was quenched by adding 120 mM Tris (final concentration). The resulting mixture was separated by SDS-PAGE (4-20% linear gradient gel, Bio-Rad, Hercules, CA) and stained with Coomassie Brilliant Blue (Thermo Fisher Scientific, Waltham, MA). Protein bands of interest were excised from the gel and digested with trypsin at 37°C overnight. Resulting peptides were analyzed using a maXis Impact UHR-QTOF instrument (Bruker Daltonics, Billerica, MA) interfaced with a Dionex 3000 nano-uHPLC (Thermo-Fisher, Waltham, MA) as described befor[53]. Cross-links were identified in the Spectrum Identification Machine. Precursor and fragment ion tolerances were set to ±20ppm.

1.15. Transient absorption spectroscopy (TAS)

The TAS experiment was performed as described previously[54]. For all transient absorption measurements, the sample was made in an MBraun glove box (N_2 atmosphere), sealed in a 2 mm quartz cuvette, and constantly stirred to prevent photodegradation. *Av*FixABCX and *Av*FixABCX/*Av*Fld_{NSQ} mixture concentrations were approximately ~60 µM. As purified *Av*FixABCX is in a partially reduced state, light can be used to initiate the formation of SQ species in oxidized FAD in the following way: photons of sufficient energy are used to excite FAD OX to its excited state (FAD*). Owing to the highly oxidizing nature of FAD*, an electron is abstracted from a nearby donor to form the mechanistically relevant one-electron reduced SQ species that relaxes by electron transfer to reform FAD OX. All experiments were conducted at room temperature. The change in absorbance signal (ΔA) was calculated from the intensities of sequential probe pulses with and without the pump pulse excitation. The data collection (350 pump shots per time point) was carried out four consecutive times and then averaged. The experiment was repeated two times. Kinetic traces were fit in Igor Pro 9 software using the least number of components required using the following equation:

$$\Delta Abs.\ (mA.U.) = y_0 + A_1 * e^{\left(\frac{-(x-x0)}{\tau_1}\right)} + A_2 * e^{\left(\frac{-(x-x0)}{\tau_2}\right)} + A_3 * e^{\left(\frac{-(x-x0)}{\tau_3}\right)}.$$

1. Results:

Table S1: The list of primers used to amplify the *Av*FixABCX and *Av*FixAB genes.

Construct	Forward	Reverse

FixA	CGCGGATCCGCACAGCGTCGTCTGTATC AAGCAGGTGCC	CCCAAGCTTTCAGACCGCCCGCTT GGCGATTTCCTGTT
FixB	GGAATTCCATATGAGCGAACAACCGAAAAAGCC GACGAAGAAGAAGGTCG	AATGGTACCTCAGCCGGCTTTTTTCAG TTGTCCCAGACGGG
FixABCX	CCGGAATTCATGCACAGCGTCGTCTGTATCAAG CAGGTGCC	CGCGGATCCTCAGTTCTTGGCCGAGCG GCTCCGCCA

Table S2: cryo-EM data collection, processing, model refinement and validation.

	**EMD-74585 PDB 9ZQZ**
**Data collection**	
Microscope	Talos Arctica
Voltage (kV)	200
Detector	K3 (Counting)
Magnification (nominal/calibrated)	×45,000/×55,187
Exposure navigation	Image shift to 25 holes
Electron exposure (e^-/Å^2)	56 e^- Å^2
Data acquisition software	SmartScope/SerialEM
Total electron exposure (e^-/Å^2)	65
Exposure rate (e^-/pixel/sec)	14.9
Frame length (ms)	70
Number of frames per micrograph	50
Pixel size (Å)	0.9061
Defocus range (μm)	-0.8 to -2.4
Micrographs collected	3436
**Reconstruction**	
Image processing package	cryoSPARC
Total extracted particles (blob picks)	1,593,178
Final number particles	67,249
Symmetry imposed	C2
Resolution (Å)	
FSC 0.143 (masked/unmasked)	4.72/5.4
**Model Composition (#)**	
Chains	8
Atoms	17,618
Hydrogen	0
Residues	Protein: 2236
Water	0
Ligands	FAD: 6<br>SF4: 4
**Model Refinement**	
Refinement Package	Phenix
Bonds (RMSD)	
Length (Å) (# > 4σ)	0.014 (0)
Angles (°) (# > 4σ)	2.073 (0)
**Model Validation**	
MolProbity score	1.44
Clash score	5.83
Ramachandran (%)	
Outliers	0.00

Allowed	2.61
Favored	97.39
Rama-Z	
whole (N = 2248)	1.32 (0.17)
helix (N = 782)	1.88 (0.18)
sheet (N = 456)	1.24 (0.22)
loop (N = 1010)	0.06 (0.19)
Outliers	
Rotamer outliers (%)	0.00
Cβ outliers (%)	0.00
Peptide Plane, Cis Pro/general (%)	5.7/0.0
Peptide Plane, Twisted Pro/general (%)	0.0/0.1
CaBLAM outliers (%)	1.54
ADP (B-factors)	
Iso/Aniso (#)	17618/0
min/max/mean	
Protein	0/206/84
Ligand	1/228/51
Occupancy	
Mean	1.00
occ = 1 (%)	100.00
0 < occ < 1 (%)	0.00
occ > 1 (%)	0.00
**Model vs. Data**	
Resolution (Å)	Unmasked Masked
d FSC model (0.5)	5.0 5.2
CC (mask)	0.72
CC (box)	0.57
CC (peaks)	0.44
CC (volume)	0.68
Mean CC for ligands	0.75
Iso/Aniso (#)	17618/0
min/max/mean	
Protein	287/1013/507
Ligand	328/804/501
Occupancy	
Mean	1.00
occ = 1 (%)	100.00
0 < occ < 1 (%)	0.00
occ > 1 (%)	0.00
**Model vs. Data**	
Resolution (Å)	Unmasked Masked
d FSC model (0.5)	6.8 6.5
CC (mask)	0.69
CC (box)	0.89
CC (peaks)	0.65
CC (volume)	0.68
Mean CC for ligands	0.75

Table S3: Moser-Dutton equation parameters and rates of the reaction

Donor	Acceptor	Distance/ Å	Donor	Acceptor	Rate (k_{ET})

			E, mV	E, mV	s^{-1}
Bf-$FAD_{OX/SQ}$	$Fld_{NSQ/HQ}$	10	-673	-460	10^9
Bf-$FAD_{OX/SQ}$	$Fld_{NSQ/HQ}$	8.5*	-673	-460	4 x 10^9 *
Bf-$FAD_{OX/SQ}$	ET-$FAD_{SQ/HQ}$	25	-673	+20	10^3 †
Bf-$FAD_{SQ/HQ}$	ET-$FAD_{SQ/HQ}$	25	+153	+20	0.5
Bf-$FAD_{SQ/HQ}$	ET-$FAD_{SQ/HQ}$	18	+153	+20	10^3
Bf-$FAD_{OX/SQ}$	ET-$FAD_{SQ/HQ}$	18	-673	+20	10^6
Bf-$FAD_{SQ/HQ}$	ET-$FAD_{SQ/HQ}$	14	+153	+20	10^5 ‡
ET-$FAD_{SQ/HQ}$	$[4Fe\text{-}4S]_{II}$	10	+20	-131	10^7
$[4Fe\text{-}4S]_{II}$	QR-$FAD_{OX/NSQ}$	12	+50	-131	10^8

The distance between donor and acceptor is obtained from the cryo-EM structure, and the measured *E* values are used.

‡ When considering domain II movement that brings ET-FAD as close as 14 Å to receive a single electron from Bf-$FAD_{SQ/HQ}$, assuming measured *E* values were the same as when 25 Å apart.

* Rate was calculated using the Bf-FAD SQ lifetime (250 ps) to recalculate the distance between Bf-FAD and Fld.

†Rate of electron transfer from Bf-$FAD_{OX/SQ}$ to $Fld_{NSQ/HQ}$.

Table S4: Calculated non-equilibrium reduction midpoint potentials.

Redox couple	E^{o}, mV	E, mV	No of electrons	Ref.*
$NADH/NAD^+$	-320	-480	2	[55]
Q/QH_2	+200	+260	2	[56]
$NAD^+/NADH$	-320	-380	2	[55]
QH_2/Q	-400	-460	2	[2]
Fld_{NSQ}/Fld_{HQ}	-460	-340	1	This study
Fld_{HQ}/Fld_{NSQ}	-460	-580	1	This study

***** References for the standard reduction midpoint potentials

Table S5: Activity assay of each individual reactions in the forward, reverse direction, confurcation and bifurcation reactions, stoichiometric coefficient between the reactants and free energy change of each reaction.

Reaction	Products	Specific Activity nmol/mg/min	Stoichiometry	Turnover number/ s^{-1}	ΔG kJ/mol
NADH → Q	NAD^+, QH_2	Fastest	1:1	NM*	-42.4
QH_2 → NAD^+	Q , NADH	2.51± 0.04 (n=3)	1:1	5 × 10^3	-15.4
NADH → Fld_{NSQ}	NAD^+, Fld_{HQ}	0.045 (n=3)	NM*	92	-27.0
Fld_{HQ} → NAD^+	NADH, Fld_{NSQ}	7.7 (n=3)	1:1	1.6 × 10^4	-38.6
NADH → Fld_{NSQ} + Q	QH_2 , Fld_{HQ}, NAD^+	0.87 ± 0.13 (n=3)	Fld_{NSQ} : NADH: Q 2:2:1	1.8 × 10^3	-69.4
QH_2 + Fld_{HQ} → NAD^+	NADH , Fld_{NSQ} , Q	7.4 ± 0.4 (n=3)	Fld_{HQ} : NAD^+: QH_2 2:2:1	1.5 × 10^4	-54.0

Note: free energy change ΔG in kJ/mol is calculated using ΔG = -nFΔE, where n is the number of electrons, F is the Faraday's constant and ΔE is the difference between the reduction midpoint potential of electron acceptor and donor which were the calculated non-equilibrium reduction midpoint potentials tabulated in the Table S4 for each reaction.
NM* indicates not measurable.

Table S6: Fitting parameters for the data shown in Figure S8.

	FixABCX alone		***FixABCX + Fld***	
Wavelength (nm)	***366 (ASQ)***	***625 (NSQ)***	***366 (ASQ)***	***625 (NSQ)***
y_0 (mA.U.)	-2 ± 7	0.18 ± 0.04	0 ± 2	-0.4 ± 0.1
x_0 (ps)	0.92	0.92	0.92	0.92
A_1 (mA.U.)	-2.9 ± 0.5	1.83 ± 0.05	-2 ± 1	1.9 ± 0.1
t_1 (ps)	1.8 ± 0.4	210 ± 19	2 ± 1	180 ± 40
A_2 (mA.U.)	1.2 ± 0.3	-	1.4 ± 0.4	-
t_2 (ps)	260 ± 100	-	140 ± 83	-
A_3 (mA.U.)	5 ± 7	-	2.5 ± 1	-
t_3 (ps)	*> 5000*	-	*> 5000*	-
χ^2 (•10^{-5})	1.70	1.36	9.17	8.21
	-	-	9.27*	8.23*

*****_When locked to parameters from corresponding FixABCX alone trace_

Table S7: Primary analysis of SAXS profiles using RAW and GNOM from ATSAS package

FixABCX Sample	Conditions	Guinier R_g, Å	GNOM R_g, Å	D_{max}, Å	Mw kDa, (exp, 257)* Vc
FixABCX Octamer	Cryo-EM	49.5	49.4	139	223*
As purified	No additive	53.0	52.0	157	243
	2 eq NADH	53.0	53.0	160	239
	8 eq NADH	53.0	53.0	160	231
Air Oxidized	No additive	53.0	52.0	155	267
	2 eq NADH	52.0	52.0	155	256
	8 eq NADH	49.0	51.0	155	235
As purified	1 eq UQ, 2 eq NADH	52.0	52.0	156	223
As purified	1 eq UQ, 2 eq NADH, 2 eq Fld_{NSQ}	55.0	54.0	160	260
Reduced FixABCX with NaDT	1 eq UQH_2, 2 eq NAD^+, 2 eq Fld_{HQ}	53.0	53.0	160	229

* Molecular weight of *Av*FixABCX based on the mass spectrometric analysis, 257 kDa
* SAXS curve generated for cryo-EM structure was ran on RAW to determine the MW using the Vc, which gave 223 kDa, this is to compare the SAXS profile of higher resolution cryo-EM map with low resolution SAXS data.

Table S8: Selected ensembles from BilboMD and populations of models.

1)**Air oxidized FixABCX**				**2) As purified FixABCX**			
ensemble no.	χ^2	population (%)	R_g (Å)	ensemble no.	χ^2	population (%)	R_g(Å)
4	1.4	60	45	5	1.74	17	45
		16	45			9	52
		10	66			20	59
		16	66			29	45
						24	80
**3) Oxidized+2eq NADH**				**4) As purified +2eq NADH**			
ensemble no.	χ^2	population (%)	R_g (Å)	ensemble	χ^2	population (%)	R_g(Å)
4	1.41	45	58	2	1.47	58	45
		24	44			42	66
		18	44				
		14	72				

5) Oxidized + 8eq NADH

ensemble no.	χ^2	population (%)	R_g (Å)
3	1.33	82	42
		5	48
		13	60

6) As purified + 8eq NADH

ensemble	χ^2	population (%)	R_g(Å)
3	1.37	48	45
		37	55
		15	73

7) As purified + 2eq NADH + 1eq UQ + 2 eq Fld_{NSQ}

ensemble no.	χ^2	population (%)	R_g (Å)
1	1.77	100	74

8) NaDT reduced FixABCX + 2eq NAD^+ + 1eq UQH_2 + 2 eq Fld_{HQ}

ensemble	χ^2	population (%)	R_g(Å)
1	1.91	100	66

9) As purified + 2eq NADH + 1eq UQ

ensemble no.	χ^2	population (%)	R_g (Å)
1	1.33	28	51
		50	44
		22	72

Table S9: Protein-protein interactions captured with BS3 within FixABCX complex.

	Experimental M+H Primary Score		ppm	Peaks Matched	Peaks Matched	Peptide Sequence (additional mass modifications indicated)	Protein 1 Protein 2		Cross-linked residue	Cross-linked residue	Cα to Cα Distan
A with B				Protein 1	Protein 2				Protein 1	Protein 2	
	4839.566	10.77	8.1	86	20	DYRNEPYTKALTDLVNSYQPEILMLGATTLGR - KEIQVERR	FIXB	FIXA	103	158	25.4
	5727.943	7.62	4.4	44	31	GKEICGEAFQHGADKAYLLQHEILRDYR - VAAGIDTVEMIGLKGSPTVVS(156.0786)K	FIXB	FIXA	84	227	30.8
	4185.177	7.82	5.5	62	15	HVWVC(57.02146)IESERGVVHPVSWELLGEGRK - IVDVDTAKK	FIXB	FIXA	31	157	33.9
	5727.943	7.62	4.4	44	31	GKEICGEAFQHGADKAYLLQHEILRDYR - VAAGIDTVEMIGLKGSPTVVS(156.0786)K	FIXB	FIXA	84	225	34.8
	6080.245	7.73	11.1	35	41	HVWVC(57.02146)IESERGVVHPVSWELLGEGRK - VWDRVAAGIDTVEMIGLKGSPTVVS(156.0786)K	FIXB	FIXA	31	225	35.8
	5727.939	12.75	5.1	61	65	GKEICGEAFQHGADKAYLLQHEILRDYR - VAAGIDTVEMIGLKGSPTVVS(156.0786)K	FIXB	FIXA	71	227	37.2
	5969.066	12.13	10.5	62	58	HVWVC(57.02146)IESERGVVHPVSWELLGEGRK - LK(156.0786)DKFGGTVTVVTMGPPMAEAALRK	FIXB	FIXA	31	51	37.4
	5969.066	12.83	10.5	62	65	HVWVC(57.02146)IESERGVVHPVSWELLGEGRK - LKDK(156.0786)FGGTVTVVTMGPPMAEAALRK	FIXB	FIXA	31	49	39.1
	4186.197	8.51	13.7	60	24	NLASTRPTFGGSLLC(57.02146)TILTQR - IVDVDTAKKEIQVER	FIXB	FIXA	166	157	42.1
	5727.939	12.65	5.1	61	64	GKEICGEAFQHGADKAYLLQHEILRDYR - VAAGIDTVEMIGLKGSPTVVS(156.0786)K	FIXB	FIXA	71	225	42.2
	6949.570	14.36	8.2	79	63	FGGTVTVVTMGPPMAEAALRKC(57.02146)LSFGADDAILVSDR - HVWVC(57.02146)IESERGVVHPVSWELLGEGRK	FIXA	FIXB	72	31	42.9
	4034.089	9.55	6.2	58	36	KLADALGVELYGVVICGPGER - KIMEDMPVDLIFTGK	FIXB	FIXA	49	107	46.5
A with C											
	6080.255	9.52	8.9	52	42	ESITLGVGCMLSDFKQQKIS(156.0786)PYELLDR - QGVPAIINPYDLFALEEALRLKDK	FIXC	FIXA	246	49	13.5
	4112.079	7.03	14.9	58	11	ESITLGVGCMLSDFKQQKISPYELLDR - KEIQVER	FIXC	FIXA	243	158	20.9
	7025.418	15.05	19.0	74	75	KC(57.02146)LSFGADDAILVSDRAFAGSDTLATSYALSAVIR - ESITLGVGCMLSDFKQQKIS(156.0786)PYELLDR	FIXA	FIXC	72	246	21.5
	4094.064	7.72	11.1	47	29	DKFGGTVTVVTMGPPMAEAALR - THPS(156.0786)IAPLIAGSEMK	FIXA	FIXC	51	272	24.4
	4034.089	9.05	9.6	43	46	MHSVVCIKQVPDSAQIR - ESITLGVGCMLSDFKQQK	FIXA	FIXC	1	246	25.9
	4112.079	7.64	14.9	64	11	ESITLGVGCMLSDFKQQKISPYELLDR - KEIQVER	FIXC	FIXA	251	158	26.1
	5727.934	10.04	5.3	74	25	DKFGGTVTVVTMGPPMAEAALRKC(57.02146)LSFGADDAILVS(156.0786)DR - KLDDSFVMKDLK	FIXA	FIXC	75	351	26.1
	6080.245	8.11	7.3	49	31	ESITLGVGCMLSDFKQQKIS(156.0786)PYELLDR - QGVPAIINPYDLFALEEALRLKDK	FIXC	FIXA	249	49	26.3
	5727.934	10.66	5.3	80	25	DK(156.0786)FGGTVTVVTMGPPMAEAALRKC(57.02146)LSFGADDAILVSDR - KLDDSFVMKDLK	FIXA	FIXC	72	351	27.1
	4112.079	7.13	14.9	59	11	ESITLGVGCMLSDFKQQKISPYELLDR - KEIQVER	FIXC	FIXA	246	158	27.4
	4029.078	7.34	11.9	41	31	AADK(156.0786)PFSAANLAEYKK(156.0786)K - QTIDGDTAQVGPGIAKR	FIXC	FIXA	349	137	27.8
	4839.566	11.05	0.1	67	42	LKDK(156.0786)FGGTVTVVTMGPPMAEAALRK - QGGEVRADAVILADGVNSR	FIXA	FIXC	49	165	28.0
	7025.418	14.85	19.0	66	81	KC(57.02146)LSFGADDAILVSDRAFAGSDTLATSYALSAVIR - ESITLGVGCMLSDFKQQKIS(156.0786)PYELLDR	FIXA	FIXC	75	249	30.0
	5727.934	10.05	8.9	68	31	HELKVWDRVAAGIDTVEMIGLKGSPTVVS(156.0786)K - ES(156.0786)ITLGVGC(57.02146)MLSDFKQQK	FIXA	FIXC	207	243	30.2
	7025.418	15.66	19.0	74	81	KC(57.02146)LSFGADDAILVSDRAFAGSDTLATSYALSAVIR - ESITLGVGCMLSDFKQQKIS(156.0786)PYELLDR	FIXA	FIXC	72	249	30.9
	4094.064	7.21	2.4	35	36	AFAGSDTLATSYALSAVIR - ESITLGVGC(57.02146)MLSDFKQQK	FIXA	FIXC	98	246	31.0
	5471.799	7.31	0.8	53	19	QQKISPYELLDRMKTHPS(156.0786)IAPLIAGS(156.0786)EMK - CLSFGADDAILVS(156.0786)DR	FIXC	FIXA	260	75	31.2
	4839.566	12.08	13.6	101	18	EAGVLTICETTVTDLLIEGGKVVGVRTDRQGGEVR - VFAPKPRSK	FIXC	FIXA	139	241	31.2
	6949.570	14.65	2.2	89	56	FGGTVTVVTMGPPMAEAALRKC(57.02146)LSFGADDAILVS(156.0786)DR - GETPGSKNVQGAILYADAIEKIIPDFR	FIXA	FIXC	72	41	31.4
	4190.183	8.41	2.6	58	25	SKRAELIESHDS(156.0786)DPKNLAEAALAK - ADAVILADGVNSR	FIXA	FIXC	242	165	31.8
	4112.079	7.74	14.9	65	11	ESITLGVGCMLSDFKQQKISPYELLDR - KEIQVER	FIXC	FIXA	249	158	31.9
	4094.064	7.21	2.4	35	36	AFAGSDTLATSYALSAVIR - ESITLGVGC(57.02146)MLSDFKQQK	FIXA	FIXC	102	246	32.3
	6949.570	14.35	2.2	87	55	FGGTVTVVTMGPPMAEAALRKC(57.02146)LSFGADDAILVS(156.0786)DR - GETPGSKNVQGAILYADAIEKIIPDFR	FIXA	FIXC	75	42	33.1
	4094.064	7.13	12.7	58	12	AADKPFSAANLAEYKK(156.0786)KLDDSFVMK - QVPDSAQIR	FIXC	FIXA	349	13	33.6
	5727.934	9.85	8.9	68	29	HELKVWDRVAAGIDTVEMIGLKGSPTVVS(156.0786)K - ES(156.0786)ITLGVGC(57.02146)MLSDFKQQK	FIXA	FIXC	207	246	33.6
	6949.570	14.55	2.2	89	55	FGGTVTVVTMGPPMAEAALRKC(57.02146)LSFGADDAILVS(156.0786)DR - GETPGSKNVQGAILYADAIEKIIPDFR	FIXA	FIXC	72	42	33.7
	5969.066	12.13	17.4	55	65	GETPGSK(156.0786)NVQGAILYADAIEKIIPDFR -	FIXC	FIXA	41	49	34.0

						LKDK(156.0786)FGGTVTVVTMGPPMAEAALRK					
	4839.566	11.38	13.6	101	11	EAGVLTICETTVTDLLIEGGKVVGVRTDRQGGEVR - VFAPKPRSK	FIXC	FIXA	139	238	34.5
	5969.066	11.93	17.4	53	65	GETPGSKNVQGAILYADAIEK(156.0786)IIPDFR - LKDK(156.0786)FGGTVTVVTMGPPMAEAALRK	FIXC	FIXA	42	49	34.8
	4094.064	7.10	2.4	34	36	AFAGSDTLATSYALSAVIR - ESITLGVGC(57.02146)MLSDFKQQK	FIXA	FIXC	92	246	35.4
	4094.064	7.14	12.7	58	12	AADKPFSAANLAEYKKK(156.0786)LDDSFVMK - QVPDSAQIR	FIXC	FIXA	350	13	37.0
	4185.174	7.15	2.7	48	22	FDVIVVGAGMAGNAAAYTLAKGGLK - RAELIESHDS(156.0786)DPK	FIXC	FIXA	25	249	38.2
	4094.064	7.25	12.7	59	12	AADKPFSAANLAEYKKKLDDS(156.0786)FVMK - QVPDSAQIR	FIXC	FIXA	351	13	38.5
	5727.949	7.11	6.3	48	22	HELKVWDRVAAGIDTVEMIGLKGSPTVVS(156.0786)K - ES(156.0786)ITLGVGC(57.02146)MLSDFKQQK	FIXA	FIXC	227	246	38.6
	4112.079	8.04	9.8	49	30	DKFGGTVTVVTMGPPMAEAALR - AADKPFSAANLAEYKK	FIXA	FIXC	51	349	39.5
	3340.753	7.03	1.1	57	12	TDRQGGEVRADAVILADGVNSR - KEIQVER	FIXC	FIXA	165	158	40.1
	5967.053	7.11	4.3	57	13	DKFGGTVTVVTMGPPMAEAALRKCLS(156.0786)FGADDAILVSDR - VHFDQWFNKK(156.0786)AR	FIXA	FIXC	72	115	40.4
	5727.934	9.94	5.3	74	24	DKFGGTVTVVTMGPPMAEAALRKC(57.02146)LSFGADDAILVSDR - KLDDS(156.0786)FVMKDLK	FIXA	FIXC	51	351	40.7
	5471.799	7.31	7.5	62	10	DKFGGTVTVVTMGPPMAEAALRKCLS(156.0786)FGADDAILVSDR - KLDDS(156.0786)FVMK	FIXA	FIXC	85	351	40.9
	4185.174	7.11	16.2	54	16	GETPGS(156.0786)KNVQGAILYADAIEKIIPDFR - IVDVDTAKK	FIXC	FIXA	42	157	42.4
	4185.174	7.22	16.2	55	16	GETPGSK(156.0786)NVQGAILYADAIEKIIPDFR - IVDVDTAKK	FIXC	FIXA	41	157	42.6
	4094.064	7.45	11.1	41	32	AFAGSDTLATSYALS(156.0786)AVIR - NKQFFTTYPDLLS(156.0786)R	FIXA	FIXC	98	375	42.6
	6080.245	7.91	17.0	51	27	VHFDQWFNKK(156.0786)AREAGVLTICETTVTDLLIEGGK - MHSVVCIKQVPDS(156.0786)AQIR	FIXC	FIXA	115	8	46.2
	4094.064	7.13	11.1	36	34	AFAGSDTLATS(156.0786)YALS(156.0786)AVIR - NKQFFTTYPDLLSR	FIXA	FIXC	92	375	46.3
	5471.799	7.31	0.8	53	19	QQKISPYELLDRMKTHPS(156.0786)IAPLIAGS(156.0786)EMK - CLS(156.0786)FGADDAILVSDR	FIXC	FIXA	260	85	47.8
	4190.194	9.74	10.9	53	43	VAAGIDTVEMIGLKGSPTVVSK - AADK(156.0786)PFSAANLAEYKK	FIXA	FIXC	232	349	50.1
	4839.566	11.27	1.9	87	24	HELKVWDRVAAGIDTVEMIGLKGSPTVVS(156.0786)K - LDDSFVMKDLK	FIXA	FIXC	227	359	49.7
	4190.194	9.96	10.9	57	41	VAAGIDTVEMIGLKGSPTVVS(156.0786)K - AADKPFSAANLAEYKK	FIXA	FIXC	227	349	50.5
	4839.566	10.86	10.2	77	30	KIMEDMPVDLIFTGKQTIDGDTAQVGPGIAK - VLQIERGETPGSK	FIXA	FIXC	107	41	51.8
	6080.245	7.91	17.0	51	27	VHFDQWFNKK(156.0786)AREAGVLTICETTVTDLLIEGGK - MHSVVCIKQVPDS(156.0786)AQIR	FIXC	FIXA	115	3	51.9
	4190.194	10.06	10.9	58	41	VAAGIDTVEMIGLKGS(156.0786)PTVVSK - AADKPFSAANLAEYKK	FIXA	FIXC	225	349	52.0
	4112.079	7.53	9.8	49	25	DKFGGTVTVVTMGPPMAEAALR - AADKPFSAANLAEYKK	FIXA	FIXC	51	338	52.9
	4112.079	7.34	9.8	49	23	DKFGGTVTVVTMGPPMAEAALR - AADKPFSAANLAEYKK	FIXA	FIXC	51	341	53.2
	6080.245	8.12	17.0	51	29	VHFDQWFNKK(156.0786)AREAGVLTICETTVTDLLIEGGK - MHSVVCIKQVPDS(156.0786)AQIR	FIXC	FIXA	115	1	53.7
	4029.076	7.03	12.5	33	36	AADK(156.0786)PFSAANLAEYK(156.0786)KK - QTIDGDTAQVGPGIAKR	FIXC	FIXA	350	137	53.8
	6080.252	7.29	5.4	34	38	GETPGSKNVQGAILYADAIEK(156.0786)IIPDFR - VWDRVAAGIDTVEMIGLKGSPTVVS(156.0786)K	FIXC	FIXA	42	227	54.3
	5412.792	8.42	18.5	67	16	VWDRVAAGIDTVEMIGLKGSPTVVSKVFAPK(156.0786)PR - AADKPFSAANLAEYK	FIXA	FIXC	233	341	55.7
	5412.792	8.83	18.5	67	20	VWDRVAAGIDTVEMIGLKGSPTVVSKVFAPK(156.0786)PR - AADKPFSAANLAEYK	FIXA	FIXC	233	338	56.8
	6080.252	7.19	5.4	36	35	GETPGSKNVQGAILYADAIEK(156.0786)IIPDFR - VWDRVAAGIDTVEMIGLKGSPTVVS(156.0786)K	FIXC	FIXA	41	225	57.0
	4034.089	9.55	17.8	57	37	VAAGIDTVEMIGLKGSPTVVSK - AADKPFSAANLAEYKK	FIXA	FIXC	232	338	57.2
	6949.570	14.24	13.2	127	14	MKTHPSIAPLIAGSEMKEYAAHLIPEGGYNAIPQVYGDGWMIAGDAGQFVNGIHR - GS(156.0786)PTVVSK	FIXC	FIXA	260	232	57.2
	4094.064	7.24	16.3	55	16	MAERFDVIVVGAGMAGNAAAYTLAKGGLK - MHS(156.0786)VVCIK	FIXC	FIXA	25	1	58.0
	4700.564	7.94	6.4	71	7	FDVIVVGAGMAGNAAAYTLAKGGLKVLQIERGETPGSK - GSPTVVSK	FIXC	FIXA	29	227	59.4
	4839.612	14.20	17.4	103	37	FDVIVVGAGMAGNAAAYTLAKGGLKVLQIER - GSPTVVSKVFAPKPR	FIXC	FIXA	29	227	59.4
	4190.183	8.35	13.4	53	29	VAAGIDTVEMIGLKGSPTVVS(156.0786)K - AADKPFSAANLAEYKK	FIXA	FIXC	225	341	60.0
	4190.183	8.96	13.4	55	33	VAAGIDTVEMIGLKGSPTVVS(156.0786)K - AADKPFSAANLAEYKK	FIXA	FIXC	227	338	60.4
	4700.564	7.83	6.4	71	6	FDVIVVGAGMAGNAAAYTLAKGGLKVLQIERGETPGSK - GSPTVVSK	FIXC	FIXA	25	232	60.9
	4190.183	8.75	13.4	53	33	VAAGIDTVEMIGLKGSPTVVS(156.0786)K - AADKPFSAANLAEYKK	FIXA	FIXC	225	338	61.7
	4029.075	7.01	12.8	35	34	AADK(156.0786)PFSAANLAEYKK(156.0786)K - QTIDGDTAQVGPGIAKR	FIXC	FIXA	341	137	62.8
	4029.076	7.02	12.5	33	36	AADKPFSAANLAEYK(156.0786)K(156.0786)K - QTIDGDTAQVGPGIAKR	FIXC	FIXA	338	137	63.6
	6080.255	9.23	0.4	55	36	HELKVWDRVAAGIDTVEMIGLK(156.0786)GSPTVVS(156.0786)K - FDVIVVGAGMAGNAAAYTLAKGGLK	FIXA	FIXC	227	25	64.9
	5727.939	12.96	8.2	84	44	HELK(156.0786)VWDRVAAGIDTVEMIGLKGSPTVVS(156.0786)K - ESITLGVGC(57.02146)MLSDFKQQK	FIXA	FIXC	225	233	65.3
	6080.252	7.19	0.9	42	29	HELK(156.0786)VWDRVAAGIDTVEMIGLKGSPTVVS(156.0786)K - FDVIVVGAGMAGNAAAYTLAKGGLK	FIXA	FIXC	225	25	67.5
**B with C**											
	5471.799	7.72	3.5	45	31	VLGAEVGASRPIVQAGWAELDRQVGQS(156.0786)GK - ES(156.0786)ITLGVGC(57.02146)MLSDFKQQK	FIXB	FIXC	267	246	13.7
	5727.944	8.53	17.9	55	29	ES(156.0786)ITLGVGCMLSDFKQQKIS(156.0786)PYELLDR - TVRPKLYIAAGIS(156.0786)GAIQHR	FIXC	FIXB	246	292	30.2
	5727.944	8.93	17.9	59	29	ESITLGVGCMLSDFK(156.0786)QQKIS(156.0786)PYELLDR - TVRPKLYIAAGIS(156.0786)GAIQHR	FIXC	FIXB	249	292	31.2
	5727.943	8.23	16.7	44	37	QQKISPYELLDRMKTHPSIAPLIAGSEMK - NLASTRPTFGGSLLC(57.02146)TILTQR	FIXC	FIXB	249	166	34.1
	5500.014	14.17	16.3	78	62	VLQIERGETPGSKNVQGAILYADAIEK - NLAS(156.0786)TRPTFGGSLLC(57.02146)TILTQR	FIXC	FIXB	42	166	37.4
	6949.570	14.97	2.8	121	27	DLAGSVATTLGTGLVADC(57.02146)TELVIDTETRNLASTRPTFGGSLLC(57.02146)TILTQR - QQKIS(156.0786)PYELLDR	FIXB	FIXC	131	249	37.7
	4185.177	7.33	8.9	50	22	VLGAEVGASRPIVQAGWAELDR - LDDS(156.0786)FVMKDLK(156.0786)K	FIXB	FIXC	267	359	40.1
	5727.949	7.03	5.0	56	13	K(156.0786)LADALGVELYGVVICGPGERGKEIC(57.02146)GEAFQHGADK - QQKIS(156.0786)PYELLDR	FIXB	FIXC	71	249	45.1
	7024.433	7.81	10.2	40	37	LADALGVELYGVVICGPGERGKEICGEAFQHGADK - ESITLGVGC(57.02146)MLSDFKQQKIS(156.0786)PYELLDR	FIXB	FIXC	71	246	43.5
	4034.089	8.95	16.4	58	30	NLASTRPTFGGSLLCTILTQR - QQKIS(156.0786)PYELLDR	FIXB	FIXC	158	249	44.0
	4185.177	7.02	8.9	50	19	VLGAEVGASRPIVQAGWAELDR - KLDDS(156.0786)FVMK(156.0786)DLK	FIXB	FIXC	267	351	44.8
	4839.566	13.12	3.3	57	72	FDVIVVGAGMAGNAAAYTLAKGGLK - NLASTRPTFGGSLLC(57.02146)TILTQR	FIXC	FIXB	25	166	47.3
	4185.177	8.35	0.3	52	30	VLGAEVGASRPIVQAGWAELDR - AADKPFS(156.0786)AANLAEYK	FIXB	FIXC	267	338	49.3
	4839.566	11.48	4.6	82	31	VLGAEVGASRPIVQAGWAELDRQVGQSGK - AADKPFSAANLAEYKK	FIXB	FIXC	285	338	50.8
	4190.194	10.65	9.3	66	39	KLADALGVELYGVVICGPGERGK - THPS(156.0786)IAPLIAGSEMK	FIXB	FIXC	49	272	60.9
	5471.799	7.43	10.0	49	24	IS(156.0786)PYELLDRMKTHPSIAPLIAGSEMK - SGEIIEVPFS(156.0786)MIETDIITK	FIXC	FIXB	260	198	61.8
	4839.566	12.30	3.3	57	64	FDVIVVGAGMAGNAAAYTLAKGGLK - NLASTRPTFGGSLLC(57.02146)TILTQR	FIXC	FIXB	25	158	63.3
	4112.079	7.05	11.3	49	20	MPEIFHKNKQFFTTYPDLLS(156.0786)R - VMAMPEPDASR	FIXC	FIXB	375	196	72.2
	5412.792	8.12	14.8	64	16	KLADALGVELYGVVICGPGERGKEIC(57.02146)GEAFQHGADK - QFFTTYPDLLSR	FIXB	FIXC	71	386	72.3
	4185.186	7.32	11.5	37	35	SGEIIEVPFSMIETDIITK - GGLKVLQIERGETPGS(156.0786)K	FIXB	FIXC	198	29	79.5
	4112.079	7.24	19.7	36	35	SGEIIEVPFSMIETDIITK - AADKPFSAANLAEYKKK	FIXB	FIXC	198	350	82.1
**A with X**											
	7078.324	10.42	1.7	98	5	QCTVCCPAGCYTRETNGKVTLVTDGCLECGTC(57.02146)RIICQDSGNLEWEWPR - GSPTVVSKVFAPKPR	FIXX	FIXA	54	232	18.7
	7078.324	10.32	1.7	98	4	QCTVCCPAGCYTRETNGKVTLVTDGCLECGTC(57.02146)RIICQDSGNLEWEWPR - GSPTVVSKVFAPKPR	FIXX	FIXA	54	233	19.5
	5727.934	9.94	0.2	58	40	VWDRVAAGIDTVEMIGLKGSPTVVS(156.0786)K - VDS(156.0786)GRPHIRIKDPDHCTECLEK	FIXA	FIXX	225	25	20.9
	5727.934	10.34	0.2	62	40	VWDRVAAGIDTVEMIGLKGSPTVVS(156.0786)K - VDS(156.0786)GRPHIRIKDPDHCTECLEK	FIXA	FIXX	227	25	21.9
	7063.375	7.41	11.7	42	31	ETNGKVTLVTDGC(57.02146)LECGTCRIIC(57.02146)QDSGNLEWEWPR - VWDRVAAGIDTVEMIGLKGS(156.0786)PTVVSK	FIXX	FIXA	54	225	22.6
	7078.324	10.72	1.7	98	8	QCTVCCPAGCYTRETNGKVTLVTDGCLECGTC(57.02146)RIICQDSGNLEWEWPR - GSPTVVSKVFAPKPR	FIXX	FIXA	54	227	24.7
	7429.417	18.85	12.3	100	87	KCLSFGADDAILVSDRAFAGSDTLATSYALSAVIRK - IKDPDHCTECLEKQCTVC(57.02146)CPAGC(57.02146)YTRETNGK	FIXA	FIXX	72	36	28.4
	7078.324	10.72	1.7	98	8	QCTVCCPAGCYTRETNGKVTLVTDGCLECGTC(57.02146)RIICQDSGNLEWEWPR - GSPTVVSKVFAPKPR	FIXX	FIXA	54	238	29.2
	7024.429	11.92	13.2	58	60	K(156.0786)IMEDMPVDLIFTGK(156.0786)QTIDGDTAQVGPGIAKR - IK(156.0786)DPDHCTECLEKQCTVCCPAGC(57.02146)YTR	FIXA	FIXX	137	36	33.4
	4700.564	8.13	3.9	63	17	VWDRVAAGIDTVEMIGLKGSPTVVSKVFAPKPR - VDSGRPHIR	FIXA	FIXX	233	17	34.3
	7429.417	18.75	6.1	142	44	QCTVCCPAGCYTRETNGKVTLVTDGC(57.02146)LECGTCRIIC(57.02146)QDSGNLEWEWPR - KCLSFGADDAILVS(156.0786)DR	FIXX	FIXA	54	72	34.7
	4700.564	8.43	3.9	66	17	VWDRVAAGIDTVEMIGLKGSPTVVSKVFAPKPR - VDSGRPHIR	FIXA	FIXX	238	17	37.8
	5727.934	9.94	0.2	58	40	VWDRVAAGIDTVEMIGLKGSPTVVS(156.0786)K - VDSGRPHIRIK(156.0786)DPDHCTECLEK	FIXA	FIXX	225	17	38.3
	5727.944	8.54	8.6	63	21	FGGTVTVVTMGPPMAEAALRKC(57.02146)LS(156.0786)FGADDAILVSDR - MSKIEEK(156.0786)LFQDR	FIXA	FIXX	72	3	41.5
	5727.943	7.41	5.4	39	34	YRVDSGRPHIRIKDPDHC(57.02146)TECLEK - VFAPKPRSKRAELIESHDS(156.0786)DPK	FIXX	FIXA	25	249	53.9
**B with X**											
	7429.417	19.05	6.4	100	89	KLADALGVELYGVVICGPGERGKEICGEAFQHGADK - IKDPDHCTECLEKQC(57.02146)TVCCPAGC(57.02146)YTRETNGK	FIXB	FIXX	49	36	34.8
	5471.799	7.31	1.4	35	37	IICQDSGNLEWEWPRGGFGILFKFG - NLAS(156.0786)TRPTFGGSLLC(57.02146)TILTQR	FIXX	FIXB	92	166	36.8
	7429.417	18.94	6.4	100	88	KLADALGVELYGVVICGPGERGKEICGEAFQHGADK - IKDPDHC(57.02146)TECLEKQCTVCCPAGC(57.02146)YTRETNGK	FIXB	FIXX	49	25	44.8
	7429.417	18.96	14.0	106	82	ETNGKVTLVTDGCLECGTCRIIC(57.02146)QDSGNLEWEWPR - VMAMPEPDASRSGEIIEVPFSMIETDIITK	FIXX	FIXB	54	207	53.5
	7429.417	19.35	6.4	105	87	KLADALGVELYGVVICGPGERGKEICGEAFQHGADK - IKDPDHCTECLEKQC(57.02146)TVCCPAGC(57.02146)YTRETNGK	FIXB	FIXX	71	25	56.0

	Experimental M+H	Primary Score	ppm	Peaks Matched Peptide 1	Peaks Matched Peptide 2	Peptide Sequence (additional mass modifications indicated)	Cross-linked residue Peptide 1	Cross-linked residue Peptide 2	Cα to Cα Distance
**FixA**									
	5727.934	10.14	16.6	77	23	FGGTVTVVTMGPPMAEAALRKCLSFGADDAILVS(156.0786)DR - CLSFGADDAILVS(156.0786)DR	72	75	5.1
	4185.176	7.35	15.6	33	39	MHSVVC(57.02146)IKQVPDS(156.0786)AQIR - KC(57.02146)LSFGADDAILVS(156.0786)DR	8	72	11.9
	4185.176	7.04	15.6	33	36	MHSVVC(57.02146)IKQVPDS(156.0786)AQIR - KC(57.02146)LS(156.0786)FGADDAILVSDR	8	85	13.0
	4185.174	7.15	5.3	47	23	NLAEAALAKLFTQHPNLEQEIAK - AELIESHDS(156.0786)DPK	264	249	14.3
	5471.799	7.51	4.0	65	9	DK(156.0786)FGGTVTVVTMGPPMAEAALRKCLS(156.0786)FGADDAILVSDR - HELKVWDR	72	207	14.8
	5969.066	12.64	8.1	95	30	DKFGGTVTVVTMGPPMAEAALRKCLS(156.0786)FGADDAILVSDR - KIMEDMPVDLIFTGK	85	107	14.9
	2866.561	3.21	7.7	31		VWDRVAAGIDTVEMIGLKGSPTVVSK	225	232	15.2
	5500.014	14.58	17.7	94	50	HELKVWDRVAAGIDTVEMIGLKGSPTVVS(156.0786)K - MHSVVC(57.02146)IKQVPDSAQIR	207	3	15.7
	4185.177	7.76	15.7	39	37	MHSVVC(57.02146)IKQVPDS(156.0786)AQIR - KC(57.02146)LSFGADDAILVS(156.0786)DR	8	75	16.0
	5471.799	7.31	4.0	63	9	DKFGGTVTVVTMGPPMAEAALRK(156.0786)CLSFGADDAILVS(156.0786)DR - HELKVWDR	75	207	16.1
	4185.176	7.45	15.6	34	39	MHSVVC(57.02146)IKQVPDS(156.0786)AQIR - KC(57.02146)LSFGADDAILVS(156.0786)DR	3	72	16.8
	4185.174	7.03	7.9	57	12	MHSVVC(57.02146)IKQVPDSAQIRVHPVTNTIMR - GSPTVVS(156.0786)K	8	227	19.0
	5500.014	14.39	17.7	104	38	HELKVWDRVAAGIDTVEMIGLKGSPTVVS(156.0786)K - MHSVVC(57.02146)IKQVPDSAQIR	227	8	19.0
	5500.014	14.78	17.7	94	52	HELKVWDRVAAGIDTVEMIGLKGSPTVVS(156.0786)K - MHSVVC(57.02146)IKQVPDSAQIR	207	1	19.5
	4185.176	7.65	15.6	36	39	MHSVVC(57.02146)IKQVPDS(156.0786)AQIR - KC(57.02146)LSFGADDAILVS(156.0786)DR	1	72	20.7
	5727.934	10.16	5.6	74	26	FGGTVTVVTMGPPMAEAALRKCLS(156.0786)FGADDAILVSDR - KIMEDMPVDLIFTGK	72	107	23.1
	5727.934	9.95	5.6	72	26	FGGTVTVVTMGPPMAEAALRKCLSFGADDAILVS(156.0786)DR - KIMEDMPVDLIFTGK	75	107	24.1
	6080.245	8.03	9.6	49	30	VAAGIDTVEMIGLKGSPTVVSKVFAPK(156.0786)PR - VFAPK(156.0786)PRSKRAELIESHDS(156.0786)DPK	233	242	25.8
	5471.799	7.62	4.0	66	9	DKFGGTVTVVTMGPPMAEAALRK(156.0786)CLS(156.0786)FGADDAILVSDR - HELKVWDR	51	207	26.3
	4112.079	7.03	9.0	38	31	DKFGGTVTVVTMGPPMAEAALR - QTIDGDTAQVGPGIAKR	51	137	26.6
	5969.066	12.05	8.1	89	30	DKFGGTVTVVTMGPPMAEAALRK(156.0786)CLSFGADDAILVSDR - KIMEDMPVDLIFTGK	51	107	26.6
	5727.939	12.64	1.6	84	41	C(57.02146)LSFGADDAILVSDRAFAGSDTLATSYALS(156.0786)AVIR - IVDVDTAKK(156.0786)EIQVER	75	157	27.1
	6080.245	7.93	9.6	48	30	VAAGIDTVEMIGLKGSPTVVSKVFAPK(156.0786)PR - VFAPK(156.0786)PRSKRAELIESHDS(156.0786)DPK	232	242	28.7
	5500.014	14.69	17.7	95	50	HELKVWDRVAAGIDTVEMIGLKGSPTVVS(156.0786)K - MHSVVC(57.02146)IKQVPDSAQIR	225	3	30.9
	6080.255	9.21	12.0	48	43	HELKVWDRVAAGIDTVEMIGLKGSPTVVSK - PMAEAALRKCLS(156.0786)FGADDAILVSDR	232	72	34.1
	6949.570	14.33	17.6	76	66	VWDRVAAGIDTVEMIGLKGSPTVVSKVFAPKPR - KIMEDMPVDLIFTGKQTIDGDTAQVGPGIAK	233	107	36.4
	5500.014	14.07	10.9	92	47	VAAGIDTVEMIGLKGSPTVVSKVFAPK(156.0786)PRSK - K(156.0786)CLSFGADDAILVS(156.0786)DR	225	75	36.5
	6080.255	9.14	10.0	59	31	C(57.02146)LSFGADDAILVSDRAFAGS(156.0786)DTLATSYALSAVIR - GSPTVVSK(156.0786)VFAPKPRSK(156.0786)R	102	238	37.3
	6080.245	7.82	9.6	46	31	VAAGIDTVEMIGLKGSPTVVSKVFAPK(156.0786)PR - VFAPKPRSKRAELIES(156.0786)HDS(156.0786)DPK	227	242	39.0
	6080.245	8.03	9.6	49	30	VAAGIDTVEMIGLKGSPTVVSKVFAPK(156.0786)PR - VFAPKPRS(156.0786)KRAELIESHDS(156.0786)DPK	233	249	46.0
	4190.183	8.44	5.6	49	34	LDYQLLTYVSRIVDVDTAKK - SKRAELIESHDSDPK	157	241	52.0
	5500.014	14.88	2.1	73	74	NLAEAALAKLFTQHPNLEQEIAK(156.0786)RAV - VAAGIDTVEMIGLKGSPTVVS(156.0786)K	264	227	53.4
	5500.014	14.88	2.1	73	74	NLAEAALAKLFTQHPNLEQEIAK(156.0786)RAV - VAAGIDTVEMIGLKGSPTVVS(156.0786)K	264	225	58.2
	6080.245	7.72	9.6	43	33	VAAGIDTVEMIGLKGSPTVVSK(156.0786)VFAPK(156.0786)PR - VFAPKPRSKRAELIESHDS(156.0786)DPK	225	249	60.1
**FixB**									
	6080.255	9.13	13.3	58	32	NLAS(156.0786)TRPTFGGSLLCTILTQRHRPQMATVRPR - TVRPKLYIAAGIS(156.0786)GAIQHR	166	292	21.6
	4034.089	9.04	14.3	62	27	NLASTRPTFGGSLLC(57.02146)TILTQR - GKEICGEAFQHGADK	166	71	38.8
**FixC**									
	7078.324	9.81	14.0	47	50	ISAGMMGTGFLYTNKESITLGVGCMLSDFK(156.0786)QQK - ISAGMMGTGFLYTNKESITLGVGC(57.02146)MLSDFK	231	233	5.4
	5471.803	7.03	11.1	49	20	ISPYELLDRMKTHPS(156.0786)IAPLIAGS(156.0786)EMK - MK(156.0786)THPS(156.0786)IAPLIAGSEMK	260	272	9.4
	4186.197	8.42	16.7	51	32	THPSIAPLIAGSEMKEYAAHLIP - ESITLGVGCMLSDFK	275	233	10.5
	6080.252	7.29	0.9	57	15	FDVIVVGAGMAGNAAAYTLAKGGLKVLQIERGETPGS(156.0786)K - YTIIRVHFDQWFNKK	25	115	13.6
	6080.255	9.32	1.4	64	28	FDVIVVGAGMAGNAAAYTLAKGGLK(156.0786)VLQIERGETPGSK - YTIIRVHFDQWFNKK	41	115	14.1
	6080.252	7.29	0.9	57	15	FDVIVVGAGMAGNAAAYTLAKGGLKVLQIERGETPGS(156.0786)K - YTIIRVHFDQWFNKK	29	115	17.1
	4034.089	9.14	0.6	59	31	TDRQGGEVRADAVILADGVNSR - K(156.0786)LDDSFVMKDLK	165	359	18.5
	4029.075	7.12	11.0	60	10	VVGVRTDRQGGEVRADAVILADGVNSR - KLDDSFVMK	165	351	21.1
	4185.176	7.63	5.1	58	17	VVGVRTDRQGGEVRADAVILADGVNSR - KLDDS(156.0786)FVMK	165	351	21.1
	4112.079	7.32	6.1	40	32	GETPGSKNVQGAILYADAIEK - ES(156.0786)ITLGVGC(57.02146)MLSDFK	42	243	21.4
	6080.255	9.70	5.9	58	38	FDVIVVGAGMAGNAAAYTLAKGGLKVLQIER - VVGVRTDRQGGEVRADAVILADGVNSR	29	165	23.2
	4112.079	7.13	14.9	38	32	GETPGSKNVQGAILYADAIEK - MKTHPSIAPLIAGSEMK	42	272	23.9
	4839.612	14.39	4.4	105	37	FDVIVVGAGMAGNAAAYTLAKGGLK(156.0786)VLQIER - VLQIERGETPGSK	25	41	24.7
	4839.566	12.08	14.0	85	34	FDVIVVGAGMAGNAAAYTLAK(156.0786)GGLKVLQIER - VLQIERGETPGSK	29	41	25.4
	3165.743	2.60	3.9	25		KAREAGVLTICETTVTDLLIEGGKVVGVR	116	139	26.9
	6080.255	9.31	11.2	40	52	MAERFDVIVVGAGMAGNAAAYTLAKGGLK - GETPGSKNVQGAILYADAIEK(156.0786)IIPDFR	25	42	27.6
	4112.079	7.52	14.9	38	36	GETPGSKNVQGAILYADAIEK - MKTHPSIAPLIAGSEMK	42	260	29.0
	4094.064	7.14	4.2	37	33	ESITLGVGC(57.02146)MLSDFKQQK - LDDSFVMKDLK(156.0786)KYR	246	359	29.0
	4186.197	8.43	0.8	67	16	GETPGSKNVQGAILYADAIEKIIPDFR - MPEIFHKNK	41	373	29.8
	4112.079	7.42	14.9	37	36	GETPGSKNVQGAILYADAIEK - MKTHPSIAPLIAGSEMK	41	260	30.5
	4186.197	8.33	0.8	66	16	GETPGSKNVQGAILYADAIEKIIPDFR - MPEIFHKNK	42	373	30.5
	6949.570	14.16	4.6	69	71	AADKPFSAANLAEYK(156.0786)KKLDDSFVMKDLKK - ESITLGVGCMLSDFK(156.0786)QQKIS(156.0786)PYELLDR	362	249	30.8
	4094.064	7.12	4.2	36	34	ESITLGVGC(57.02146)MLSDFKQQK - LDDSFVMKDLKK(156.0786)YR	233	362	31.1
	4186.197	8.44	3.6	76	7	ESITLGVGCMLSDFKQQKIS(156.0786)PYELLDR - DLKKYR	249	362	33.4
	4186.197	8.53	9.2	57	27	VHFDQWFNKKAREAGVLTIC(57.02146)ETTVTDLLIEGGK - ES(156.0786)ITLGVGC(57.02146)MLSDFK	115	243	33.7
	4034.089	9.86	4.0	50	47	QGGEVRADAVILADGVNSR - MKTHPS(156.0786)IAPLIAGSEMK	165	260	36.2
	5727.943	7.61	9.5	56	19	ISAGMMGTGFLYTNKESITLGVGCMLS(156.0786)DFK(156.0786)QQK - KLDDSFVMKDLK(156.0786)K	231	359	37.1
	4190.194	10.06	10.4	68	31	FDVIVVGAGMAGNAAAYTLAKGGLK - ESITLGVGC(57.02146)MLSDFK	25	233	38.0
	4186.197	9.03	12.3	44	45	ESITLGVGC(57.02146)MLSDFKQQK - AADKPFSAANLAEYKK(156.0786)K	233	349	40.0
	5727.943	7.61	2.3	58	17	FDVIVVGAGMAGNAAAYTLAKGGLKVLQIERGETPGSK - VWVMDDASYVGTHYR	25	83	40.0
	6949.570	14.16	13.8	73	67	ISAGMMGTGFLYTNKES(156.0786)ITLGVGCMLSDFKQQK - AADKPFSAANLAEYKK(156.0786)KLDDS(156.0786)FVMK	218	351	40.1
	4190.194	10.37	10.4	68	34	FDVIVVGAGMAGNAAAYTLAKGGLK - ESITLGVGC(57.02146)MLSDFK	25	243	40.5
	5727.943	7.51	9.5	56	18	ISAGMMGTGFLYTNKESITLGVGCMLS(156.0786)DFK(156.0786)QQK - KKLDDSFVMK(156.0786)DLK	231	351	40.8
	4186.197	8.32	12.3	46	36	ESITLGVGC(57.02146)MLSDFK(156.0786)QQK - AADKPFSAANLAEYKKK	233	350	41.7
	5727.943	7.52	15.5	43	31	IS(156.0786)PYELLDRMKTHPSIAPLIAGS(156.0786)EMK - YTIIRVHFDQWFNKK(156.0786)AR	260	115	43.1
	5727.943	7.41	9.5	56	17	ISAGMMGTGFLYTNKESITLGVGCMLS(156.0786)DFK(156.0786)QQK - KKLDDSFVMK(156.0786)DLK	231	350	43.6
	5727.939	13.36	13.3	110	22	FDVIVVGAGMAGNAAAYTLAKGGLK(156.0786)VLQIERGETPGS(156.0786)K - QFFTTYPDLLSR	25	386	44.5
	5727.943	7.40	2.3	56	17	FDVIVVGAGMAGNAAAYTLAKGGLKVLQIERGETPGSK - VWVMDDASYVGTHYR	29	83	45.6
	4839.566	11.18	15.4	74	36	EAGVLTICETTVTDLLIEGGKVVGVRTDR - ESITLGVGC(57.02146)MLSDFK	139	233	47.5
	5727.939	12.64	3.2	104	21	EGSNLAMTTGMLAAQTLVELRAADKPFS(156.0786)AANLAEYK(156.0786)K - ISPYELLDRMK	338	251	48.5
	5727.939	13.44	13.3	111	22	FDVIVVGAGMAGNAAAYTLAK(156.0786)GGLKVLQIERGETPGS(156.0786)K - QFFTTYPDLLSR	29	386	49.7
	5969.066	12.36	16.4	71	51	QQK(156.0786)ISPYELLDRMKTHPSIAPLIAGSEMK - FDVIVVGAGMAGNAAAYTLAKGGLK	260	25	52.4
	6950.565	8.31	2.4	49	33	ISAGMMGTGFLYTNKESITLGVGCMLSDFKQQK - GGLKVLQIERGETPGSKNVQGAILYADAIEK	218	29	54.3

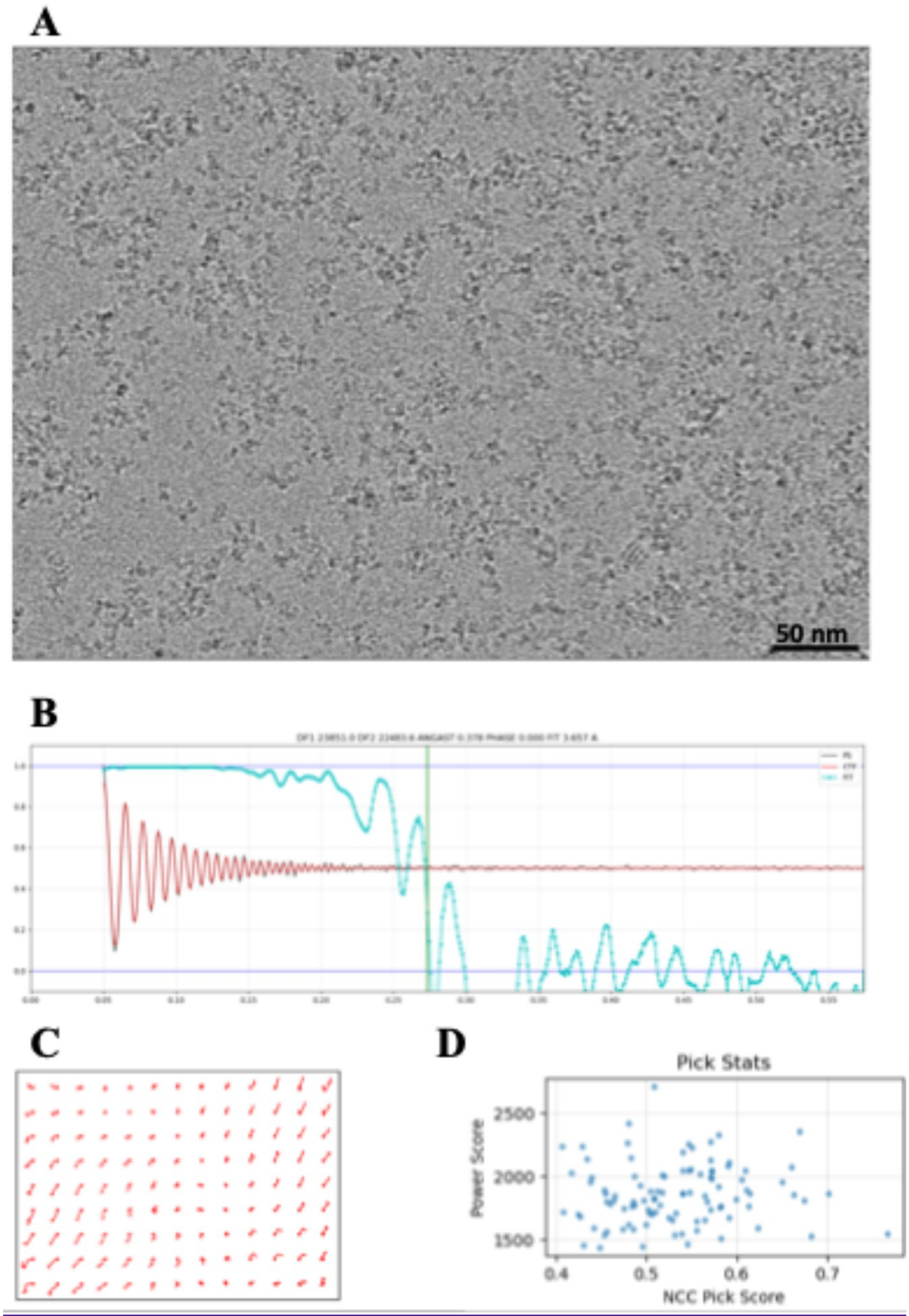

Figure S1. A) An exemplar micrograph. The scale bar (bottom right) is 50 nm. In addition to individual particles, regions of aggregated particles are also present. B) The 1D CTF plot showing the preliminary fit to 3.7 Å with an average defocus of -2.3 microns. C) The local

motion trajectory for this micrograph. D) The pick stats for this image, using only particles contributing to the final structure.

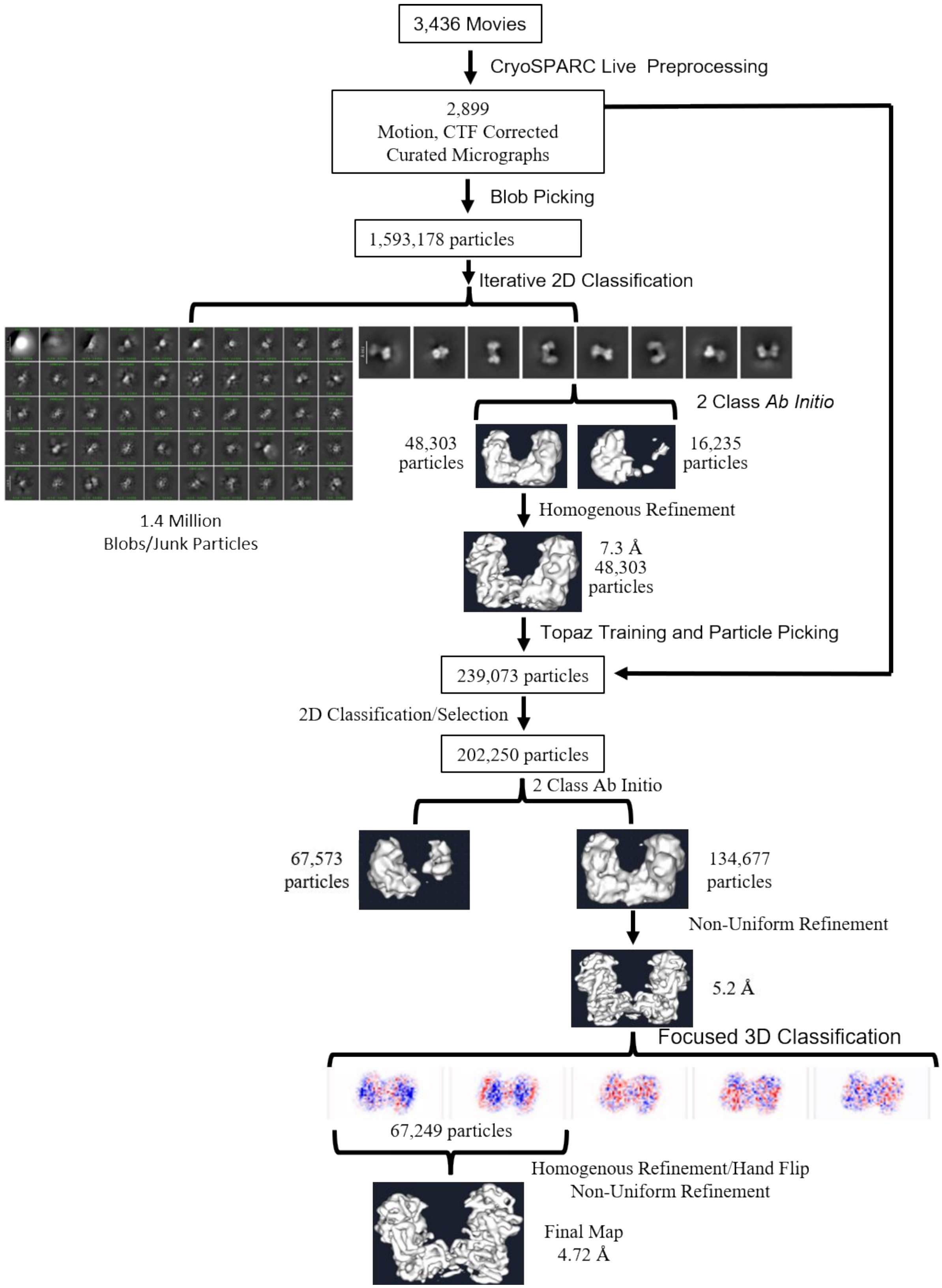

Figure S2: Single particle reconstruction workflow for *Av*FixABCX. See Methods for details.

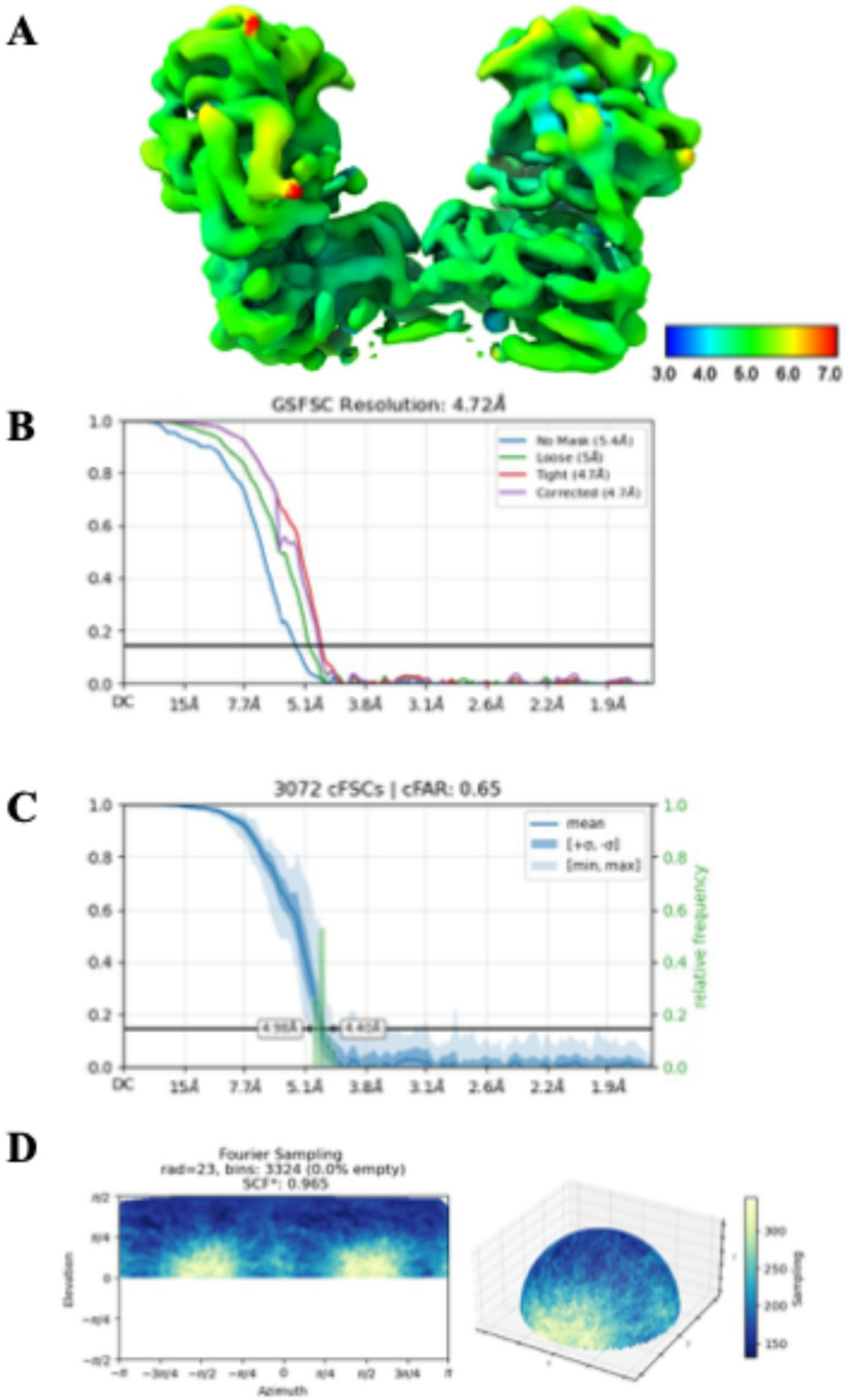
A
3.0 4.0 5.0 6.0 7.0
B
GSFSC Resolution: 4.72Å
No Mask (5.4Å)
Loose (5Å)
Tight (4.7Å)
Corrected (4.7Å)
DC 15Å 7.7Å 5.1Å 3.8Å 3.1Å 2.6Å 2.2Å 1.9Å
C
3072 cFSCs | cFAR: 0.65
mean
[+σ, -σ]
[min, max]
relative frequency
4.98Å
4.40Å
DC 15Å 7.7Å 5.1Å 3.8Å 3.1Å 2.6Å 2.2Å 1.9Å
D
Fourier Sampling
rad=23, bins: 3324 (0.0% empty)
SCF*: 0.965
Elevation
Azimuth
Sampling

Figure S3: Local Resolution and Orientation Diagnostics. A) The local resolution map with a color-coded key, from 3.0 Å (dark blue) to 7.0 Å (red) in the bottom right. Mean resolution is 4.72 Å (green). B) The gold standard FSC curve for the final map, indicating a final resolution of 4.72 Å C) The conical FSC area ratio for FixABCX is 0.65, where 0 indicates severe orientation bias, and 1 indicates no bias. CryoSPARC documentation suggests a cFAR of 0.5 “serves as a reasonable threshold for the presence of a preferred orientation”. In addition, minimum and maximum directional resolutions of 4.98 Å and 4.40 Å are also indicated. D) The Sampling Compensation Factor (SCF) for the FixABCX data set is 0.965. Values above 0.81 generally indicate good, though not necessarily isotropic signal content (Baldwin and Lyumkis 2021).

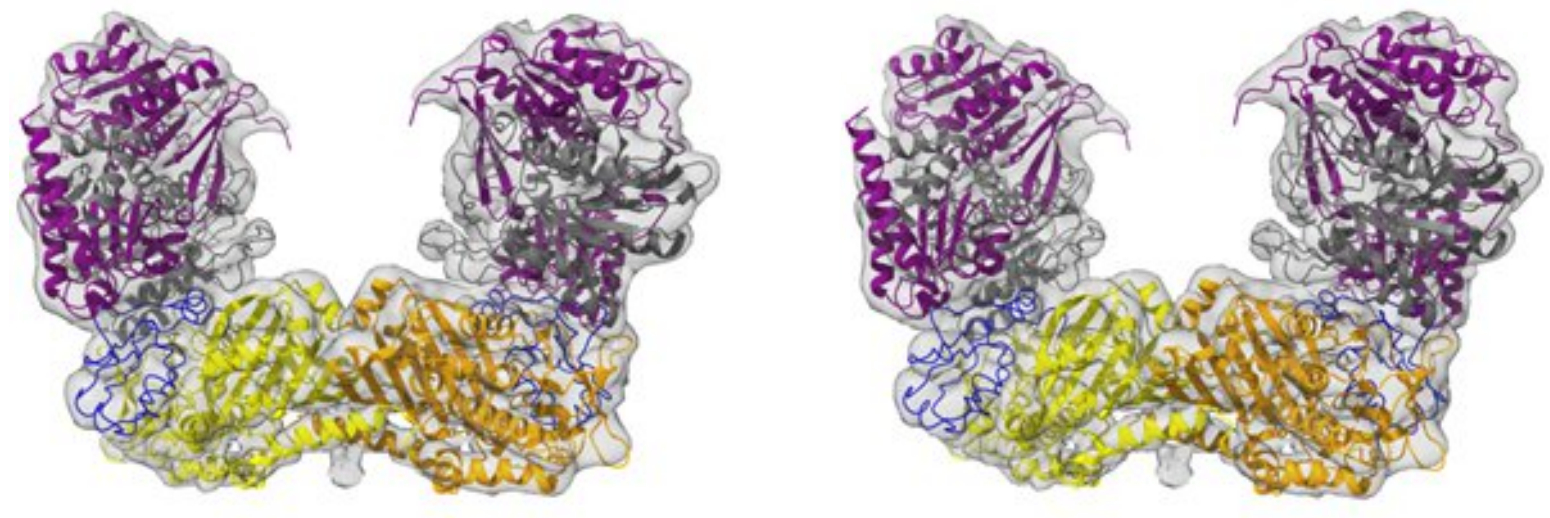

Figure S4: Model Fit to Map (Stereo Figure). The transparent potential-density isosurface contoured at 5 σ is shown in light grey, with FixA in dark grey, FixB in purple, FixC in yellow and orange, and FixX in blue. The masked map-to-model correlation is 0.72.

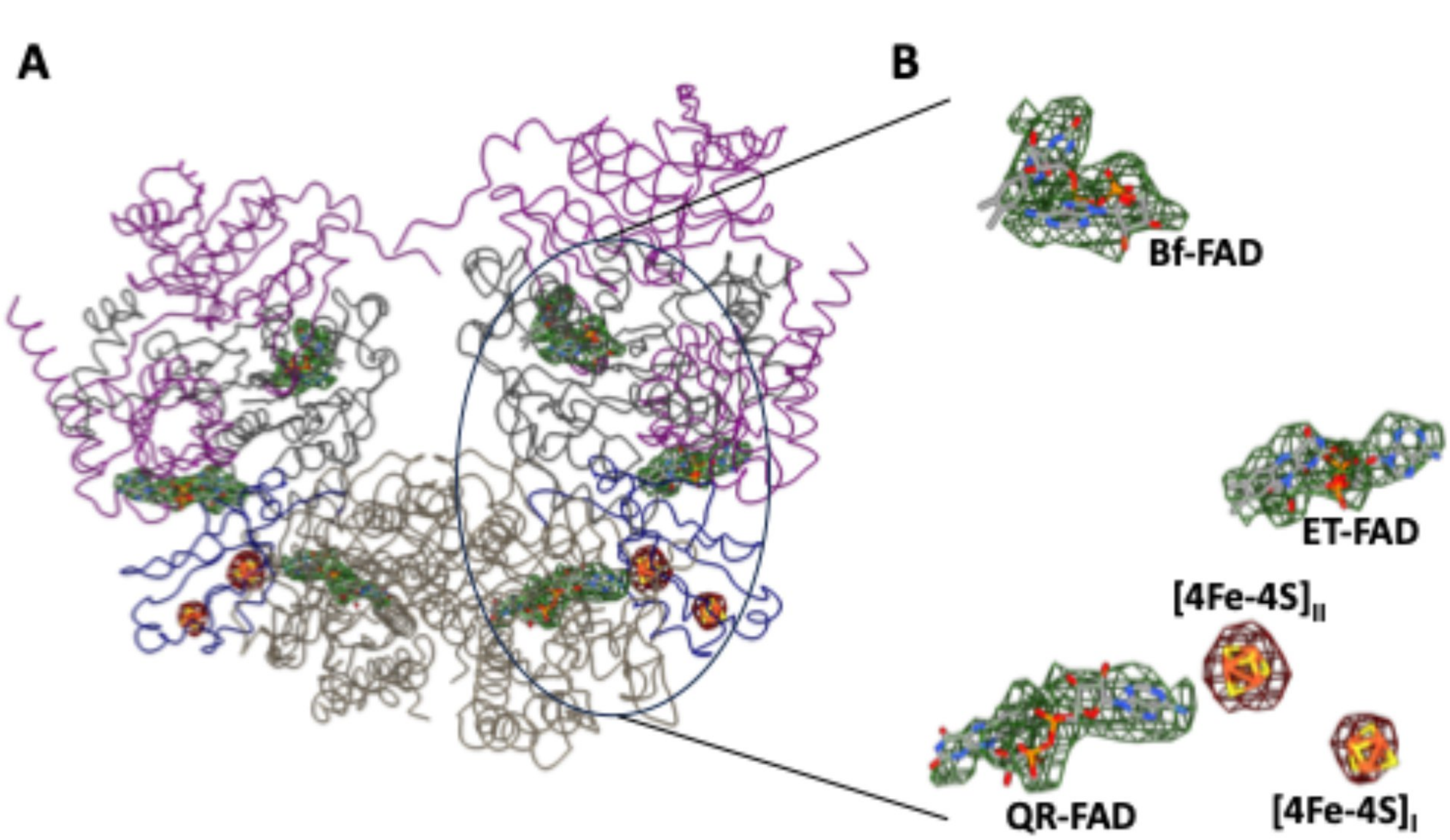

Figure S5. *Av*FixABCX Cofactor Density. A) The FixABCX octamer is shown as a ribbon, colored by subunit type. The 2-fold axis runs vertically in the plane of the page, with the right side protomer rotated toward the viewer, and the left side protomer away. Density for each FAD cofactor is shown as green isonet contoured at 5 sigma, and density for the 4Fe-4S clusters is in rust colored isonet contoured at 10 sigma. B). An enlarged view of the FAD and iron-sulfur clusters in the right protomer; the protein is hidden for clarity.

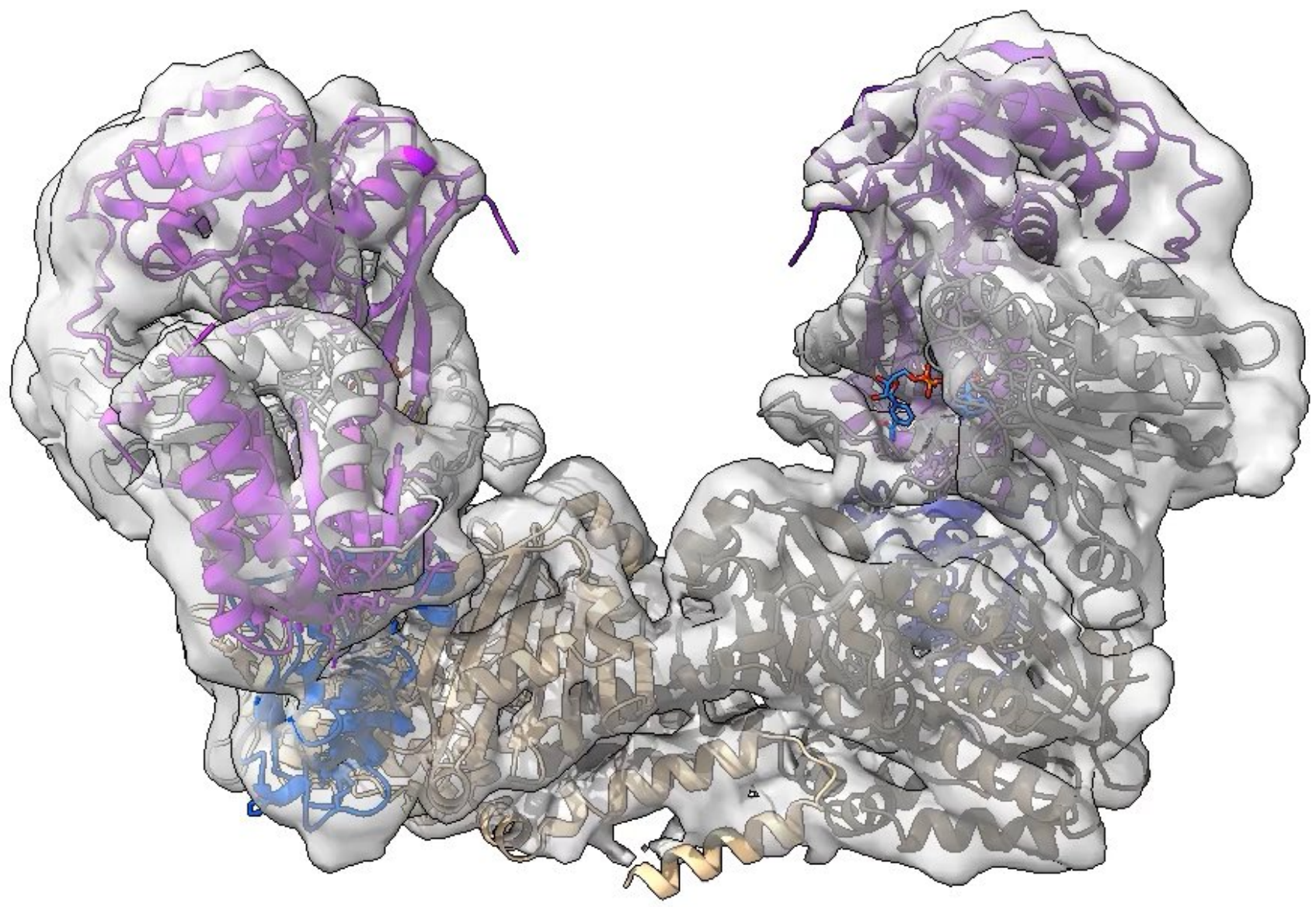

Movie S1: 3D variability analysis in *Av*FixABCX cryo-EM particles.

3D Variability analysis of the compact structure determined by cryo-EM shows significant conformational heterogeneity, with the arms of the U-shaped structure oscillating towards and away from each other. Docking studies suggest Fld interacts with each protomer, linking the arms of the U-shaped structure, potentially stabilizing the inward conformation of the compact structure. This conformational variability provides one explanation for the limited resolution of the single-particle reconstruction. Homogenous refinement focused on a single protomer did not increase the global resolution. This suggests the conformational changes are more complex than simple rigid body rotations of a complete protomer.

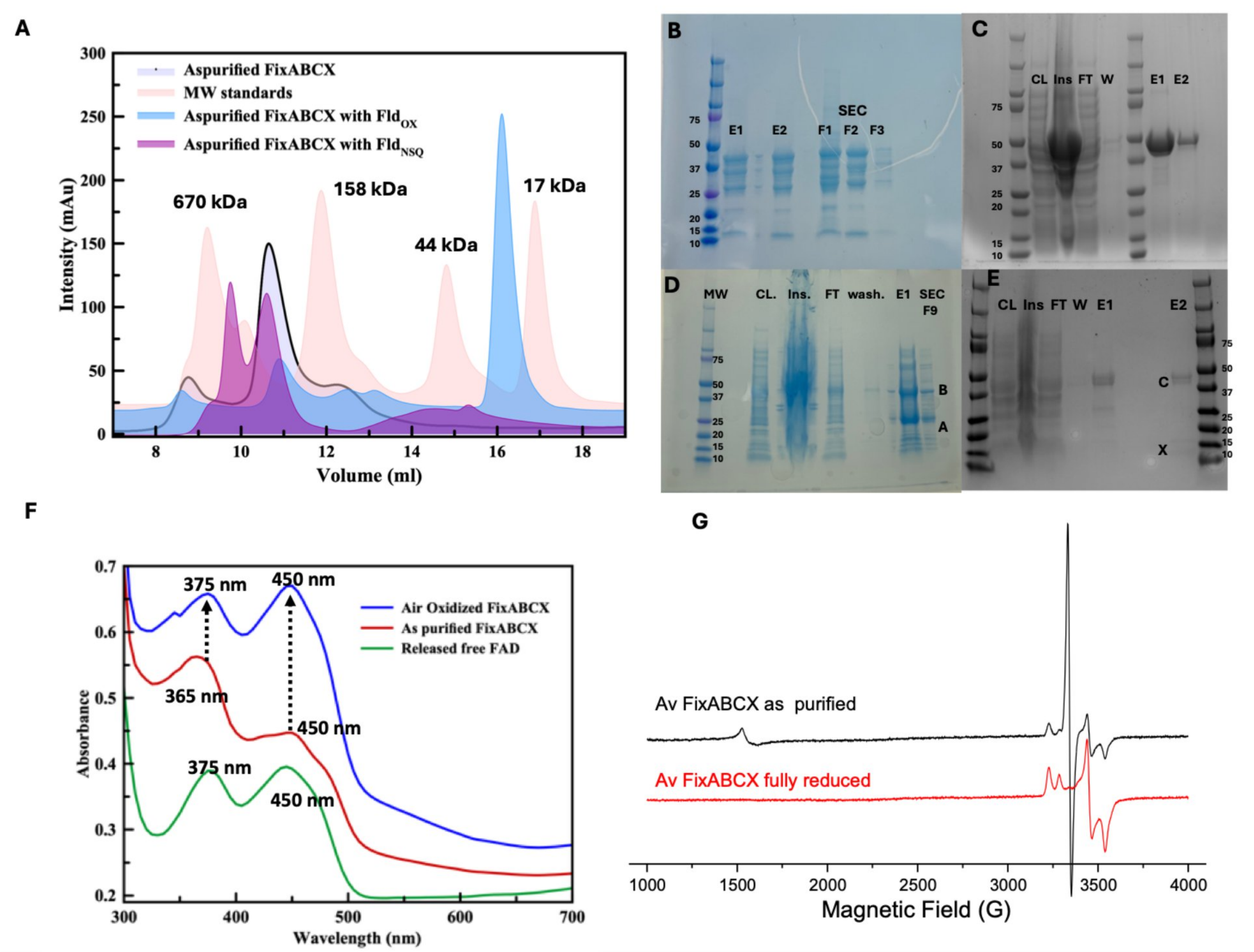

Figure S6: SEC profile, SDS PAGE gels, UV-visible and EPR characterization of *Av*FixABCX. A) SEC profile showing presence of *Av*FixABCX octamer (260 kDa) in as-purified sample (light purple) between the MW standards 670 kDa and 158 kDa; peaks eluting below ~9.5 mL are due to aggregation/ larger MW components. In the light purple chromatogram, a much smaller peak corresponding to the 158 MW standard appears due to the 130 kDa tetramer state of FixABCX. When oxidized Fld$_{OX}$ was mixed (blue), the Fld did not bind and eluted later, around 16 mL, with an MW of ~20 kDa. However, partially reduced Fld$_{NSQ}$ binds to FixABCX (dark purple). B) SDS-PAGE gel of *Av*FixABCX, C) *Av*FixX with MBP tag, D) *Av*FixAB, and E) *Av*FixCX; E1 and E2 stand for eluates, F1, F2, F3 stand for SEC fractions corresponding to the light purple chromatogram, CL- cell lysate, Ins-insoluble fraction, FT-flow through, and W-wash. F) UV-visible spectrum of as-purified *Av*FixABCX (red), air-oxidized *Av*FixABCX (blue) and free FAD released from *Av*FixABCX after acid denaturation of protein (green); partially reduced FAD spectrum intensity increases (red to blue) when air-oxidized; band 2 at 365 nm, shows a red shift to 375 nm is an indication of FAD oxidation. G) X-band EPR spectra of as-purified *Av*FixABCX and NaDT reduced *Av*FixABCX; as-purified *Av*FixABCX shows highly intense isotropic FAD, SQ radical, and two rhombic [4Fe-4S] cluster radical signals. Reduction with NaDT decreases the FAD radical intensity as the paramagnetic SQ state of FAD converts to the diamagnetic HQ state. However, rhombic signal intensity increases as more population of [4Fe-4S] clusters are being reduced.

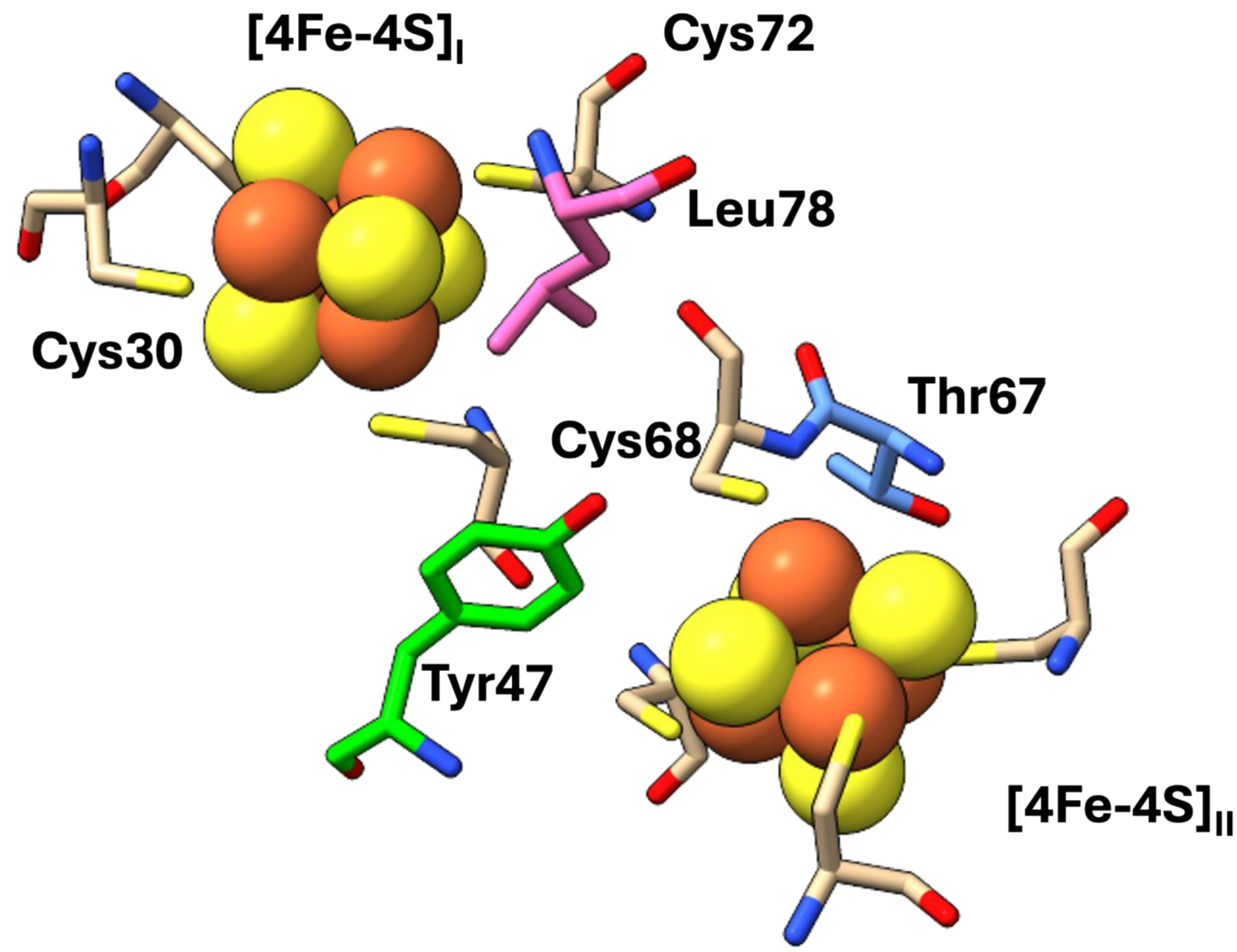

Figure S7: Amino acid arrangement near [4Fe-4S] clusters in *Av*FixX; Conserved Tyr47 and Thr67 only interact with Cys68 in $[4Fe\text{-}4S]_{II}$. This interaction increases the electronegativity of $[4Fe\text{-}4S]_{II}$. This interaction could cause charge distribution in the $[4Fe\text{-}4S]_{II}$ cubane, leading to spin isomers that give different rhombic signals when paramagnetic. In addition, Tyr47 interaction with the Cys68 could increase the electronegativity in the cubane and increase the reduction midpoint potential of $[4Fe\text{-}4S]_{II}$.

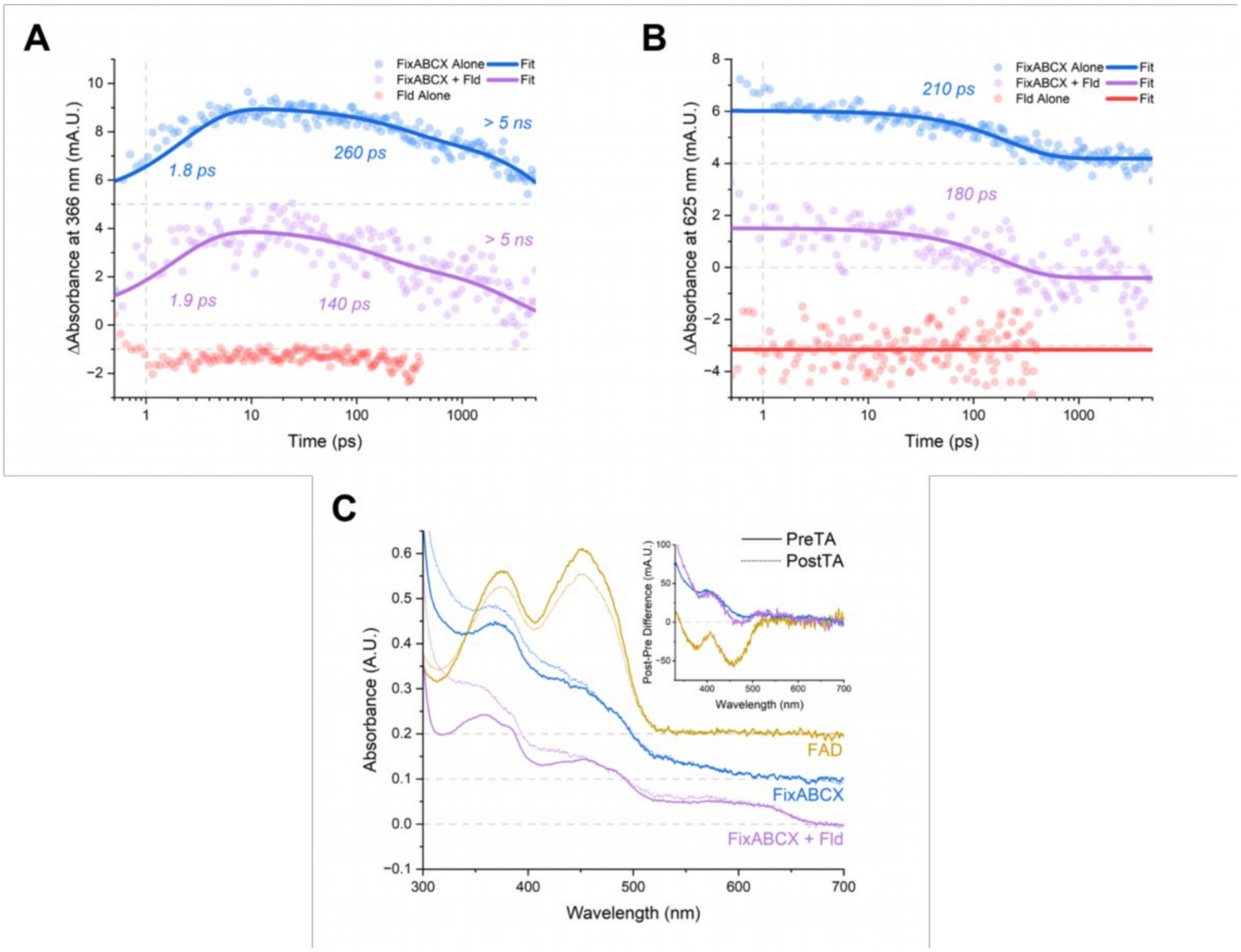

Figure S8. Kinetic traces following the ASQ at 366 nm (A) and the NSQ at 625 nm (B) from the FixABCX alone (*blue*), FixABCX with Fld_{NSQ} (*purple*), and Fld_{NSQ} alone (*red*) samples. Between the two conditions containing FixABCX, there are no differences in the rates for the ASQ at 366 nm nor for the NSQ at 625 nm, showing the presence of Fld_{NSQ} by itself does not impact electron transfer pathways in FixABCX. Trace fitting parameters are located in Table S6. C) Comparison of the pre- and post-TA UV-Visible spectra for the FixABCX alone (*blue*) and FixABCX with Fld_{NSQ} (*purple*) samples and an FAD only control (gold). Photooxidation occurs in both the FixABCX alone and the FixABCX with Fld_{NSQ} after TA spectroscopy, in contrast to free FAD which photoreduces (*inset*). There are broad absorbance increases in the wavelength region < 400 nm after the TA experiment that likely represent some minor protein aggregation that occurs over the course of the experiment and is unrelated to the TA behavior in this report.

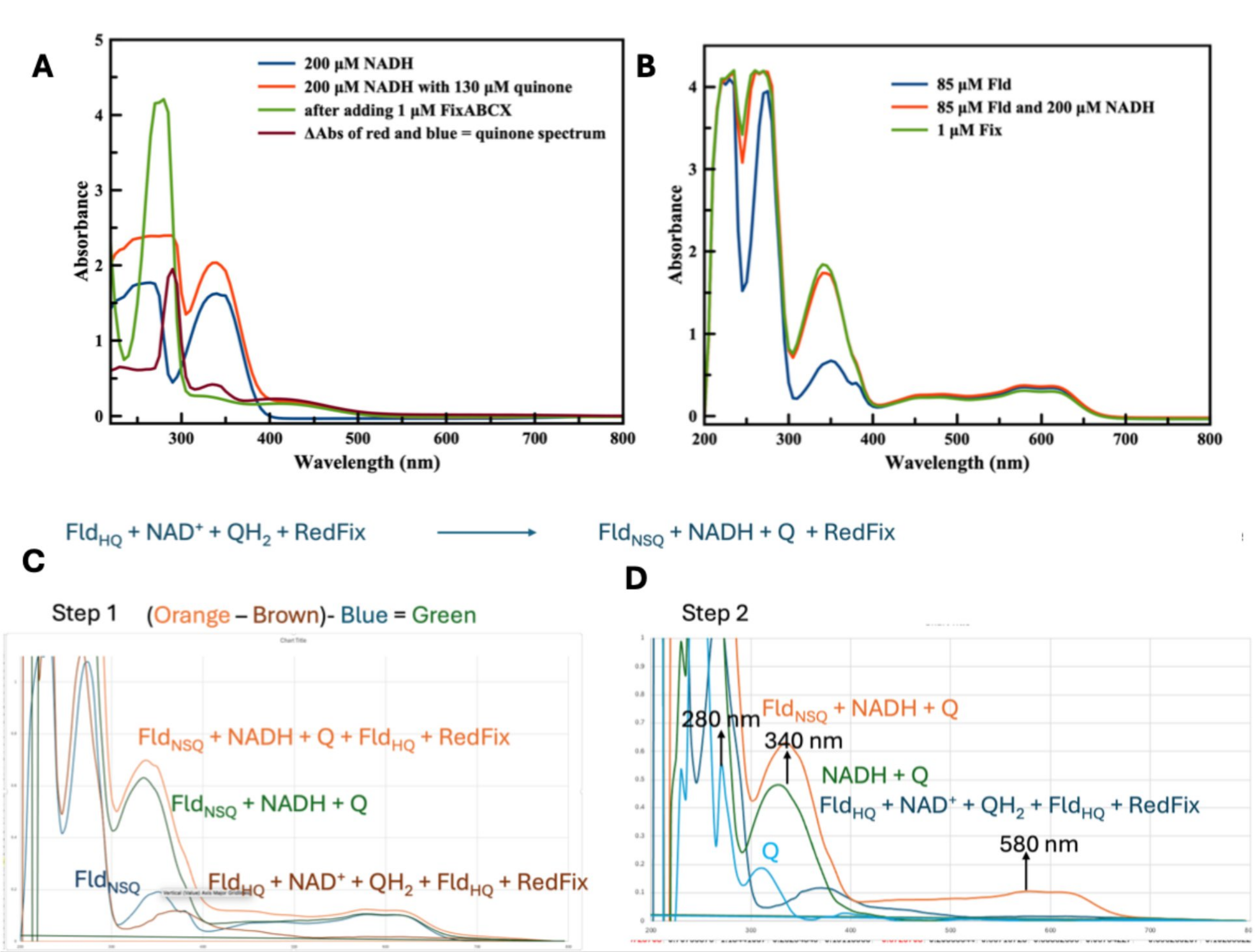

Figure S9:UV-visible spectral change during the steady-state kinetic reactions and the steps of the spectral deconvolution. A) Spectral change during NADH oxidation and quinone reduction in the high-potential exergonic branch; this reaction consumes 130 µM NADH to reduce 130 µM Q in a 1.:1 ratio. B) Lower-potential uphill branch reaction; Fld_{NSQ} spectrum (maximum absorbance at 580 nm) along with NADH absorbance at 340 nm, at wavelengths below 400 nm, protein, NADH, and Q absorb, but at 580 nm only Fld_{NSQ}. C) and D) show spectral deconvolution using the confurcation reaction; step 1 shows the initial (brown) spectrum and the final (orange) spectrum after 10 min reaction. Subtracting brown from orange yields a green spectrum, with only Fld_{NSQ}, Q, and NADH formed. Using the concentration of NSQ formed, the NSQ spectrum of that concentration was subtracted from the green spectrum to get the green spectrum shown in step 2. From this spectrum, the NADH spectrum based on absorbance at 340 nm was subtracted to obtain the light blue spectrum corresponding to Q, which is identical to the Q spectrum shown in red in panel A. Absorbances at 280 nm and 1.5 mM^{-1} cm^{-1} were used to calculate the concentration of Q formed. This reaction stoichiometry between of Fld_{NSQ}:NADH:Q is 2:2:1.

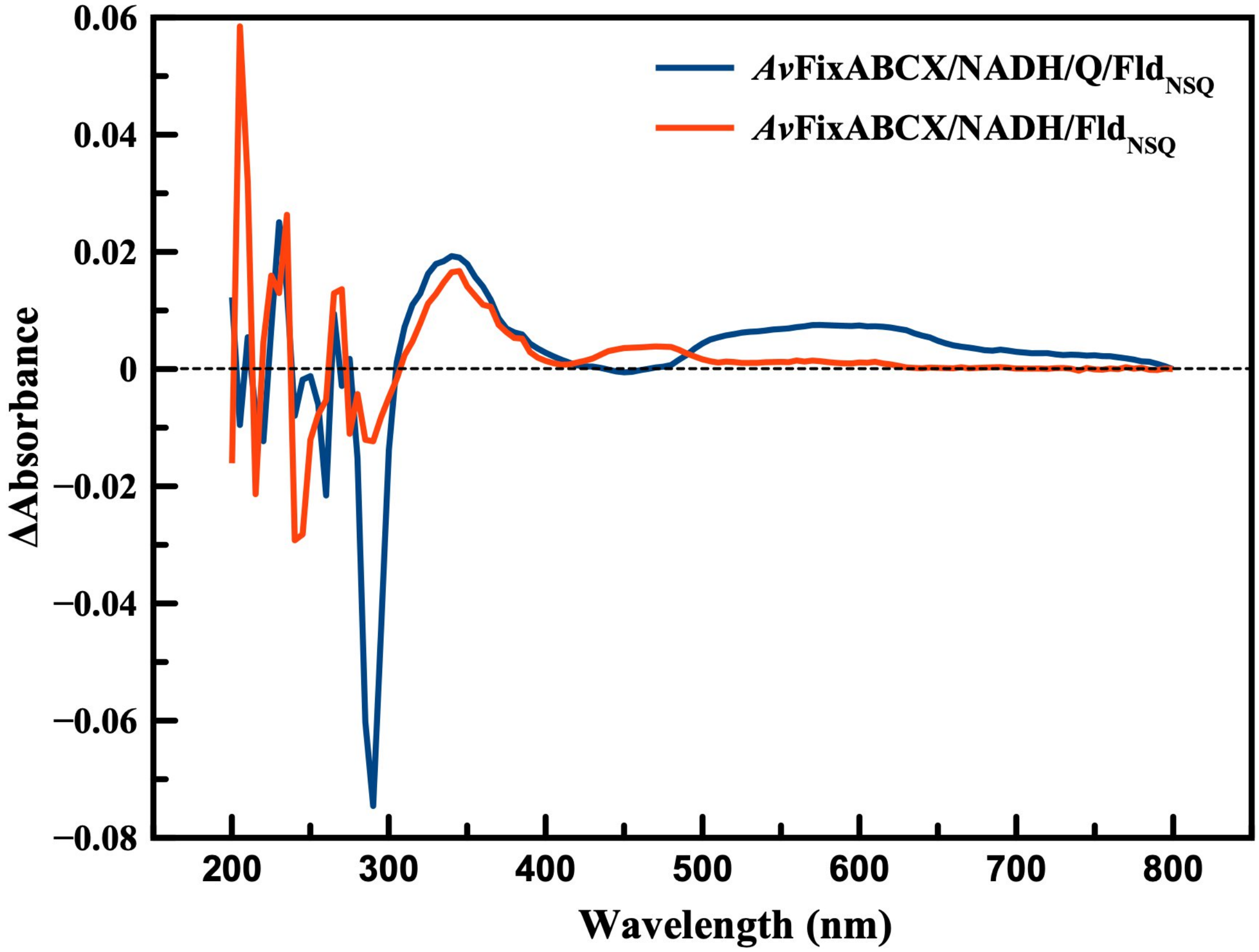

Figure S10: UV-visible spectral change during the bifurcation reaction and the lower-potential half-reaction: The red spectrum shows the spectral change during the lower-potential half-reaction mixture containing *Av*FixABCX, Fld$_{NSQ,}$ and NADH. Absorbance at 340 nm is due to NADH consumption, absorption at 450 nm is due to the change in the absorption of flavins in *Av*FixABCX, and a small absorption change at 580 nm is due to the consumption of Fld$_{NSQ,}$ which is negligible. However, in the blue spectrum, the bifurcation reaction mixture containing *Av*FixABCX, Fld$_{NSQ,}$ NADH, and Q shows a relatively visible spectral change in all components. This indicates the formation of QH_2 at 280 nm, Fld$_{HQ}$ from Fld$_{NSQ}$ at 580 nm, and the consumption of NADH. Here, the absorption at 280 nm includes the change in absorption due to the conversion of Fld$_{NSQ}$ to Fld$_{HQ}$. ~~C~~The change in absorbance due to the conversion of Fld$_{NSQ}$ to Fld$_{HQ}$ is calculated using the extinction coefficient at 280 nm and the concentration of Fld$_{NSQ}$ formed. This absorbance change is subtracted from the total absorbance change at 280 nm, and the resulting absorbance change is due to the formation of QH_2.

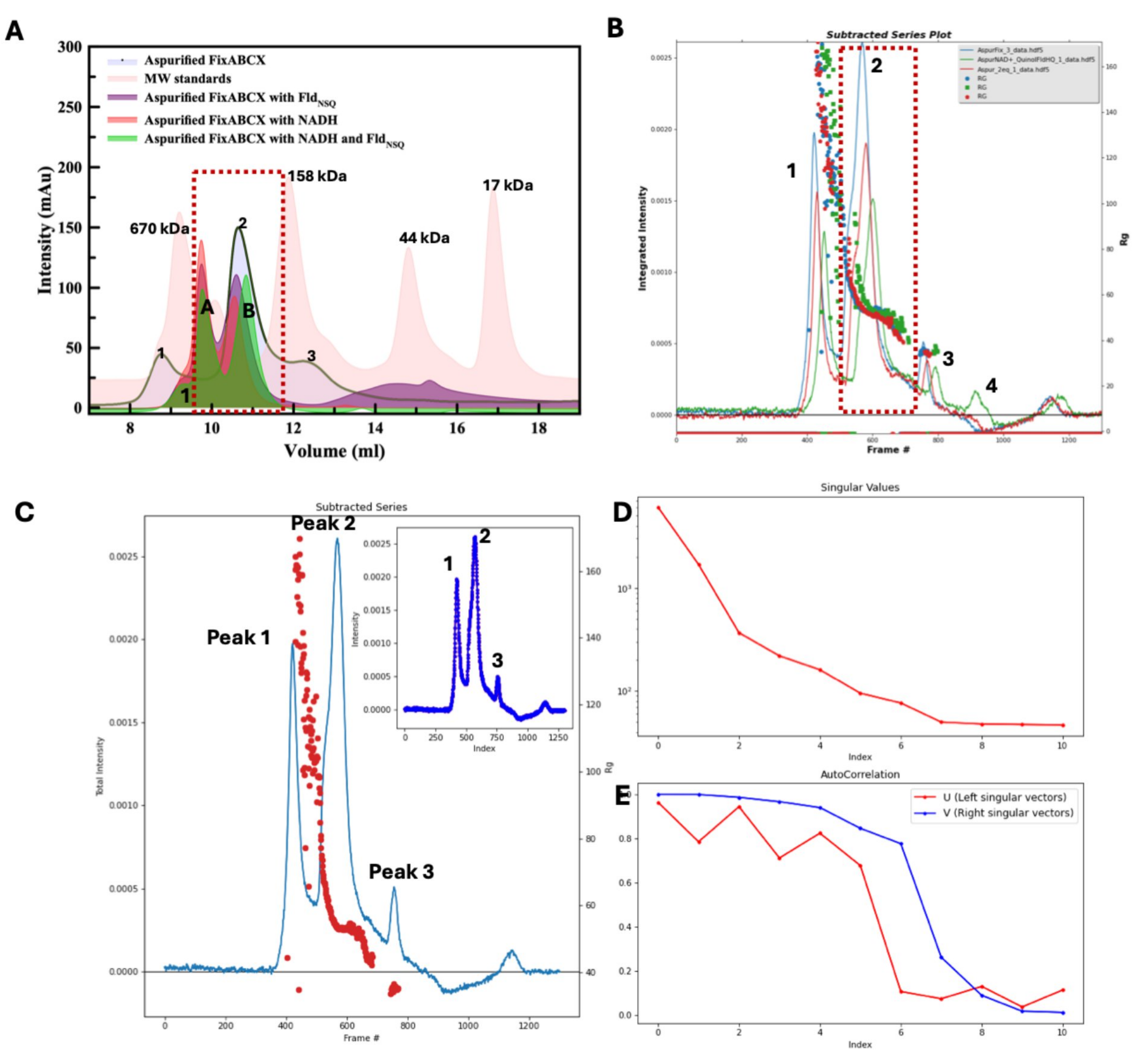

Figure S11: Comparison of SEC chromatogram and SEC-SAXS scattering profiles. A) SEC chromatogram of as-purified *Av*FixABCX (light purple), as-purified *Av*FixABCX with 2 eq NADH (red), as purified *Av*FixABCX with 2 eq *Av*Fld$_{NSQ}$ (dark purple) and as purified *Av*FixABCX with 2 eq NADH and 2 eq *Av*Fld$_{NSQ}$ (dark green); in light purple chromatogram, Peak 1 is due to the higher molecular weight aggregates (elutes below 9.5 ml of void volume (8-9.5 ml) in Superdex200 10/300 size exclusion column), Peak 2 is where the majority of octamer (260 kDa) *Av*FixABCX elutes and peak 3 is corresponding to the tetramer *Av*FixABCX (130 kDa). According to the mass spectrometer analysis, MW standards and the SDS-PAGE gels in Figure S5 (fractions F1, F2 and F3 in Figure S5B) all three peaks contain *Av*FixABCX however in peak 1 it appears as aggregation. B) SEC-SAXS profiles of as purified *Av*FixABCX (blue), as purified *Av*FixABCX with 2 eq NADH (red), and as purified *Av*FixABCX with NADH and *Av*Fld$_{NSQ}$ (green); peak 1 contains randomly distributed particles with varying degrees of MW/R_g values due to the scattering of aggregated protein as observed in Peak1 in panel A. Peak 2 shows particles with different R_g values at the beginning of the peaks and constant R_g value later, the corresponding peak 2 in SEC chromatogram (panel A) in the similar conditions, when NADH or/and *Av*Fld$_{NSQ}$ is/are present two distinguished peaks appear above 9.5 ml and below 12 ml where the octamer of *Av*FixABCX elutes. This indicates the presence of particles with two hydrodynamic radius/shape in

solution; however, the scattering plot is indistinguishable and appears as a single peak (peak 2 in panel B) with a varying R_g value. Peak 3 in panel B for as-purified *Av*FixABCX and as-purified *Av*FixABCX with NADH or/and *Av*Fld_{NSQ} shows a small particle not corresponding to *Av*FixABCX, with R_g of 30 Å, and peak 4 is present only in the sample containing *Av*FixABCX with 2 eq NADH along with 2 eq *Av*$Fld_{NSQ,}$ which could be due to the unbound *Av*$Fld_{NSQ.}$ Scattering profiles of all the other samples are similar to the profiles shown in panel B, depending on the reaction conditions. C, D, and E) Singular value decomposition (SVD) of the entire profile shows the presence of seven scattering components in all the samples tested; SVD of as-purified *Av*FixABCX is shown here.

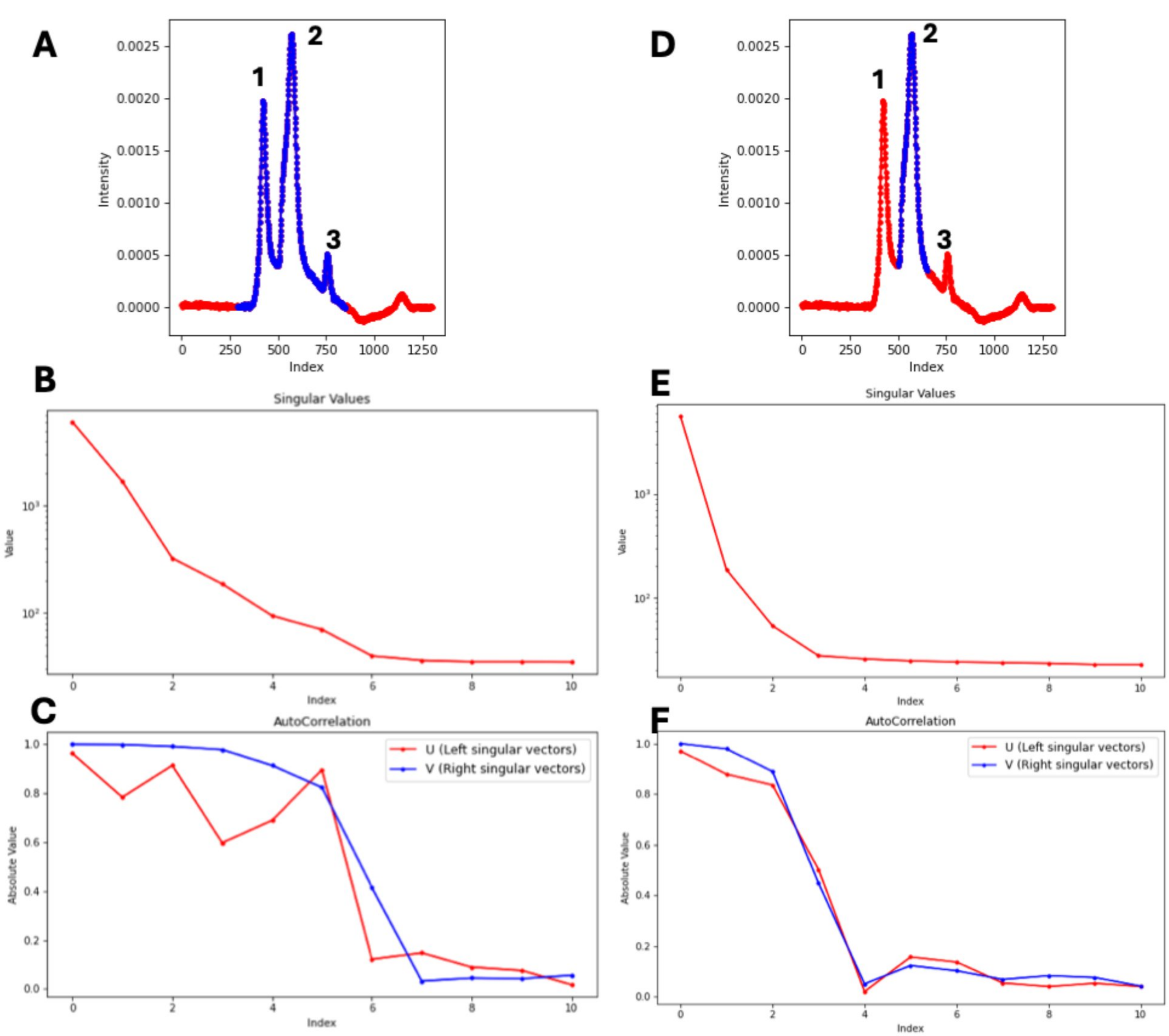

Figure S12: A, B and C) SVD of three overlapping peaks in the as-purified *Av*FixABCX SEC-SAXS profile shows six scattering components. D, E and F) SVD of peak 2 shows three scattering components. When selecting the EFA range to include fewer scattering components, the absolute values of the left and right singular vectors are above 0.6- 0.7. Further, the scattering components with higher singular values in peak 2 are distinguishable. SVD of as-purified, oxidized, and reduced *Av*FixABCX showed similar behavior.

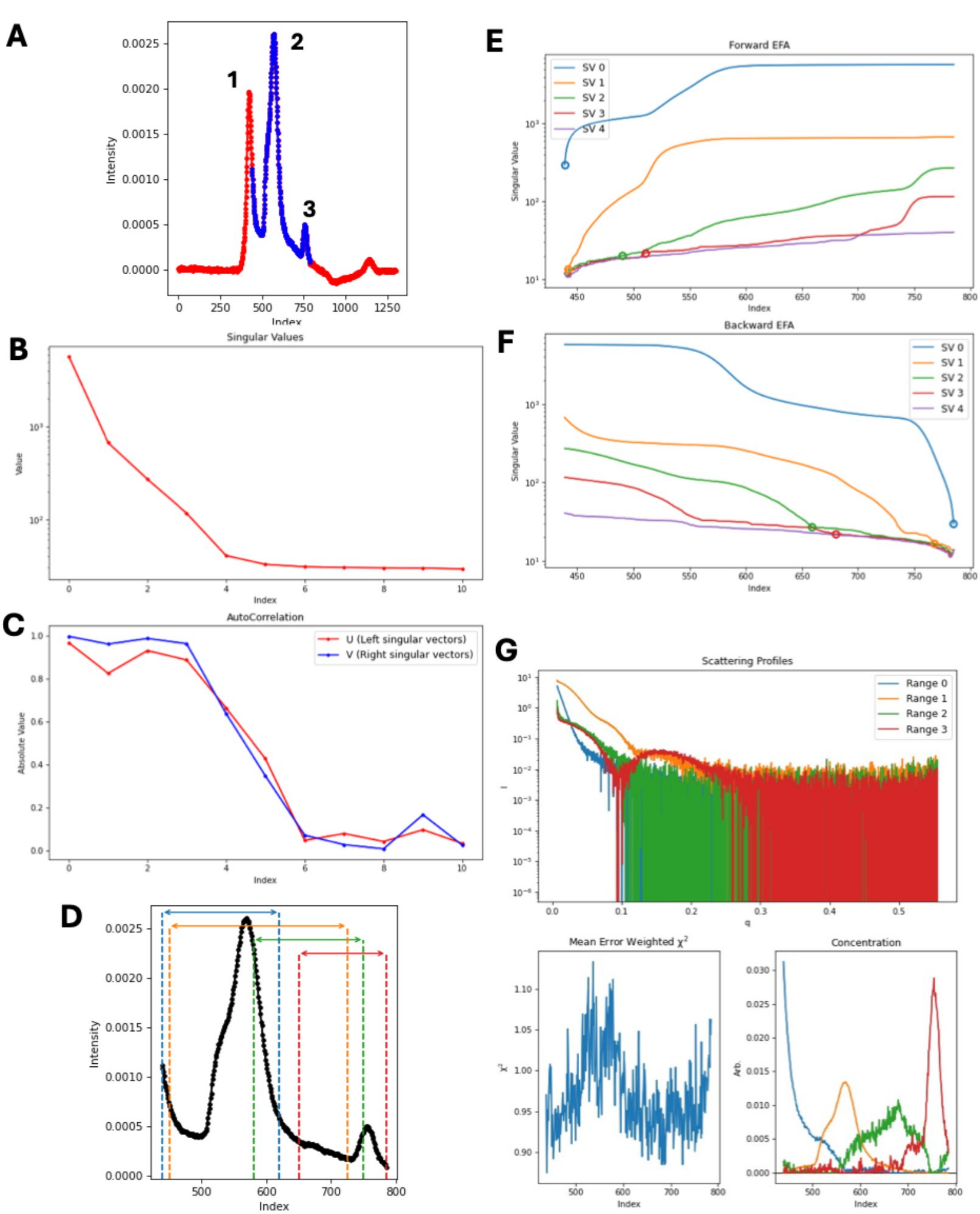

Figure S13: Evolving factor analysis (EFA) of as-purified AvFixABCX sample; A, B and C) EFA range was set to include four singular values, excluding the particles with R_g values above 90 Å as they are randomly distributed in the peak 1. Also, the particles with 90 Å and below are eluting in peak 2; this range allowed the absolute value of the left and right singular vectors to be above or equal to 0.6. E and F) Forward and backward EFA. D and G) Starting and end points of each component in the profile and their

scattering profiles. Yellow peak shows the highest concentration in peak 2 region from 500-700 frames and blue peak ends eluting in peak 2 and green peak start to elute in peak 2. However, the major scattering component does not overlap with either in peak 1 or peak 3 region. χ^2 value of the EFA analysis is between 0.9- 1 indicating a very good fit and the scattering profile of corresponding yellow peak is due to an octamer of *Av*FixABCX. Similarly, EFA of as purified, oxidized and reduced *Av*FixABCX was carried out.

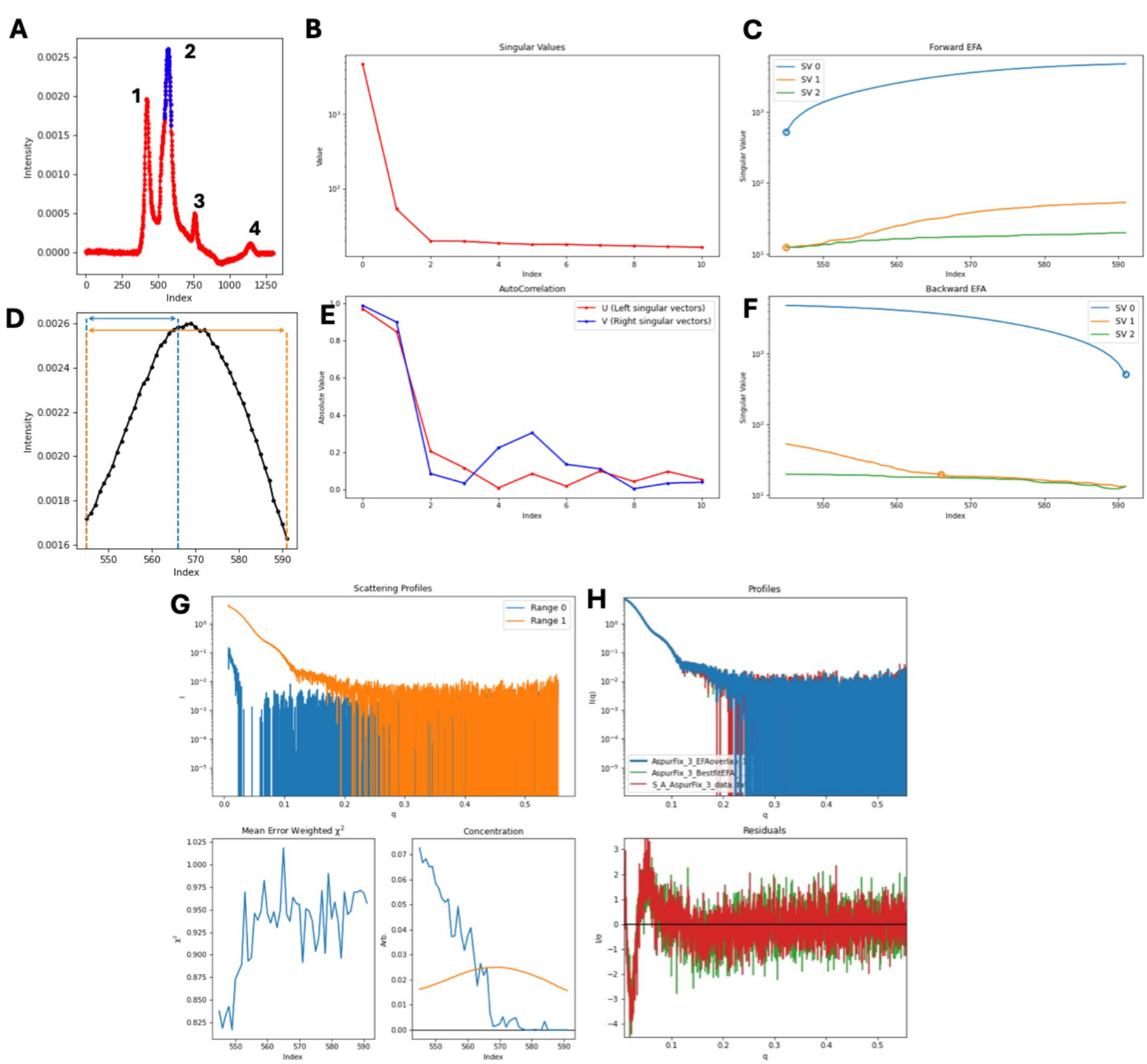

Figure S14: A, B, C, D, E, and F) EFA analysis of the as-purified *Av*FixABCX sample by picking the region with a higher singular value. G) Choosing the two-component region in peak 2 provides the scattering profile with a χ^2 value of 1 in the 560- 590 frames of peak 2. H) The comparison of scattering plots derived from EFA analysis and the direct analysis using the constant R_g value fits well.

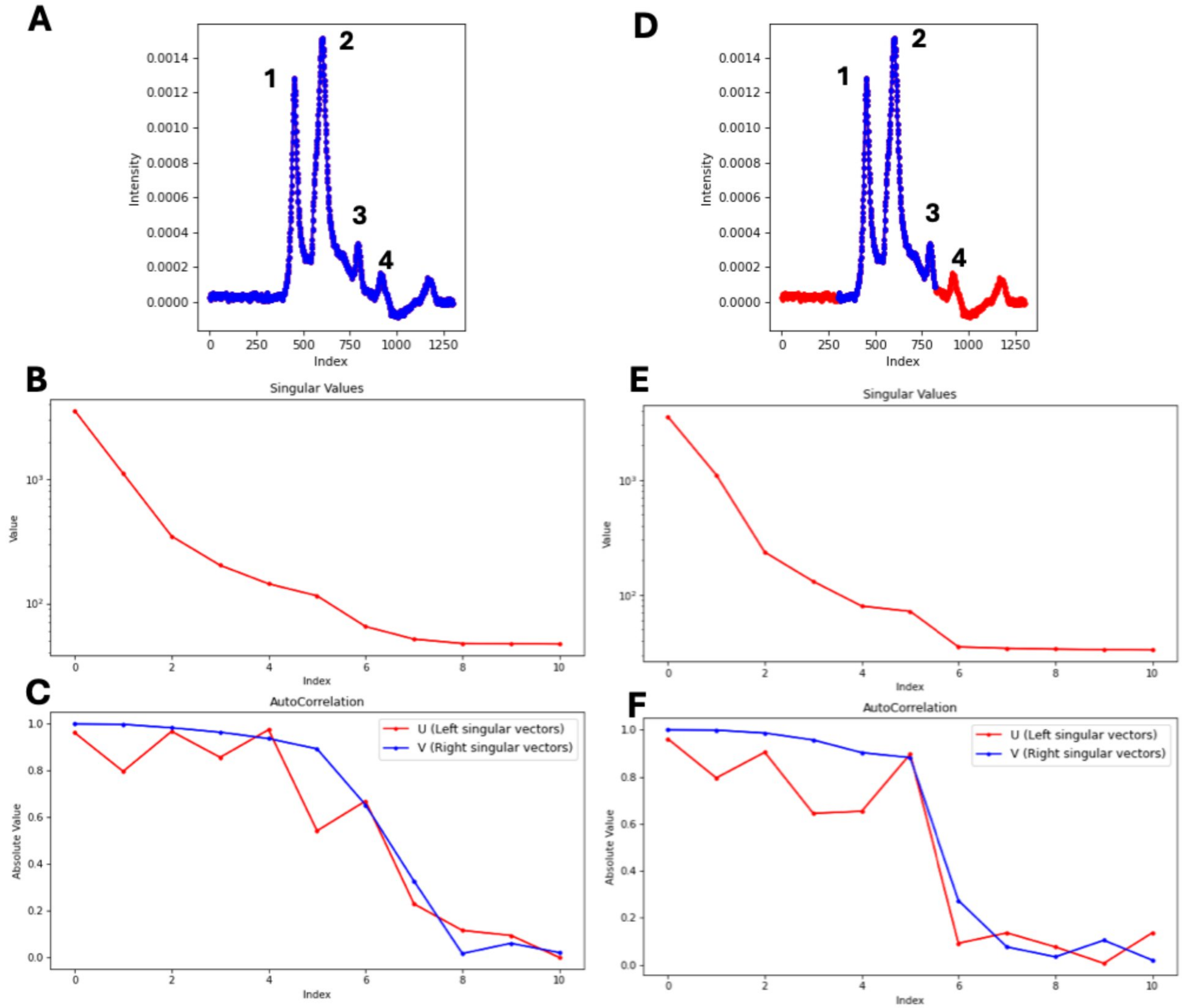

Figure S15: A, B and C) SVD of the entire elution profile in the sample containing as-purified *Av*FixABCX with *Av*Fld$_{NSQ}$, NADH and Q shows seven scattering components. Additional peak 4 in these samples could be due to the unbound access *Av*Fld$_{NSQ.}$ D, E and F) SVD of three overlapping peaks in the as-purified *Av*FixABCX with *Av*Fld$_{NSQ}$, NADH and Q shows six scattering components.

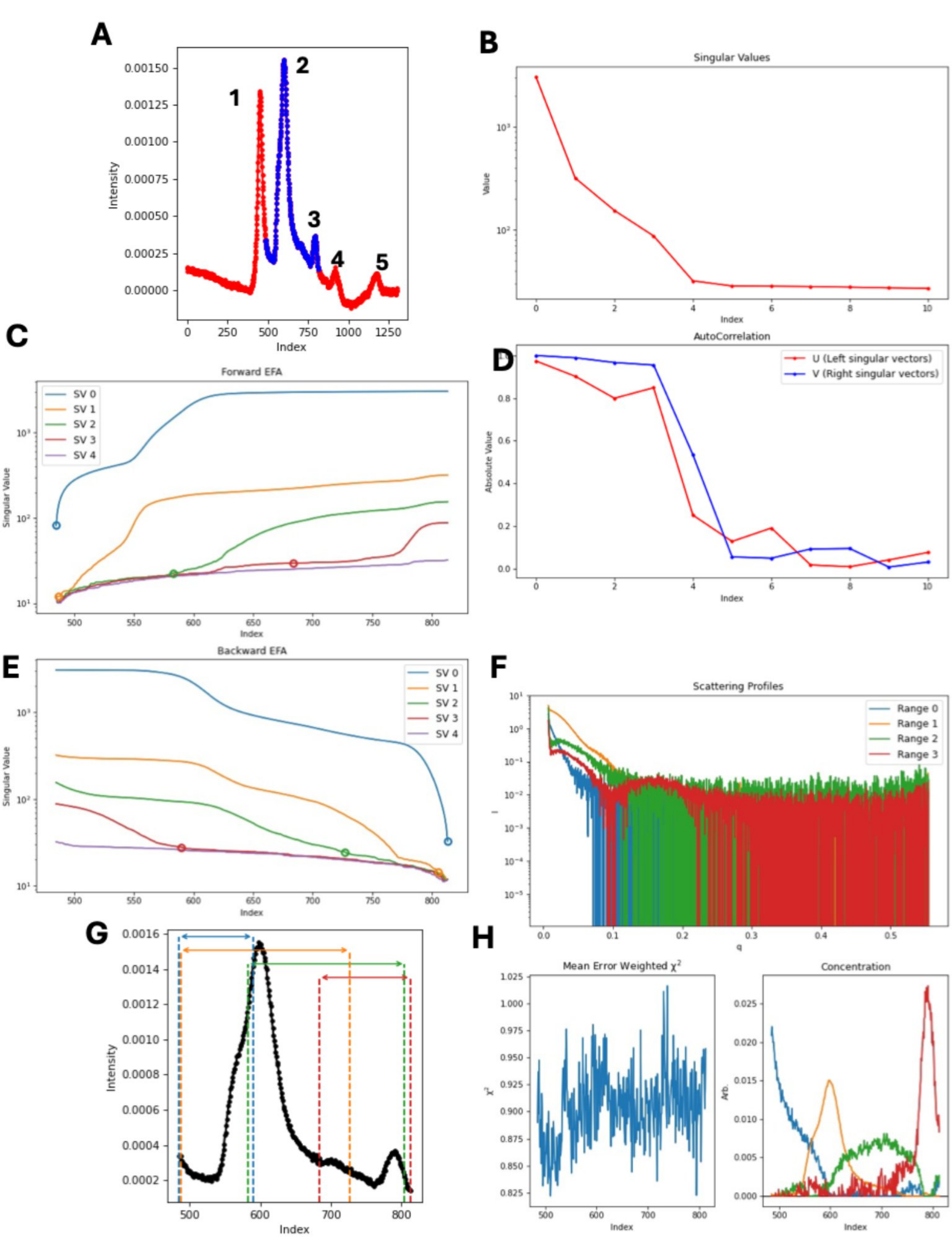

Figure S16: Evolving factor analysis (EFA) of as-purified AvFixABCX with AvFldNSQ, NADH, and Q; A, B, and D) EFA range was set to include four singular value, excluding the particles with R_g values

above 90 Å as they are randomly distributed in the peak 1. Also, the particles with 90 Å and below are eluting in peak 2; this range allowed the absolute value of the left and right singular vectors to be above or equal to 0.6. C and E) Forward and backward EFA. G and F) Starting and end points of each component in the profile and their scattering profiles. Yellow peak shows the highest concentration in peak 2 region from 500-700 frames and blue peak ends eluting in peak 2 and green peak start to elute in peak 2. However, the major scattering component does not overlap with either in peak 1 or peak 3 region. χ^2 value of the EFA analysis is between 0.85- 1 indicating a very good fit and the scattering profile of corresponding yellow peak is due to an octamer of *Av*Fld bound *Av*FixABCX. Similarly EFA analysis for samples containing as-purified *Av*FixABCX with AvFld$_{HQ}$, NAD^+, and QH_2 was carried out.

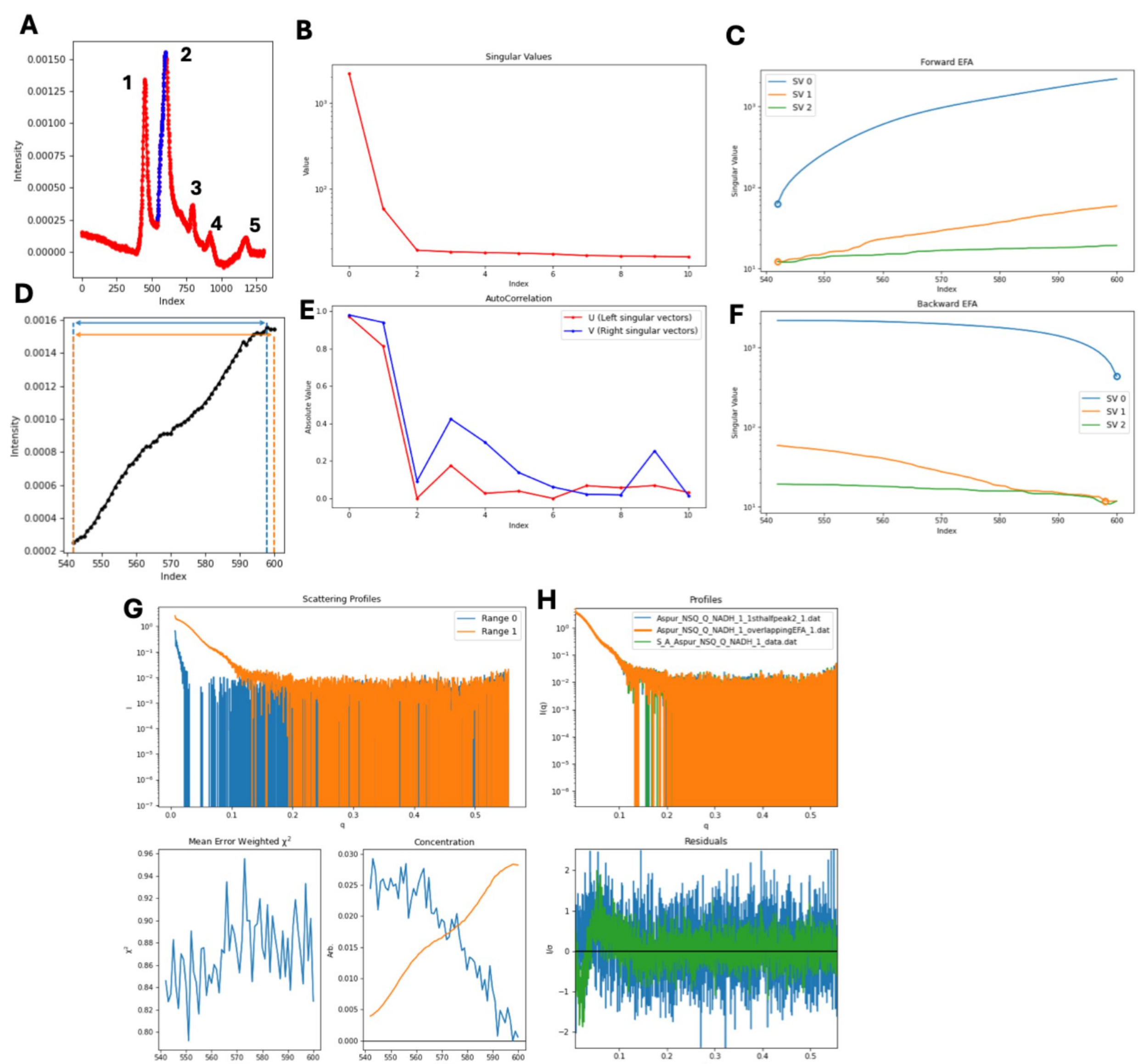

Figure S17: A, B, C, D, E, and F) EFA analysis of the as-purified *Av*FixABCX with AvFld$_{NSQ}$, NADH, and Q by picking the region with a higher singular value. G) Choosing the two-component region in peak 2 provides the scattering profile with a χ^2 value closer to 1 in the 560- 590 frames of peak 2. H) The comparison of scattering plots derived from EFA analysis and the direct analysis using the constant R_g

value fits well. Similarly EFA analysis for samples containing as-purified *Av*FixABCX with *Av*Fld_{HQ}, NAD^{+}, and QH_2 was carried out.

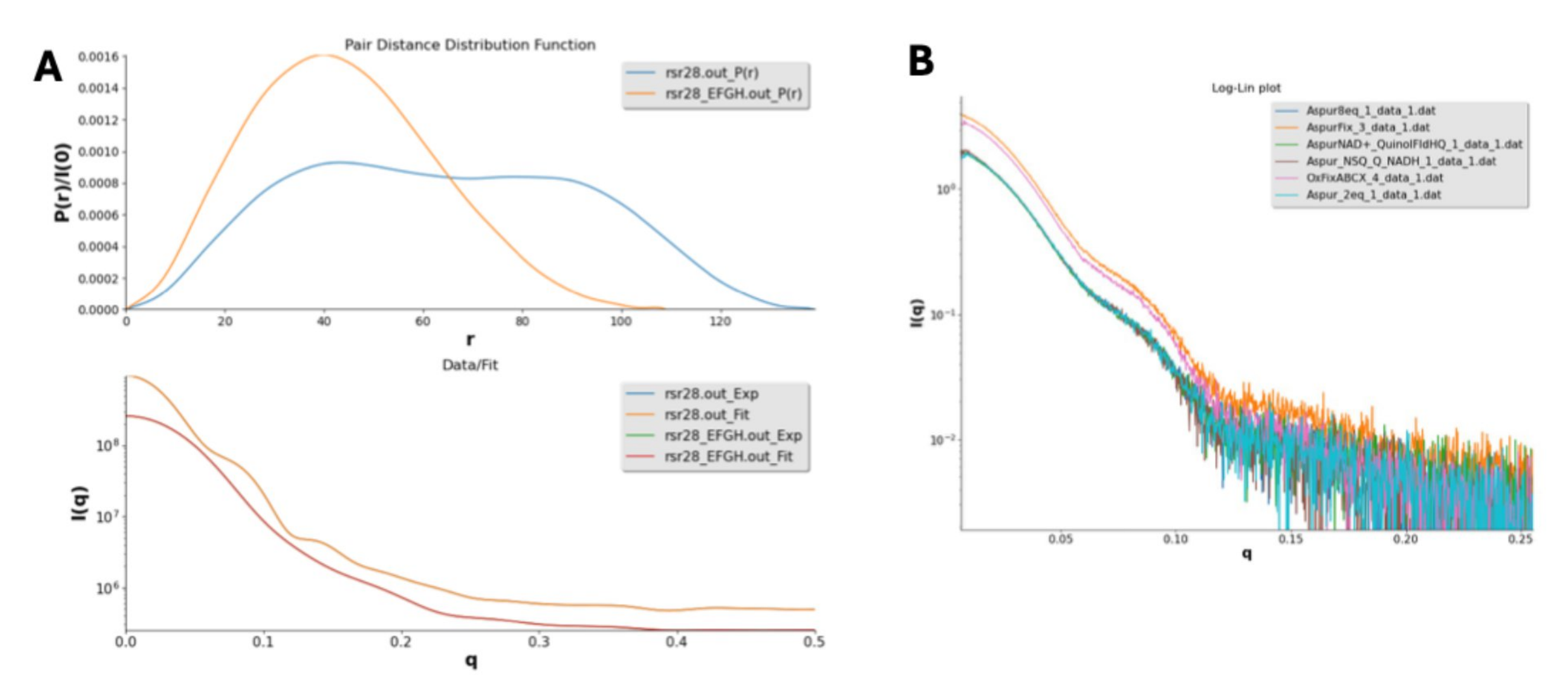

Figure S18: A) P(r) plot of the *Av*FixABCX cryo-EM structure; SAXS curves for octamer and tetramer were generated using Crysol in the ATSAS package. The octamer is in blue (rsr28), and the tetramer is in orange (rsr28-EFGH). Log-Lin plot of the octamer and tetramer is given in the panel below in A; the octamer shows two curves below q = 0.1, whereas the tetramer shows 1 curve. B) Log-Lin plots of all the SAXS curves generated using the EFA for the octamers present in the samples tested in this study. All the curves resemble the Log-Lin plot of *Av*FixABCX octame.

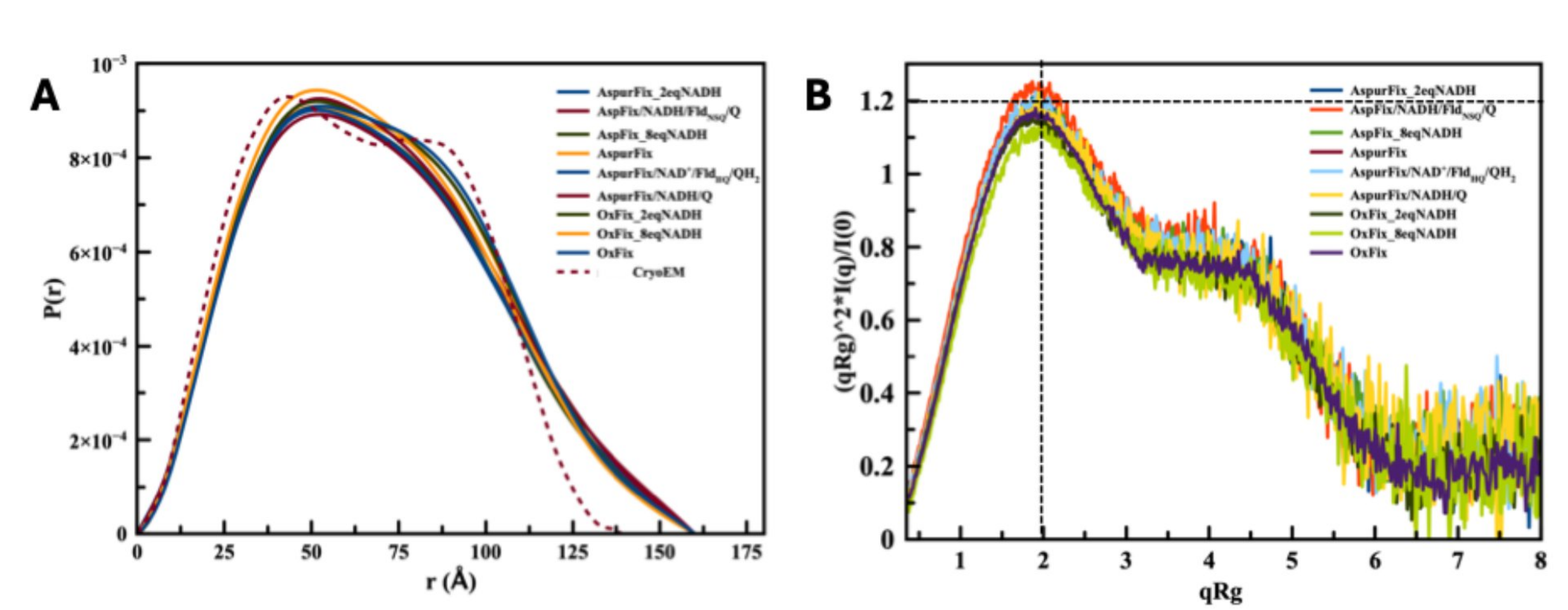

Figure S19: P(r) function and dimensionless Kratky plot. A)P(r) function of all the octamers in the tested SEC-SAXS samples tabulated in Tables S7 and S8. B) Dimensionless Kratky plot shows two peaks for all the octamers, and above qR_g =2, the curve comes to zero, showing a stable, folded protein in solution.

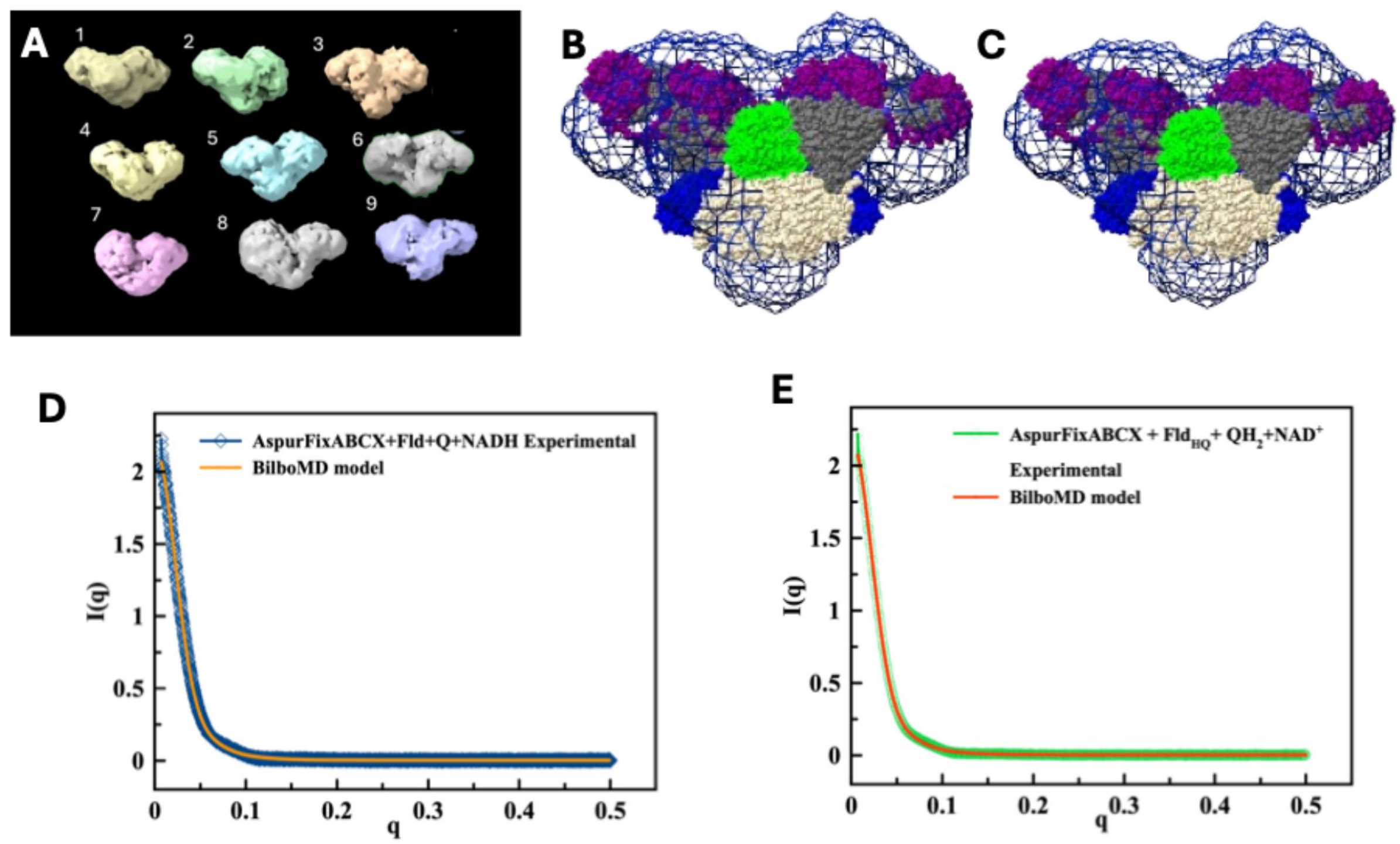

Figure S20: DENSS maps generated for all the SAXS curves using RAW. A);1)As purified FixABCX with 1eq Q and 2eq NADH, 2) As purified Fix with 1eq Q, 2eq Fld_{NSQ} and 2eq NADH, 3) NaDT reduced FixABCX, QH_2, Fld_{HQ}, and NAD^+.,4) air oxidized FixABCX, 5) air oxidized FixABCX with 2eq NADH, 6) air oxidized FixABCX with 8 eq NADH, 7) As purified FixABCX 8) As purified FixABCX with 2 eq NADH and 9) As purified FixABCX with 8 eq NADH. B) and C) Supperimposition of BilboMD models generated for; 2) As purified FixABCX with 1 eq Q, 2 eq Fld_{NSQ} and 2 eq NADH, and 3) NaDT reduced as purified FixABCX, QH_2, Fld_{HQ}, and NAD^+; with the DENSS map generated for the SAXS scattering plots in RAW. D) and C) Linear-Linear plot of experimental and Model SAXS scattering plot showing the best fit.

Note: B) and C) BilboMD models of extended conformer generated for both confurcation and bifurcation reactions where *Av*Fld (green) is bound to *Av*FixABCX in the FixA subunits; best fit of the extended domain II in the envelope or DENSS map validate the SAXS scattering plot of the model and experimental data.

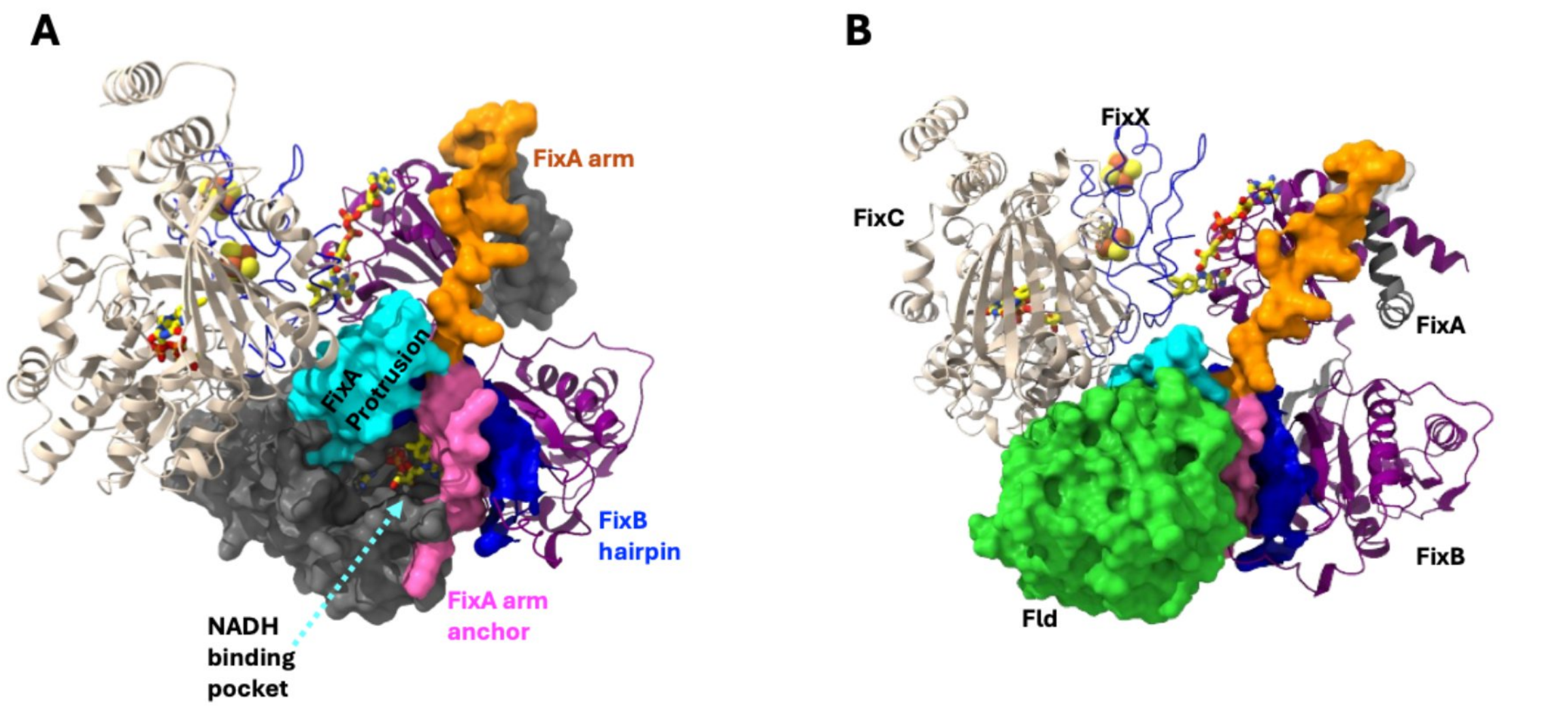

Figure S21: Amino acid segments surrounding the NADH binding pocket near the Bf-FAD site of *Av*FixABCX, which is known to play an important role in domain II movement during catalysis, A) FixA protrusion in cyan and FixA arm anchor in pink, FixA arm is in the second loop that stabilizes domain II (orange) and FixB hairpin in blue. B) Fld (green) interaction with FixA arm anchor and FixA protrusion. In the absence of NADH/Fld, FixA arm is in the second loop that stabilizes domain II, and FixB hairpin interacts with FixA protrusion. NADH binding will displace the FixA anchor arm thereby disengages a subsequent interactions between FixB hairpin with FixA protrusion. Subsequently, FixA arm pull the domain II towards the NADH/Fld binding site during EB[49]. NADH binding pocket is where Fld/Fd binds during EB[50]. These amino acid segments could play an essential role in the conformational change as reported in the EtfAB-Bcd open and closed conformer crystal structures[49].

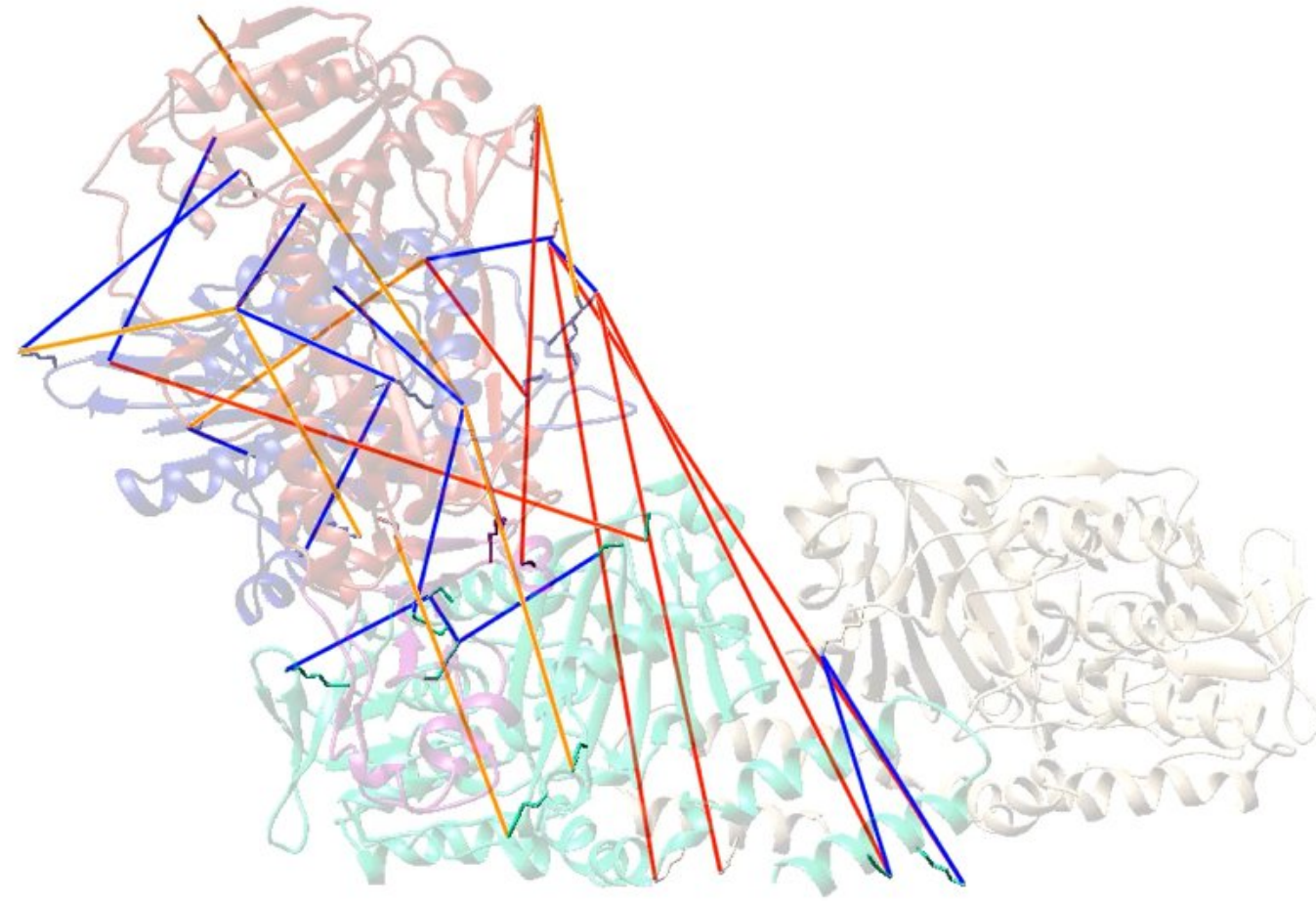

Movie S2. Long-range BS3 crosslinks detected between domain II (FixB) and FixC, FixA and FixX in the presence and absence of NADH.

BS3 crosslinker has a linker arm that is 11.4 Å long when fully extended, allowing Cα atoms of crosslinked lysine residues to be up to ~26-30 Å apart[57]. In the movie, blue, orange and red crosslinks represent distance up to 30 Å, between 30-50 Å and over 50 Å, respectively. FixA in blue, FixB in red, $FixC_1$ in green, FixX in magenta and $FixC_2$ in tan. Presence of crosslinks within 30 Å as well as the long-range ones suggests: structural change within the octamer and/or presence of multiple conformers in the solution. Change in dynamics and/or shift in conformational ensemble equilibrium would be sensitive to complex stoichiometry, cofactors oxidation state and ligand binding.

References

(1) Ledbetter, R. N.; Garcia Costas, A. M.; Lubner, C. E.; Mulder, D. W.; Tokmina-Lukaszewska, M.; Artz, J. H.; Patterson, A.; Magnuson, T. S.; Jay, Z. J.; Duan, H. D.; et al. The Electron Bifurcating FixABCX Protein Complex from Azotobacter vinelandii: Generation of Low-Potential Reducing Equivalents for Nitrogenase Catalysis. *Biochemistry* **2017**, *56* (32), 4177-4190. DOI: 10.1021/acs.biochem.7b00389.
(2) Aliverti, A.; Curti, B.; Vanoni, M. A. Identifying and quantitating FAD and FMN in simple and in iron-sulfur-containing flavoproteins. *Methods Mol Biol* **1999**, *131*, 9-23. DOI: 10.1385/1-59259-266-x:9 From NLM.
(3) fish, W. W. [27] Rapid colorimetric micromethod for the quantitation of complexed iron in biological samples. In *Methods in Enzymology*, Vol. 158; Academic Press, 1988; pp 357-364.
(4) Freibert, S.-A.; Weiler, B. D.; Bill, E.; Pierik, A. J.; Mühlenhoff, U.; Lill, R. Chapter Seven - Biochemical Reconstitution and Spectroscopic Analysis of Iron–Sulfur Proteins. In *Methods in Enzymology*, David, S. S. Ed.; Vol. 599; Academic Press, 2018; pp 197-226.
(5) Gauvin, C. C.; Findlay, J. L.; Hophan-Nichols, C.; Lawrence, C. M. A cost-effective 200 kV cryo-EM core facility for high resolution single particle analysis. *Methods Microsc* **2026**, *3* (1), 55-70. DOI: 10.1515/mim-2025-0039 From NLM.
(6) Bouvette, J.; Huang, Q.; Riccio, A. A.; Copeland, W. C.; Bartesaghi, A.; Borgnia, M. J. Automated systematic evaluation of cryo-EM specimens with SmartScope. *Elife* **2022**, *11*. DOI: 10.7554/eLife.80047 From NLM Medline.
(7) Mastronarde, D. N. Automated electron microscope tomography using robust prediction of specimen movements. *J Struct Biol* **2005**, *152* (1), 36-51. DOI: 10.1016/j.jsb.2005.07.007 From NLM Medline.
(8) Punjani, A.; Fleet, D. J. 3D variability analysis: Resolving continuous flexibility and discrete heterogeneity from single particle cryo-EM. *J Struct Biol* **2021**, *213* (2), 107702. DOI: 10.1016/j.jsb.2021.107702 From NLM Medline.
(9) Punjani, A.; Rubinstein, J. L.; Fleet, D. J.; Brubaker, M. A. cryoSPARC: algorithms for rapid unsupervised cryo-EM structure determination. *Nat Methods* **2017**, *14* (3), 290-296. DOI: 10.1038/nmeth.4169 From NLM Medline.
(10) Feng, X.; Schut, G. J.; Lipscomb, G. L.; Li, H.; Adams, M. W. W. Cryoelectron microscopy structure and mechanism of the membrane-associated electron-bifurcating flavoprotein Fix/EtfABCX. *Proc Natl Acad Sci U S A* **2021**, *118* (2). DOI: 10.1073/pnas.2016978118 From NLM Medline.
(11) Tan, Y. Z.; Baldwin, P. R.; Davis, J. H.; Williamson, J. R.; Potter, C. S.; Carragher, B.; Lyumkis, D. Addressing preferred specimen orientation in single-particle cryo-EM through tilting. *Nat Methods* **2017**, *14* (8), 793-796. DOI: 10.1038/nmeth.4347 From NLM Medline.
(12) Baldwin, P. R.; Lyumkis, D. Non-uniformity of projection distributions attenuates resolution in Cryo-EM. *Prog Biophys Mol Biol* **2020**, *150*, 160-183. DOI: 10.1016/j.pbiomolbio.2019.09.002 From NLM Medline.
(13) Baldwin, P. R.; Lyumkis, D. Tools for visualizing and analyzing Fourier space sampling in Cryo-EM. *Prog Biophys Mol Biol* **2021**, *160*, 53-65. DOI: 10.1016/j.pbiomolbio.2020.06.003 From NLM Medline.
(14) Abramson, J.; Adler, J.; Dunger, J.; Evans, R.; Green, T.; Pritzel, A.; Ronneberger, O.; Willmore, L.; Ballard, A. J.; Bambrick, J.; et al. Accurate structure prediction of biomolecular

interactions with AlphaFold 3. *Nature* **2024**, *630* (8016), 493-500. DOI: 10.1038/s41586-024-07487-w From NLM Medline.
(15) Terwilliger, T. C.; Sobolev, O. V.; Afonine, P. V.; Adams, P. D. Automated map sharpening by maximization of detail and connectivity. *Acta Crystallogr D Struct Biol* **2018**, *74* (Pt 6), 545-559. DOI: 10.1107/S2059798318004655 From NLM Medline.
(16) Afonine, P. V.; Headd, J. J.; Terwilliger, T. C.; Adam, P. D. New tool: phenix.real_space_refine. . *Computational Crystallography Newsletter* **2013**, *4*, 43-44.
(17) Afonine, P. V.; Poon, B. K.; Read, R. J.; Sobolev, O. V.; Terwilliger, T. C.; Urzhumtsev, A.; Adams, P. D. Real-space refinement in PHENIX for cryo-EM and crystallography. *Acta Crystallogr D Struct Biol* **2018**, *74* (Pt 6), 531-544. DOI: 10.1107/S2059798318006551 From NLM Medline.
(18) Painter, J.; Merritt, E. A. Optimal description of a protein structure in terms of multiple groups undergoing TLS motion. *Acta Crystallogr D Biol Crystallogr* **2006**, *62* (Pt 4), 439-450. DOI: 10.1107/S0907444906005270 From NLM Medline.
(19) Emsley, P.; Lohkamp, B.; Scott, W. G.; Cowtan, K. Features and development of Coot. *Acta Crystallogr D Biol Crystallogr* **2010**, *66* (Pt 4), 486-501. DOI: 10.1107/S0907444910007493 From NLM Medline.
(20) Liebschner, D.; Afonine, P. V.; Baker, M. L.; Bunkoczi, G.; Chen, V. B.; Croll, T. I.; Hintze, B.; Hung, L. W.; Jain, S.; McCoy, A. J.; et al. Macromolecular structure determination using X-rays, neutrons and electrons: recent developments in Phenix. *Acta Crystallogr D Struct Biol* **2019**, *75* (Pt 10), 861-877. DOI: 10.1107/S2059798319011471 From NLM Medline.
(21) *The PyMOL Molecular Graphics System*; Schröedinger LLC: 2003. The Pymol Molecular Graphics System, Version 11.2r3pre, Schrodinger, LLC. (accessed.
(22) Meng, E. C.; Goddard, T. D.; Pettersen, E. F.; Couch, G. S.; Pearson, Z. J.; Morris, J. H.; Ferrin, T. E. UCSF ChimeraX: Tools for structure building and analysis. *Protein Sci* **2023**, *32* (11), e4792. DOI: 10.1002/pro.4792 From NLM Medline.
(23) Pettersen, E. F.; Goddard, T. D.; Huang, C. C.; Meng, E. C.; Couch, G. S.; Croll, T. I.; Morris, J. H.; Ferrin, T. E. UCSF ChimeraX: Structure visualization for researchers, educators, and developers. *Protein Sci* **2021**, *30* (1), 70-82. DOI: 10.1002/pro.3943 From NLM Medline.
(24) Crofts, A. R.; Rose, S. Marcus treatment of endergonic reactions: A commentary. *Biochimica et Biophysica Acta (BBA) - Bioenergetics* **2007**, *1767* (10), 1228-1232. DOI: https://doi.org/10.1016/j.bbabio.2007.06.006.
(25) Miller, A. F.; Duan, H. D.; Varner, T. A.; Mohamed Raseek, N. Reduction midpoint potentials of bifurcating electron transfer flavoproteins. *Methods in enzymology* **2019**, *620*, 365-398. DOI: 10.1016/bs.mie.2019.03.017 From NLM.
(26) Seefeldt, L. C.; Ensign, S. A. A continuous, spectrophotometric activity assay for nitrogenase using the reductant titanium(III) citrate. *Anal Biochem* **1994**, *221* (2), 379-386. DOI: 10.1006/abio.1994.1429 From NLM.
(27) Hopkins, J. B.; Gillilan, R. E.; Skou, S. BioXTAS RAW: improvements to a free open-source program for small-angle X-ray scattering data reduction and analysis. *J Appl Crystallogr* **2017**, *50* (Pt 5), 1545-1553. DOI: 10.1107/s1600576717011438 From NLM.
(28) Acerbo, A. S.; Cook, M. J.; Gillilan, R. E. Upgrade of MacCHESS facility for X-ray scattering of biological macromolecules in solution. *J Synchrotron Radiat* **2015**, *22* (1), 180-186. DOI: 10.1107/s1600577514020360 From NLM.

(29) Skou, S.; Gillilan, R. E.; Ando, N. Synchrotron-based small-angle X-ray scattering of proteins in solution. *Nat Protoc* **2014**, *9* (7), 1727-1739. DOI: 10.1038/nprot.2014.116 From NLM.
(30) Hopkins, J. B. BioXTAS RAW 2: new developments for a free open-source program for small-angle scattering data reduction and analysis. *J Appl Crystallogr* **2024**, *57* (Pt 1), 194-208. DOI: 10.1107/s1600576723011019 From NLM.
(31) Brookes, E.; Vachette, P.; Rocco, M.; Pérez, J. US-SOMO HPLC-SAXS module: dealing with capillary fouling and extraction of pure component patterns from poorly resolved SEC-SAXS data. *J Appl Crystallogr* **2016**, *49* (Pt 5), 1827-1841. DOI: 10.1107/s1600576716011201 From NLM.
(32) Meisburger, S. P.; Taylor, A. B.; Khan, C. A.; Zhang, S.; Fitzpatrick, P. F.; Ando, N. Domain Movements upon Activation of Phenylalanine Hydroxylase Characterized by Crystallography and Chromatography-Coupled Small-Angle X-ray Scattering. *J Am Chem Soc* **2016**, *138* (20), 6506-6516. DOI: 10.1021/jacs.6b01563 From NLM.
(33) Svergun, D. I. Determination of the regularization parameter in indirect-transform methods using perceptual criteria. *Journal of Applied Crystallography* **1992**, *25* (4), 495-503. DOI: https://doi.org/10.1107/S0021889892001663 (acccessed 2025/10/13).
(34) Grant, T. D. Ab initio electron density determination directly from solution scattering data. *Nat Methods* **2018**, *15* (3), 191-193. DOI: 10.1038/nmeth.4581 From NLM.
(35) Kikhney, A. G.; Svergun, D. I. A practical guide to small angle X-ray scattering (SAXS) of flexible and intrinsically disordered proteins. *FEBS Letters* **2015**, *589* (19, Part A), 2570-2577. DOI: https://doi.org/10.1016/j.febslet.2015.08.027.
(36) Pelikan, M.; Hura, G. L.; Hammel, M. Structure and flexibility within proteins as identified through small angle X-ray scattering. *Gen Physiol Biophys* **2009**, *28* (2), 174-189. DOI: 10.4149/gpb_2009_02_174 From NLM.
(37) Brooks, B. R.; Brooks, C. L., 3rd; Mackerell, A. D., Jr.; Nilsson, L.; Petrella, R. J.; Roux, B.; Won, Y.; Archontis, G.; Bartels, C.; Boresch, S.; et al. CHARMM: the biomolecular simulation program. *J Comput Chem* **2009**, *30* (10), 1545-1614. DOI: 10.1002/jcc.21287 From NLM.
(38) Schneidman-Duhovny, D.; Hammel, M.; Tainer, J. A.; Sali, A. Accurate SAXS profile computation and its assessment by contrast variation experiments. *Biophys J* **2013**, *105* (4), 962-974. DOI: 10.1016/j.bpj.2013.07.020 From NLM.
(39) Schneidman-Duhovny, D.; Hammel, M.; Tainer, J. A.; Sali, A. FoXS, FoXSDock and MultiFoXS: Single-state and multi-state structural modeling of proteins and their complexes based on SAXS profiles. *Nucleic Acids Res* **2016**, *44* (W1), W424-429. DOI: 10.1093/nar/gkw389 From NLM.
(40) Jo, S.; Kim, T.; Iyer, V. G.; Im, W. CHARMM-GUI: a web-based graphical user interface for CHARMM. *J Comput Chem* **2008**, *29* (11), 1859-1865. DOI: 10.1002/jcc.20945 From NLM.
(41) Kozakov, D.; Hall, D. R.; Xia, B.; Porter, K. A.; Padhorny, D.; Yueh, C.; Beglov, D.; Vajda, S. The ClusPro web server for protein-protein docking. *Nat Protoc* **2017**, *12* (2), 255-278. DOI: 10.1038/nprot.2016.169 From NLM.
(42) Jones, G.; Jindal, A.; Ghani, U.; Kotelnikov, S.; Egbert, M.; Hashemi, N.; Vajda, S.; Padhorny, D.; Kozakov, D. Elucidation of protein function using computational docking and hotspot analysis by ClusPro and FTMap. *Acta Crystallogr D Struct Biol* **2022**, *78* (Pt 6), 690-697. DOI: 10.1107/s2059798322002741 From NLM.

(43) Kozakov, D.; Beglov, D.; Bohnuud, T.; Mottarella, S. E.; Xia, B.; Hall, D. R.; Vajda, S. How good is automated protein docking? *Proteins* **2013**, *81* (12), 2159-2166. DOI: 10.1002/prot.24403 From NLM.
(44) Vajda, S.; Yueh, C.; Beglov, D.; Bohnuud, T.; Mottarella, S. E.; Xia, B.; Hall, D. R.; Kozakov, D. New additions to the ClusPro server motivated by CAPRI. *Proteins* **2017**, *85* (3), 435-444. DOI: 10.1002/prot.25219 From NLM.
(45) Desta, I. T.; Porter, K. A.; Xia, B.; Kozakov, D.; Vajda, S. Performance and Its Limits in Rigid Body Protein-Protein Docking. *Structure* **2020**, *28* (9), 1071-1081.e1073. DOI: 10.1016/j.str.2020.06.006 From NLM.
(46) Segal, H. M.; Spatzal, T.; Hill, M. G.; Udit, A. K.; Rees, D. C. Electrochemical and structural characterization of Azotobacter vinelandii flavodoxin II. *Protein Science* **2017**, *26* (10), 1984-1993. DOI: https://doi.org/10.1002/pro.3236 (acccessed 2025/10/08).
(47) Murray, D. T.; Ge, X.; Schut, G. J.; Rosenberg, D. J.; Hammel, M.; Bierma, J. C.; Hille, R.; Adams, M. W. W.; Hura, G. L. Correlating Conformational Equilibria with Catalysis in the Electron Bifurcating EtfABCX of Thermotoga maritima. *Biochemistry* **2023**. DOI: 10.1021/acs.biochem.3c00472 From NLM.
(48) Khan, S. A.; Hicks, A.; Leite, W. C.; Byrnes, J.; Gorai, B.; Mroginski, M.-A.; O'Neill, H.; Miller, A.-F. Extended conformations of bifurcating electron transfer flavoprotein constitute up to half the population, possibly mediating conformational change. *Chemical Science* **2024**, *15* (45), 18796-18817, 10.1039/D4SC04544K. DOI: 10.1039/D4SC04544K.
(49) Demmer, J. K.; Pal Chowdhury, N.; Selmer, T.; Ermler, U.; Buckel, W. The semiquinone swing in the bifurcating electron transferring flavoprotein/butyryl-CoA dehydrogenase complex from Clostridium difficile. *Nat Commun* **2017**, *8* (1), 1577. DOI: 10.1038/s41467-017-01746-3.
(50) Chowdhury, N. P.; Mowafy, A. M.; Demmer, J. K.; Upadhyay, V.; Koelzer, S.; Jayamani, E.; Kahnt, J.; Hornung, M.; Demmer, U.; Ermler, U.; et al. Studies on the mechanism of electron bifurcation catalyzed by electron transferring flavoprotein (Etf) and butyryl-CoA dehydrogenase (Bcd) of Acidaminococcus fermentans. *J Biol Chem* **2014**, *289* (8), 5145-5157. DOI: 10.1074/jbc.M113.521013.
(51) Feng, X.; Schut, G. J.; Lipscomb, G. L.; Li, H.; Adams, M. W. W. Cryoelectron microscopy structure and mechanism of the membrane-associated electron-bifurcating flavoprotein Fix/EtfABCX. *Proceedings of the National Academy of Sciences* **2021**, *118* (2), e2016978118. DOI: 10.1073/pnas.2016978118.
(52) Klugkist, J.; Voorberg, J.; Haaker, H.; Veeger, C. Characterization of three different flavodoxins from Azotobacter vinelandii. *Eur J Biochem* **1986**, *155* (1), 33-40. DOI: 10.1111/j.1432-1033.1986.tb09455.x From NLM.
(53) Yang, Z.-Y.; Ledbetter, R.; Shaw, S.; Pence, N.; Tokmina-Lukaszewska, M.; Eilers, B.; Guo, Q.; Pokhrel, N.; Cash, V. L.; Dean, D. R.; et al. Evidence That the Pi Release Event Is the Rate-Limiting Step in the Nitrogenase Catalytic Cycle. *Biochemistry* **2016**, *55* (26), 3625-3635. DOI: 10.1021/acs.biochem.6b00421.
(54) Lubner, C. E.; Jennings, D. P.; Mulder, D. W.; Schut, G. J.; Zadvornyy, O. A.; Hoben, J. P.; Tokmina-Lukaszewska, M.; Berry, L.; Nguyen, D. M.; Lipscomb, G. L.; et al. Mechanistic insights into energy conservation by flavin-based electron bifurcation. *Nat Chem Biol* **2017**, *13* (6), 655-659. DOI: 10.1038/nchembio.2348.
(55) McComb, R. B.; Bond, L. W.; Burnett, R. W.; Keech, R. C.; Bowers, G. N., Jr. Determination of the molar absorptivity of NADH. *Clin Chem* **1976**, *22* (2), 141-150. From NLM.

(56) Petrova, E. V.; Korotkova, E. I.; Kratochvil, B.; Voronova, O. A.; Dorozhko, E. V.; Bulycheva, E. V. Investigation of Coenzyme Q10 by Voltammetry. *Procedia Chemistry* **2014**, *10*, 173-178. DOI: https://doi.org/10.1016/j.proche.2014.10.030.
(57) Merkley, E. D.; Rysavy, S.; Kahraman, A.; Hafen, R. P.; Daggett, V.; Adkins, J. N. Distance restraints from crosslinking mass spectrometry: Mining a molecular dynamics simulation database to evaluate lysine–lysine distances. *Protein Science* **2014**, *23* (6), 747-759. DOI: https://doi.org/10.1002/pro.2458.
